# Endolysosomes Are the Principal Intracellular Sites of Acid Hydrolase Activity

**DOI:** 10.1016/j.cub.2016.06.046

**Published:** 2016-09-12

**Authors:** Nicholas A. Bright, Luther J. Davis, J. Paul Luzio

**Affiliations:** 1 Cambridge Institute for Medical Research and Department of Clinical Biochemistry, University of Cambridge School of Clinical Medicine, Wellcome Trust/MRC Building, Cambridge Biomedical Campus, Hills Road, Cambridge CB2 0XY, UK

## Abstract

The endocytic delivery of macromolecules from the mammalian cell surface for degradation by lysosomal acid hydrolases requires traffic through early endosomes to late endosomes followed by transient (kissing) or complete fusions between late endosomes and lysosomes. Transient or complete fusion results in the formation of endolysosomes, which are hybrid organelles from which lysosomes are re-formed. We have used synthetic membrane-permeable cathepsin substrates, which liberate fluorescent reporters upon proteolytic cleavage, as well as acid phosphatase cytochemistry to identify which endocytic compartments are acid hydrolase active. We found that endolysosomes are the principal organelles in which acid hydrolase substrates are cleaved. Endolysosomes also accumulated acidotropic probes and could be distinguished from terminal storage lysosomes, which were acid hydrolase inactive and did not accumulate acidotropic probes. Using live-cell microscopy, we have demonstrated that fusion events, which form endolysosomes, precede the onset of acid hydrolase activity. By means of sucrose and invertase uptake experiments, we have also shown that acid-hydrolase-active endolysosomes and acid-hydrolase-inactive, terminal storage lysosomes exist in dynamic equilibrium. We conclude that the terminal endocytic compartment is composed of acid-hydrolase-active, acidic endolysosomes and acid hydrolase-inactive, non-acidic, terminal storage lysosomes, which are linked and function in a lysosome regeneration cycle.

## Highlights

• Late endosome-lysosome fusion creates acidic, cathepsin-active endolysosomes
• Terminal storage lysosomes are cathepsin inactive and not acidic
• Fusion events creating endolysosomes precede the onset of cathepsin activity
• A lysosome regeneration cycle links endolysosomes and terminal storage lysosomes

## Teaser

Bright et al. provide evidence that the terminal endocytic compartment is composed of acid-hydrolase-active, acidic endolysosomes and acid hydrolase-inactive, non-acidic, terminal storage lysosomes, which are linked and function in a lysosome regeneration cycle.

## Introduction

Lysosomes were first discovered by De Duve and colleagues over 60 years ago, and their importance in the degradation of endocytosed, phagocytosed, and autophagocytosed macromolecules was soon recognized [1]. For a long time, lysosomes were simply regarded as endpoint, degradatively active compartments. Their morphological heterogeneity was recognized early, but only over the past two decades have their dynamic, functional interactions with other organelles of the secretory, endocytic, and autophagic pathways been properly appreciated. In addition, it has recently been shown that lysosomes play a central role in nutrient sensing and signaling to the nucleus, to mediate cellular responses to starvation and the regulation of energy metabolism [2, 3, 4, 5]. The delivery of endocytosed macromolecules to lysosomal acid hydrolases for degradation occurs as a result of transient (kissing or kiss-and-run) and complete fusion events between lysosomes and late endosomes/multivescular bodies (MVBs) [6, 7, 8]. A consequence of these transient and complete fusion events is the formation of hybrid organelles, now called endolysosomes [8], which have properties intermediate between late endosomes and lysosomes, and from which lysosomes are re-formed. While the presence of cathepsins in endolysosomes has been shown in cells [9] and cell-free systems by immuno-electron microscopy (immuno-EM); reviewed in [7]), the extent to which endolysosomes or more terminal lysosomal compartments are involved in hydrolysis of macromolecules has not been determined. In the present study, we have identified the late endocytic organelles in which acid hydrolases are catalytically active by using synthetic membrane-permeable cathepsin substrates, which liberate fluorescent reporters that remain within the organelle upon proteolytic cleavage, as well as by acid phosphatase (AcPase) cytochemistry. Using fluorescence microscopy and EM, we observed that endolysosomes are the principal organelles in which substrates are hydrolyzed and that accumulate acidotropic probes. They could be distinguished from terminal storage lysosomes, which are acid hydrolase inactive and do not accumulate acidotropic probes. We have also used live-cell microscopy to study the dynamic interaction of late endosomes, endolysosomes, and acid-hydrolase-inactive terminal storage lysosomes, to determine the relationship of organelle kissing to the development of acid hydrolase activity and to study the re-formation of terminal storage lysosomes from endolysosomes.

## Results

### Fluorescent Cresyl Violet Is Liberated from Hydrolyzed Cathepsin Substrates Only in a Subpopulation of Terminal Endocytic Compartments

**Figure 1:**
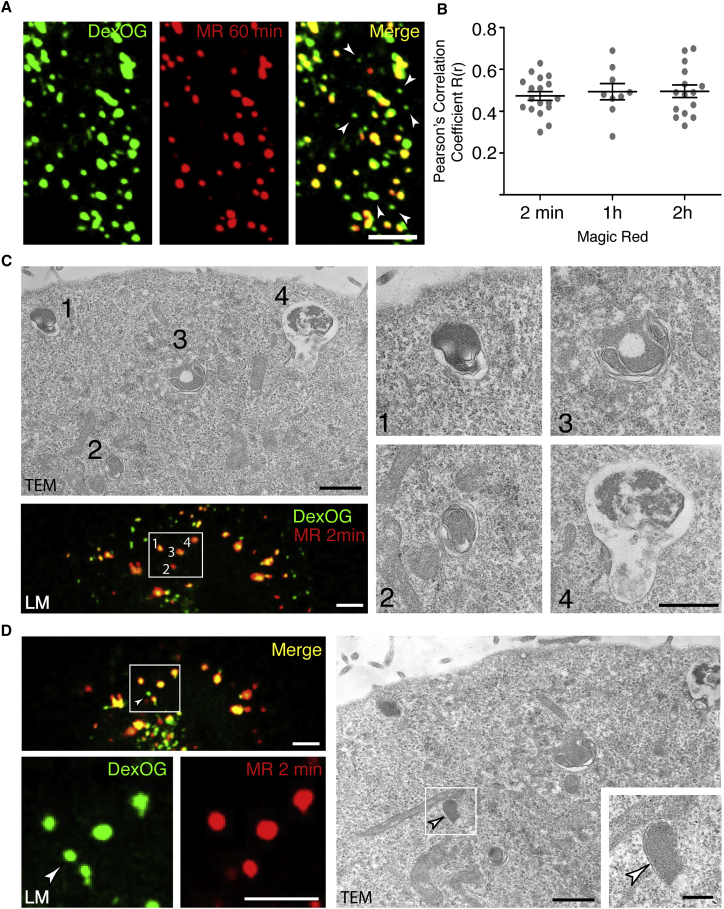
Endolysosomes Are the Principal Sites of Cathepsin B Catalytic Activity Terminal endocytic compartments of NRK cells were loaded with DexOG for 4 hr followed by a 20 hr chase in DexOG-free medium. (A) Part of a single cell showing that cresyl violet fluorescence (red) was present only in a subset of terminal endocytic organelles containing pre-loaded dextran after incubation with cathepsin B MR substrate for 1 hr. Arrowheads show examples where terminal endocytic organelles have not hydrolyzed the MR substrate. (B) Pearson’s correlation coefficients (R(r); mean ± SEM of nine or more cells within a single experiment) for colocalization of pre-loaded DexOG and liberated cresyl violet fluorescence after hydrolysis of cathepsin B MR substrate for different times (see also Figure S1A). (C) CLEM of the cathepsin-active organelles. DexOG-positive, MR-positive organelles were identified by confocal microscopy before processing for TEM to show their ultrastructure. The boxed region of the confocal image and corresponding section in the TEM shows the ultrastructure of four cathepsin-active endolysosomes. The enlargements show that these organelles contain multi-lamellar intralumenal membranes and electron dense content. (D) An image from an adjacent confocal plane to that shown in (C) with enlargements and corresponding TEM image of the boxed region identifying a DexOG-positive, MR-negative terminal endocytic organelle (arrowhead) within the constellation of endolysosomes. The TEM image of this organelle reveals it to be a granular dense core lysosome (boxed region of TEM image and inset). Scale bars represent 5 μm (A), 5 μm (C, light microscopy [LM]), 1 μm (C, TEM), 500 nm (C, TEM enlargements), 5 μm (D, LM), 1 μm (D, TEM), and 200 nm (D, TEM inset). See also Figure S1.

Terminal endocytic compartments of cultured normal rat kidney (NRK) fibroblasts were first loaded with a DexOG (dextran-Oregon green 488) conjugate using a 4 hr pulse/20 hr chase protocol to establish an equilibrium of labeling between lysosomes and endolysosomes as previously described [6]. The cells were then incubated with the membrane permeable cathepsin B MR (Magic Red) substrate that, upon hydrolysis, liberates membrane-impermeable fluorescent cresyl violet within organelles containing catalytically active cathepsin B [10, 11]. Using confocal microscopy either of live cells or after fixing cells with paraformaldehyde, the liberation of cresyl violet fluorescence in some of the DexOG-positive organelles was detected within 30 s of the addition of cathepsin B MR substrate (data not shown). Even after 60 min, with cathepsin B MR substrate present in the medium throughout, only some of the DexOG-positive compartments were labeled (Figures 1A and S1A). There was no significant difference in the overall colocalization of cresyl violet and DexOG between cells incubated with the cathepsin B MR substrate for 2 min or 2 hr (Figure 1B). To visualize the ultrastructure of the cresyl violet-containing organelles, we carried out correlative light and electron microscopy (CLEM) after incubation with cathepsin B MR substrate for 2 min, to ensure that the population of cresyl violet-containing (MR-positive) organelles included few, if any, re-formed lysosomes. The majority of the MR-positive organelles were characterized by the presence of multi-lamellar intralumenal membranes and an electron-dense lumenal content containing electron-lucent sub-domains, but we also observed electron-lucent vacuolar organelles containing intralumenal membrane, intralumenal vesicles (ILVs) and diffuse electron-dense content (Figure 1C). Thus, there was a diverse array of ultrastructural morphology among the organelles demonstrating acid hydrolase activity. In contrast, Figure 1D shows a different confocal plane to that illustrated in Figure 1C, revealing an organelle containing DexOG, but not cresyl violet which, in transmission electron microscopy (TEM), possessed the characteristic electron dense, granular morphology of a dense core lysosome. The smaller size of this dense core lysosome compared to the MR-positive organelles is consistent with data from confocal microscopy showing the size distribution of MR-positive and MR-negative terminal endocytic compartments (Figure S1B).

To confirm that our observations were neither cell type dependent nor specific for only one cathepsin, we repeated our confocal microscopy experiments with HeLa and MCF7 cells as well as with a cathepsin L MR substrate (Figures S1C–S1E). In all cases, only some of the fluorescent dextran-positive terminal endocytic compartments were MR positive. We interpreted the MR experiments combined with the CLEM as showing two types of terminal endocytic compartment: cathepsin-active organelles (predicted to be endolysosomes) and cathepsin-inactive terminal lysosomes. In our confocal fluorescence microscopy experiments, we assessed colocalization by calculating Pearson’s and Manders’ coefficients and used the latter (see the Supplemental Experimental Procedures), to estimate the proportion of fluorescent dextran-positive terminal endocytic compartments that were cathepsin-active endolysosomes as 67.4% ± 4.6% in NRK cells, 46.8% ± 8.7% in HeLa cells, and 64.1% ± 3.3% in MCF7 cells (see values for M1 in Figure S1E). The remainder of the fluorescent dextran-positive terminal endocytic compartments were cathepsin-inactive terminal lysosomes. The lack of hydrolysis of cathepsin MR substrates in the cathepsin-inactive terminal lysosomes was not due to a problem of substrate access or the absence of enzyme in these compartments, since incubation of the cells with the cathepsin B MR substrate in a pH 5 buffer in the presence of nigericin and monensin to equilibrate pH across compartments allowed us to capture images of the appearance of cresyl violet fluorescence in all of a cell’s DexOG-positive terminal endocytic compartments before it dissipated across the organelles’ limiting membranes within 5 min (Figure S1F; see also immuno-EM of cathepsin D in MCF7 cells below).

**Figure 2:**
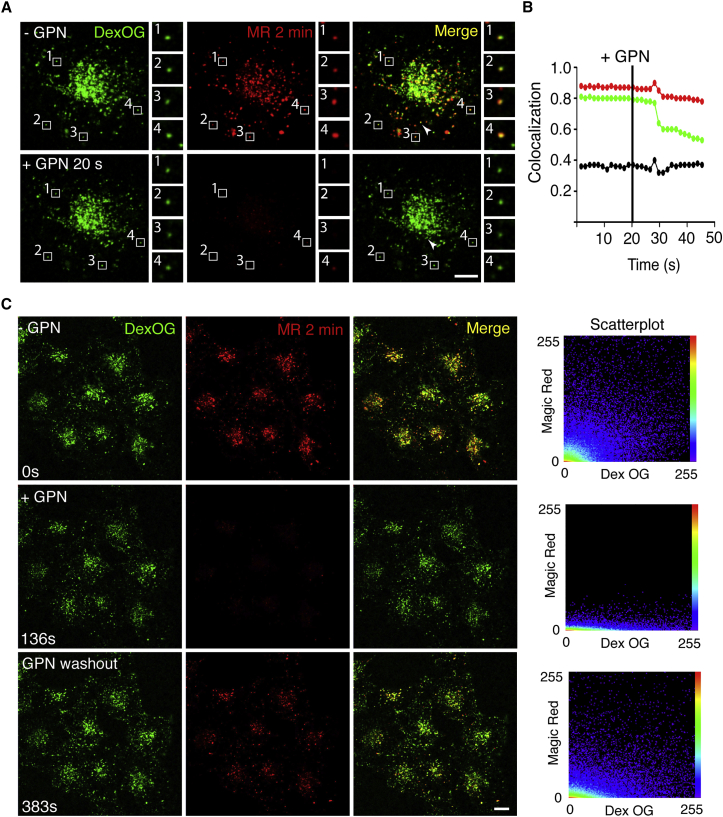
Magic-Red-Positive Endolysosomes Contain Active Cathepsin C and Can Recover Enzyme Activity after GPN Treatment Terminal endocytic compartments of NRK cells were loaded with DexOG for 4 hr followed by a 20 hr chase in DexOG-free medium. (A) Cathepsin-active endolysosomes were revealed by incubation with cathepsin B MR substrate for 2 min, and images of the living cells were collected on the confocal microscope (top, single cell with enlargements of four endolysosomes). Addition of 200 μM GPN (bottom) resulted in rapid dissipation of cresyl violet from the cathepsin-active endolysosomes (loss of cresyl violet from four endolysosomes shown in enlargements) but the DexOG (Mr 10,000) was retained in these organelles. See also Figure S2A and Movie S1. The motility of transiently permeabilized endolysosomes (e.g., the organelle identified by arrowhead) was unaffected (see Movie S1). (B) Pearson’s (R(r); black) and Manders’ (M1, green, DexOG:MR and M2, red, MR:DexOG) correlation coefficients for colocalization of pre-loaded DexOG and liberated cresyl violet fluorescence (MR) before and after addition (vertical black line) of 200 μM GPN to the cell shown in (A) and Movie S1. (C) Cathepsin-active endolysosomes were revealed by incubation with Magic Red (MR) substrate for 2 min (top row), and images of the living cells were collected on the confocal microscope. Addition of 200 μM GPN (middle row) resulted in rapid dissipation of cresyl violet from the cathepsin-active endolysosomes, but the DexOG was retained as described above. Washout of the GPN with medium containing cathepsin B MR substrate (bottom) showed that cathepsin B was retained and remained catalytically active upon removal of GPN. The associated scatterplots of the merged images clearly show the dissipation and re-acquisition of cresyl violet fluorescence upon addition of GPN and subsequent washout. See also Movie S3. Scale bars represent 10 μm. See also Figure S2.

Further evidence for a distinction between cathepsin-active endolysosomes and inactive terminal lysosomes came from experiments with the membrane permeable cathepsin C substrate GPN (glycyl-D-phenylalanine-2-naphthylamide) [12]. When GPN is added to cultured cells, its hydrolysis in organelles in which cathepsin C is catalytically active causes a transient permeabilization of the limiting membrane and the leak of small molecular mass solutes (relative molecular weight [Mr] <10,000) [13, 14, 15, 16]. In NRK cells in which terminal endocytic compartments were pre-loaded with DexOG, we observed a rapid dissipation of cresyl violet (Mr 321), but not DexOG (Mr 10,000) from the endolysosomes when GPN was added after the cathepsin B MR substrate (Figures 2A, 2B, and S2A; Movie S1). The GPN treatment also failed to dissipate DexOG from the cathepsin-inactive terminal lysosomes. In addition, GPN treatment of NRK cells in which terminal endocytic compartments were pre-loaded by fluid phase endocytosis of Lucifer Yellow (LucY, Mr 457) showed loss of both cresyl violet and LucY from the MR-positive endolysosomes, but not from the MR-negative terminal endocytic compartments (Movie S2; Figure S2B). The transient nature of the GPN effect allowed us to show that following washout of the GPN, individual endolysosomes, which had leaked cresyl violet, rapidly regained the ability to hydrolyze the cathepsin B MR substrate (Figure 2C; Movie S3).

### Cathepsin-Active Endolysosomes Are Acid Phosphatase Positive and Can Degrade Endocytosed Macromolecules

**Figure 3:**
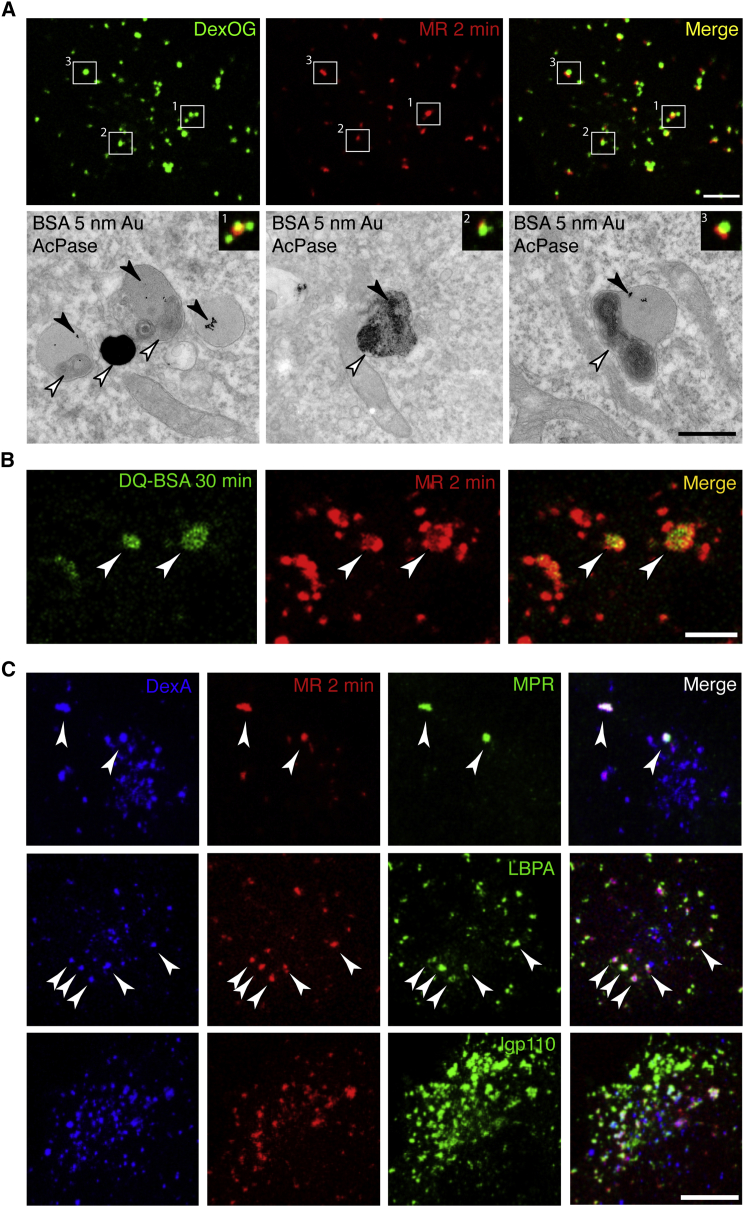
Characterization of Endolysosomes (A) Terminal endocytic compartments of NRK cells were loaded with DexOG and BSA-gold for 4 hr followed by a 20 hr chase in DexOG/BSA-gold-free medium and incubation with cathepsin B MR substrate for 2 min. DexOG/MR-positive compartments were identified by confocal microscopy (top) and CLEM with AcPase cytochemistry, performed using β-glycerophosphate and cerium chloride capture for 2 hr prior to processing for TEM. This revealed cerium phosphate deposition in AcPase-active organelles (bottom, white arrowheads). BSA-gold (bottom, black arrowheads) labeled catalytically active endolysosomes and AcPase-inactive granular, dense core lysosomes; see also Figure S3C). (B) Confocal fluorescence microscopy of NRK cells that had endocytosed DQ-BSA for 30 min and were then incubated with cathepsin B MR substrate for 2 min. Proteolytically cleaved DQ-BSA is indicated by white arrowheads and colocalizes with cresyl violet (MR). (C) Confocal immunofluorescence microscopy of MR-positive organelles (red) in NRK cells pre-loaded with DexA (blue), to label terminal endocytic compartments and immunolabeled with antibodies against the cation-independent mannose 6-phosphate receptor (MPR), lysobisphosphatidic acid (LBPA), or lysosomal glycoprotein 110 (lgp110) followed by Alexa-488-conjugated secondary antibodies (green). Examples of cathepsin B catalytically active organelles labeled with antibodies to MPR and LBPA indicated by arrowheads. Extensive colocalization of both DexA and cresyl violet (MR) with lgp110 was observed. Scale bars represent 10 μm (A, top), 500 nm (A, bottom TEM), and 10 μm (B and C). See also Figure S3.

CLEM was used to examine whether the MR-positive compartments in NRK cells were also AcPase positive by applying EM cytochemistry. For these experiments, terminal endocytic compartments of NRK cells were loaded with DexOG and BSA-gold (BSA conjugated to 5 nm colloidal gold) [9] before the cells were incubated with the cathepsin B MR substrate for 2 min. As expected, the MR-positive endolysosomes were labeled with BSA-gold and these compartments were also AcPase-positive (Figure 3A). The AcPase-positive endolysosomes, which account for 61.8% ± 5.7% (three experiments) of the BSA-gold positive organelles were also positive for cation-independent mannose 6-phosphate receptor (MPR) and lysobisphosphatidic acid/bis(monoacylglycerol)phosphate (LBPA/BMP) by immuno-EM (Figures S3A and S3B). They were sometimes closely associated with AcPase-negative, BSA-gold-positive terminal lysosomes (Figure 3A, box 1, lower-left panel, and Figure S3C). This association is consistent with observations from previous live-cell experiments in which several lysosomes were often seen very close to endolysosomes formed by earlier kissing events between endosomes and lysosomes (see, e.g., Movie 2 in [6]). Although we saw many instances in which AcPase reaction product was distributed throughout a BSA-gold-positive organelle (Figure 3A, box 2, lower-middle panel), we sometimes observed a region of AcPase reaction product within the same limiting membrane as an electron dense BSA-gold-positive region, as if a terminal AcPase-negative lysosome had recently fused with an endolysosome (Figure 3A, box 3, lower-right panel). The co-existence of AcPase-positive and AcPase-negative regions within the same endolysosome limiting membrane, despite the AcPase reaction being carried out at acid pH, could also imply that there are luminal regions in which the enzyme is present although inactive, but this was not explored further. Images showing that only some BSA-gold-positive organelles were AcPase positive were also obtained from MCF7 cells (Figure S3D). The availability of an antibody to human cathepsin D that was usable for immuno-EM enabled us to show that terminal endocytic organelles containing BSA-gold in MCF7 cells also contained immunoreactive cathepsin D irrespective of whether they were AcPase positive or negative, with 62.8% ± 1.5% (three experiments) of the immunoreactive cathepsin D molecules associated with BSA-gold-positive organelles being in the AcPase-negative lysosomes (Figure S3D).

To confirm that the MR-positive endolysosomes are the site of hydrolysis of endocytosed macromolecules, we incubated NRK cells with DQ-BSA (DQ Green BSA), an endocytic probe that becomes fluorescent upon proteolytic cleavage [11, 17], before adding the cathepsin B MR substrate to the culture medium. Green fluorescent signal from the hydrolyzed DQ-BSA was first detected after 15 min of endocytic uptake (data not shown) and was clearly observed by 30 min (Figure 3B). The overlap of the green fluorescent signal with the cresyl violet increased when the cells were incubated with DQ-BSA for longer times (data not shown) and was consistent with the MR-positive endolysosomes being the site of proteolytic degradation of the endocytosed DQ-BSA.

### Cathepsin-Active Endolysosomes Contain Markers Characteristic of Both Late Endosomes and Lysosomes

Fluorescence from the cresyl violet product of cathepsin MR substrate hydrolysis remains clearly visible for only 45–60 min after paraformaldehyde fixation. Nevertheless, we were able to use triple-labeling fluorescence confocal microscopy to characterize further the MR-positive terminal endocytic compartment by applying a rapid immunolabeling protocol. NRK cell terminal endocytic compartments were loaded with dextran Alexa (DexA) 647, and the cells then were incubated with the cathepsin B MR substrate for 2 min. After fixation and permeabilization, the cells were immunolabeled with antibodies specific for MPR, LBPA, and rat lysosomal glycoprotein110 (lgp110). There was extensive colocalization of cresyl violet with all three of these markers (Figure 3C), although the fading of the cresyl violet fluorescence after fixation prevented robust quantitative assessment of its colocalization with the antibodies used for immunolabeling. MPR has often been used to distinguish endosomes from lysosomes [18, 19], LBPA has been described as pre-lysosomal [20] and lgp110, like other lysosomal membrane glycoproteins [18] is regarded as a marker of both late endosomes and lysosomes. Using live-cell confocal fluorescence microscopy of HeLa cells stably expressing EGFP-tagged Rab5, Rab7, or Rab9, we observed that MR-positive organelles were negative for the early endosomal marker Rab5, but positive for the late endosomal markers Rab7 and Rab9 (Figure S3E). Taken together, our data showed that the MR-positive organelles demonstrated characteristics of both endosomes and lysosomes, consistent with them being endolysosomes.

### Acidotropic Probes Accumulate in Endolysosomes, but Not in Terminal Lysosomes

**Figure 4:**
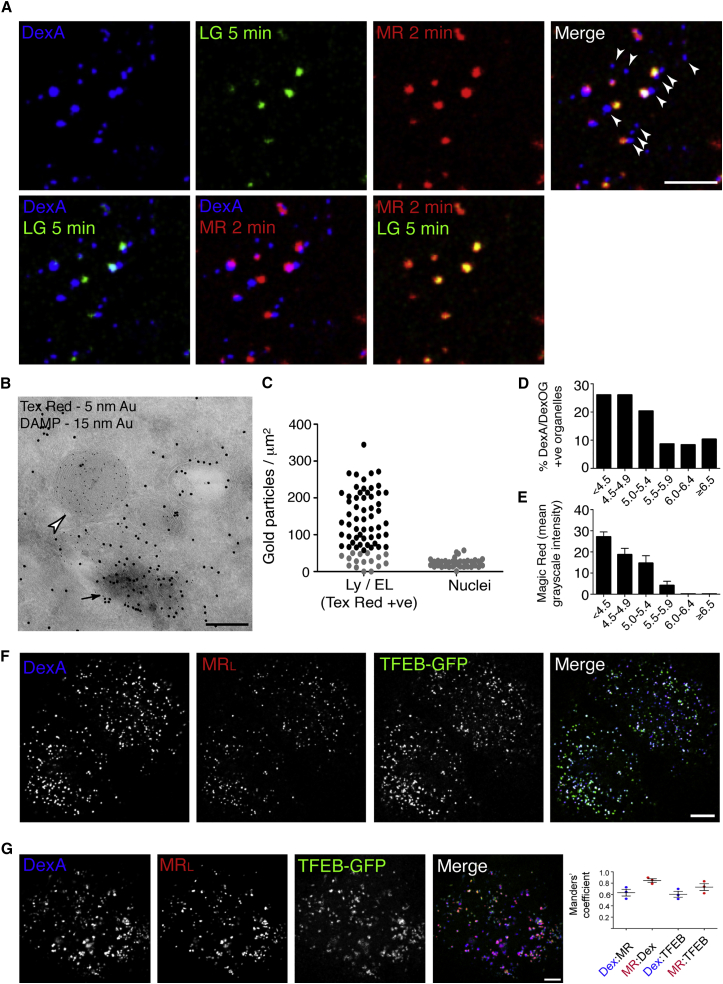
Cathepsin-Active Endolysosomes Accumulate Acidotropic Molecules but Terminal Lysosomes Do Not (A) Confocal fluorescence microscopy images of cathepsin-active, acidic endolysosomes in a single NRK cell, in which terminal endocytic compartments were pre-loaded with DexA, revealed by incubation with cathepsin B MR substrate for 2 min followed by addition of 50 nM LysoTracker Green (LG) for 5 min. The merge shows that some DexA-positive compartments were both MR negative and LG negative (white arrowheads, see also Figure S4). (B) Immuno-EM of DAMP accumulation in acidic organelles. NRK cells were pre-loaded with DexTR (5 nm gold) to label terminal endocytic compartments before incubation with 30 μM DAMP (15 nm gold) for 30 min to identify acidic organelles. An endolysosome has accumulated the acidotropic DAMP (arrow), but the adjacent terminal lysosome (arrowhead) has not. (C) Quantitation of DAMP immunolabeling in endolysosomes and lysosomes that had accumulated DexTR. The heterogeneity of DAMP accumulation in individual organelles in a representative experiment is shown, with 22.5% of the DexTR-loaded compartments having only accumulated DAMP to the same degree as nuclei (gray points). (D) Heterogeneity of pH in terminal endocytic compartments determined by ratiometric imaging using DexOG and DexA. Representative experiment is shown in which the pH of 300 organelles was measured. (E) Cathepsin B activity, determined by incubation with cathepsin B MR substrate, in terminal endocytic compartments with different pH measured by ratiometric imaging (mean ± SEM). Same experiment as in (D). (F) Confocal fluorescence microscopy images of NRK cells stably expressing TFEB-GFP, pre-loaded with DexA to label terminal endocytic compartments, incubated with cathepsin L MR substrate (MRL) for 3 min and fixed after cytosol washout. (G) Confocal fluorescence microscopy images of NRK cells stably expressing TFEB-GFP, pre-loaded with DexA, to label terminal endocytic compartments, incubated with 250 nM Torin1 for 3 hr at 37°C, then with cathepsin L MR substrate (MRL) for 2 min before imaging. Graph to the right shows Manders’ correlation coefficients (mean ± SEM; three experiments). Scale bars represent 10 μm (A), 200 nm (B), 10 μm (F), and 5 μm (G). See also Figure S4.

Late endocytic organelles have been widely shown to accumulate acidotropic probes, which are membrane-permeable weak bases that have helped to define these organelles as having an acidic lumen [18, 21]. As expected, the MR-positive endolysosomes in NRK cells also accumulated the acidotropic probe LysoTracker Green (LG) when the cells were incubated with the cathepsin B MR substrate followed by the LG (Figures 4A, S4A, and S4B). However, if cells were first allowed to accumulate LG followed by incubation with the cathepsin B MR substrate, there was a failure to hydrolyze the substrate and liberate cresyl violet (data not shown), consistent with the accumulation of LG causing alkalinization of the organelle lumen and a sub-optimal pH for cathepsin B activity. In contrast to the endolysosomes, terminal lysosomes, which were DexA positive but MR negative, were unable to accumulate LG (Figures 4A and S4A). Even after incubation with LG for 60 min, large numbers of dextran-loaded, terminal endocytic organelles did not accumulate the acidotropic molecule (Figure S4A). Similar colocalization results were obtained when NRK, HeLa, and MCF7 cells, containing late endocytic compartments pre-loaded with DexA, were compared after incubation with the cathepsin L MR substrate for 2 min and LG for 5 min. These results were consistent with all three cell types having terminal lysosomes that were not acidic and therefore unable to accumulate the acidotropic probe LG (Figures S4C and S4D).

We confirmed the presence of acidic and non-acidic late endocytic compartments by EM, utilizing the fixable cell-permeant, acidotropic reagent DAMP (3-[2,4-dinitroanilino]-3′-amino-N-methyldipropylamine) [22]. NRK cells with terminal endocytic compartments pre-loaded with dextran-Texas red (DexTR) were incubated with 30 μM DAMP for 30 min prior to aldehyde fixation. The cells were processed for immuno-EM using antibodies against Texas red and DAMP. DAMP accumulated in some, but not all, of the late endocytic organelles (Figure 4B). Quantification of DAMP accumulation in the entire DexTR-positive compartment showed a wide range of accumulation consistent with heterogeneity of luminal pH across the organelles of the dextran-loaded late endocytic compartment. In a representative experiment, 22.5% of the DexTR-positive organelles accumulated DAMP to the same extent as nuclei and therefore had a neutral luminal pH (Figure 4C). Further validation of the heterogeneity of luminal pH in dextran-loaded terminal endocytic compartments was obtained using ratiometric imaging after loading with pH-sensitive DexOG and pH-insensitive DexA. In a representative experiment 18% of dextran-loaded organelles had pH values >6.0 (Figure 4D), and few organelles with a pH value >5.5 showed any significant cathepsin B activity (Figure 4E). Overall, our observations were consistent with acid-hydrolase-active endolysosomes being acidic and acid-hydrolase-inactive, dense core, terminal lysosomes being neutral.

### Transcription Factor TFEB Localizes to Both Endolysosomes and Terminal Lysosomes

The observation of a range of acidity in late endocytic compartments, including the presence of non-acidic lysosomes, led us to examine the localization of GFP-tagged TFEB (TFEB-GFP) in NRK cells stably expressing this protein. This was because of the reported role of the vacuolar ATPase (V-ATPase) in sensing the physiological and nutritional status of the cell and its upstream function in determining the phosphorylation status of TFEB and whether it traffics to the nucleus to activate transcription [4]. After releasing the cytosolic pool of TFEB-GFP by saponin-permeabilization of the plasma membrane, we observed that membrane-associated TFEB-GFP was present on both MR-positive and -negative terminal endocytic organelles containing fluorescent dextran (Figure 4F). Background fluorescence after plasma membrane permeabilization precluded quantitative analysis, and we therefore also assessed the localization of the TFEB-GFP remaining on endolysosomes and lysosomes after torin treatment. By inhibiting mTORC1, torin prevents the phosphorylation of TFEB causing rapid translocation of the cytosolic pool into the nucleus (see below) but also results in lysosomal accumulation of TFEB that is bound to inactive mTORC1 and no longer able to exchange with the cytosol [4]. Following torin treatment of NRK cells expressing TFEB-GFP, membrane-associated TFEB-GFP was observed on both MR-positive endolysosomes and MR-negative terminal lysosomes, confirmed by the similarity of the Manders’ colocalization coefficients for overlap of TFEB-GFP with MR and fluorescent dextran (Figure 4G).

### Organelle Kissing Nucleates Activation of the Acid Hydrolases

**Figure 5:**
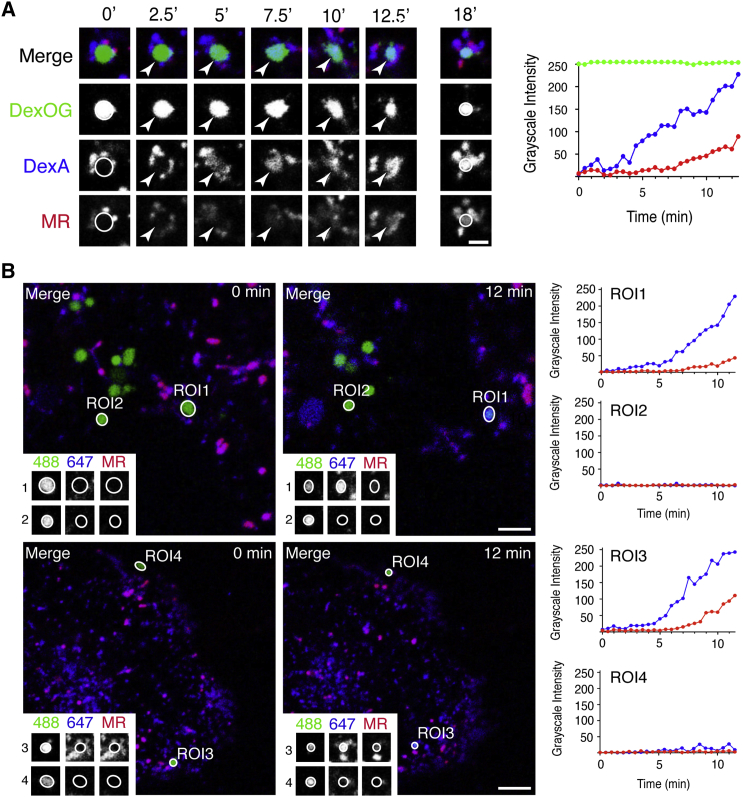
Organelle Kissing Causes Cathepsin Activation in the Newly Forming Endolysosome Terminal endocytic compartments of NRK cells were pre-loaded with DexA for 4 hr followed by a 20-hr chase in DexA-free medium, and then late endosomes were loaded with DexOG by uptake for 10 min followed by a 5 min chase in DexOG-free medium containing cathepsin B MR substrate. (A) Time-lapse confocal microscopy of the living cells for 12.5 min, showing that transient fusion of a DexOG-positive late endosome with DexA-laden terminal endocytic compartments resulted in the gradual acquisition of DexA (blue) in the DexOG-positive organelle (green) and a subsequent rise in cresyl violet fluorescence from the cleaved MR substrate (red), also demonstrated (right) by the mean grayscale intensity profile of each fluorochrome in the identified organelle. (B) Further examples of organelles tracked after the addition of cathepsin B MR substrate. The graphs to the right show that DexOG-laden late endosomes (green, 488), which acquired DexA (blue, 647) after transient fusions with terminal endocytic organelles both exhibited a subsequent rise in cresyl violet fluorescence liberated from cleaved MR substrate (red; region of interest 1 [ROI1] and ROI3), but organelles that did not acquire DexA from terminal endocytic compartments during the time course also failed to acquire cresyl violet fluorescence (ROI2 and ROI4). Scale bars represent 10 μm. See also Figure S5.

Given our evidence for a population of dense core, terminal lysosomes, which have a neutral luminal pH and contain immunoreactive but hydrolytically inactive cathepsins, we hypothesized that transient or complete fusion of terminal lysosomes and late endosomes should result in the creation of an appropriate intraorganelle endolysosomal environment to stimulate cathepsin activity. To test this hypothesis, we used a live-cell content mixing assay to investigate whether cathepsin B activity is activated when endosomes exchange content with acid-hydrolase-inactive terminal lysosomes. NRK cell terminal lysosomes were pre-loaded with DexA and late endosomes with DexOG as previously described [6] and the cells observed when incubated with cathepsin B MR substrate in the surrounding medium. We searched for fusion events between DexA-positive, MR-negative lysosomes and DexOG-positive, MR-negative endosomes. These fusion events (see e.g., fusion in Figure S5) were less frequently observed than kissing and fusion between DexA-positive, MR-positive endolysosomes with DexOG-positive, MR-negative endosomes. More often, when searching for interactions between MR-negative endosomes with MR-negative lysosomes, we also observed the close apposition of one or more MR-positive endolysosomes. Nevertheless, the appearance of the lysosomal DexA in a DexOG-positive endosome always commenced before the appearance of cresyl violet fluorescence in the newly forming endolysosome (Figures 5A and 5B; Movie S4). After fusion, we often saw the emergence of tubular budding profiles, containing both fluorescent dextran and cresyl violet, from the resultant endolysosomes (Figure S5; Movie S5), consistent with the tubular budding profiles observed previously after endosome-lysosome kissing and fusion (see, e.g., Figure 1C in [6]. Tubules often retracted back into the endolysosome from which they had emerged, but some broke off, potentially maturing to become re-formed lysosomes (Figure S5).

### Cathepsin-Active Endolysosomes and Inactive Terminal Lysosomes Exist in Dynamic Equilibrium

**Figure 6:**
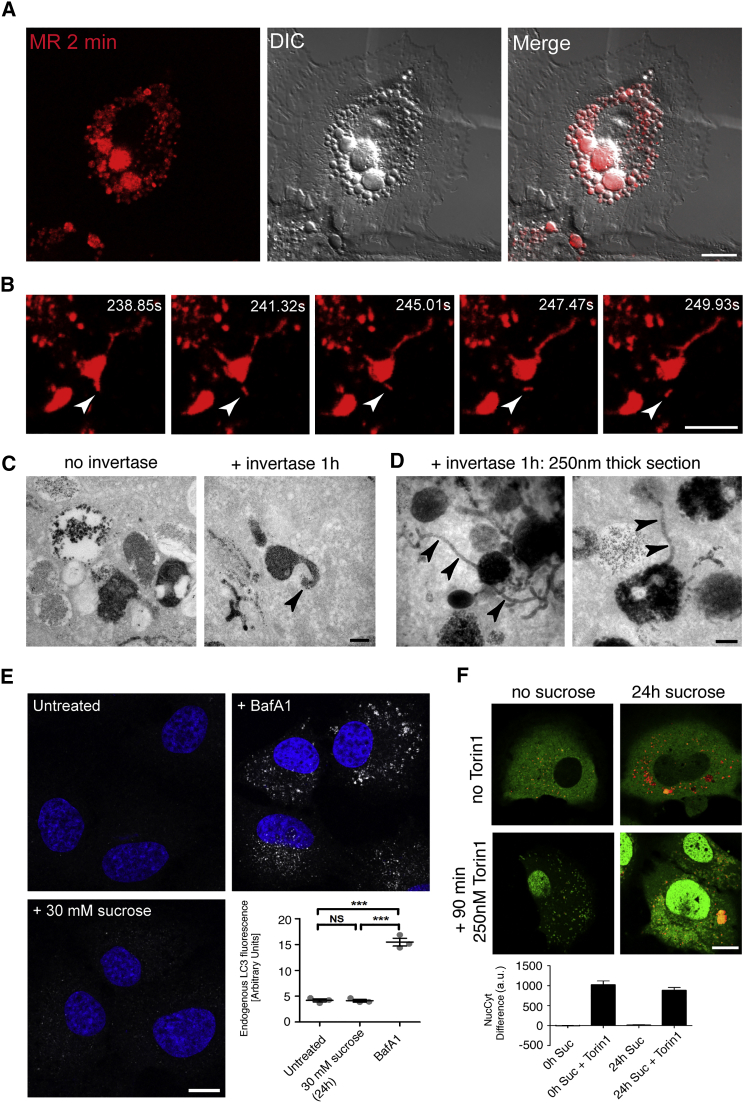
Sucrosomes Are Swollen Endolysosomes from which Lysosomes Can Be Re-formed Sucrosomes were formed in NRK cells by endocytosis of 30 mM sucrose for 24 hr. (A) Incubation of the cells with cathepsin B MR substrate for 2 min showed that the swollen vacuoles seen by differential interference contrast (DIC) microscopy were cathepsin active (MR). (B) NRK cells containing sucrosomes were incubated with medium containing 0.5 mg/ml invertase for 1 hr followed by the addition of cathepsin B MR substrate for 2 min. A time-lapse series of images collected on the confocal microscope showed that addition of invertase resulted in extensive tubulation of the cathepsin-active sucrosomes and in some instances tubules could be observed detaching from the parent sucrosome (arrowhead). See also Movie S6. (C) Following incubation with invertase as in (B), NRK cells containing sucrosomes were fixed for TEM and AcPase cytochemistry. Consistent with cleavage of the cathepsin B MR substrate, cerium phosphate deposition was seen in sucrosomes and tubules (arrowhead). (D) When 250-nm-thick sections were prepared from the cells shown in (C), the extent of cathepsin-active tubules (arrowheads) emanating from sucrosomes was more clearly revealed. (E) Anti-LC3 immunoreactivity was used as a marker of autophagosome accumulation in NRK cells following sucrosome formation (400 nM bafilomycin A1 [BafA1] for 8 hr was a control for autophagosome accumulation). Nuclei were identified by Hoechst staining and mean LC3 immunoreactivity intensity quantified (mean ± SEM; three experiments), following confocal microscopy. ^∗∗∗^p < 0.001 (Student's t test). (F) NRK cells stably expressing TFEB-GFP (green) were incubated with or without 30 mM sucrose (Suc) for 24 hr and subsequently with or without 250 nM Torin 1 for 90 min (as a control for TFEB-GFP translocation to the nucleus), before addition of cathepsin B MR substrate to show cathepsin-active organelles (red). The NucCyt difference plotted (mean ± SEM; three experiments) is the mean fluorescence intensity in the nuclei (Nuc) after subtracting the mean intensity of pixels in the cytoplasm (Cyt) in a.u. Scale bars represent 10 μm (A and B), 200 nm (C and D), and 10 μm (E and F). See also Figure S6.

We have previously shown in NRK cells that the swelling of terminal endocytic organelles following uptake of undigestible sucrose to form sucrosomes is explained by the swelling of compartments, which can be morphologically defined as endolysosomes and are able to recruit all of the terminal dense core lysosomes over a period of several hours [9]. Subsequent endocytic uptake of invertase results in collapse and disappearance of the sucrosomes and re-formation of dense core lysosomes, such that the normal equilibrium of endolysosomes and dense core lysosomes is re-established (see Figure 8 in [9]). In the present study, using NRK cells in which terminal endocytic compartments were pre-loaded with DexA, we observed that, following incubation with sucrose, the sucrosomes were MR-positive and could accumulate LG (Figures 6A, S6A, and S6B), confirming that they were swollen endolysosomes. Subsequent fluid phase endocytic uptake of invertase resulted in MR-positive tubules emanating from these endolysosomes, some of which broke off (Figures 6B, S6C, and S6D; Movie S6). The sucrosomes were AcPase positive when examined by TEM (Figure 6C) as were the tubules emanating from sucrosomes after invertase treatment (Figures 6C and 6D).

In other cell types, sucrose treatment has been shown to stimulate autophagy in a time- and sucrose concentration-dependent manner, causing an accumulation of autophagosomes [23] and also to result in the nuclear translocation of the lysosome-associated transcription factor TFEB (transcription factor EB) ([2] and our unpublished data on MCF7 cells). If that were also the case in NRK cells, it may have complicated the interpretation of our sucrosome experiments. However, under the conditions of our experiments, treatment of NRK cells with 30 mM sucrose for 24 hr caused no significant accumulation of LC3-positive autophagosomes (Figure 6E), and, in an NRK cell line stably expressing TFEB-GFP, there was no significant translocation of TFEB to the nucleus (Figure 6F).

## Discussion

**Figure 7:**
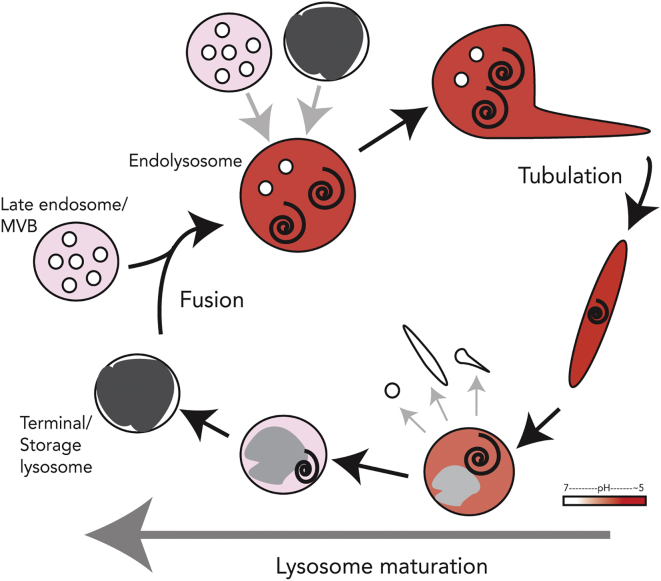
The Lysosome Regeneration Cycle Scheme showing fusion of terminal storage lysosomes with late endosomes to form endolysosomes and the subsequent tubulation, maturation, and content condensation processes required for the re-formation of terminal storage lysosomes. Endolysosomes can undertake further fusions with late endosomes and/or terminal storage lysosomes. Acidic compartments are shaded pink (slightly acidic) to dark red (pH ∼5). Electron-dense content is shaded dark gray.

Our data show that in mammalian cells endolysosomes are the principal sites of intracellular acid hydrolase activity and can be distinguished from acid-hydrolase-inactive terminal lysosomes, which act as a store of acid hydrolases. We have shown in three widely studied mammalian cell lines that endolysosomes, but not lysosomes, contain active cathepsins B, C, and L, as well as AcPase. We observed that the acid-hydrolase-active endolysosomes and inactive terminal lysosomes are in dynamic equilibrium, linked in a lysosome regeneration cycle (Figure 7), consistent with previous studies showing the transient and complete fusion of late endosomes/endolysosomes with terminal lysosomes as well as the process of lysosome re-formation [6, 9, 24]. Our present data, based on the localization of hydrolase activity, show that a higher proportion of organelles in the lysosome regeneration cycle are endolysosomes compared with previous estimates based on morphological or marker localization criteria [9, 25]. The concept that endocytosed macromolecules are delivered to lysosomal enzymes through the formation of acid-hydrolase-active endolysosomes, as a result of transient and complete fusion events, is very different to such compartments simply being intermediates in a linear transport route between endosomes and inactive terminal storage lysosomes, with or without recycling pathways [26, 27]. The live-cell experiments, in which we imaged and recorded individual organelles, showed that fusion events, resulting in the formation of endolysosomes, preceded the onset of acid hydrolase activity. These experiments were not designed to provide detailed information about the time course and kinetics of delivery of endocytosed ligands from the cell surface to acid hydrolases, because we did not use an endocytic pulse, such as could be achieved with a receptor-binding ligand. Although ours is the first study formally demonstrating acid hydrolase activity in the endolysosomal hybrid organelle, the data obtained are consistent with previous observations of acid hydrolase and protein degradation activity in intracellular compartments with endosomal characteristics [18, 28, 29]. We also note, in particular, that the endolysosomal compartment that we have defined in NRK cells has many of the characteristics of the pre-lysosomal compartment previously described in the same cell type [18, 19].

A key feature of the hydrolase-inactive terminal lysosomes observed in the present study is that not only do they contain inactive acid hydrolases, but they can all be recruited to the endolysosome compartment and are re-formed from it. Thus, they are not inert residual bodies or post-lysosomes, nor simply a storage or accumulation compartment for slowly and/or nondegradable material [30]. In contrast, they appear to be readily usable storage organelles for mature acid hydrolases of a type previously proposed [31, 32], with the endolysosome representing the cell’s stomach for digestion of endocytosed macromolecules. The observation that terminal storage lysosomes do not accumulate acidotropic reagents implies that these compartments are not acidic. The existence of non-acidic subsets of lysosomes has been reported previously, leading to the proposal that lysosomal pH fluctuates to allow different degradative processes to occur in sequence with some being mediated by lysosomal enzymes that are inactive in the acidic pH range [33]. In addition, a non-acidic post-lysosomal compartment that is cathepsin D negative by immunostaining depends on the accumulation of non-digestible material for its formation and is made up of inert residual bodies has been described [34]. In contrast to this latter report, the hydrolase-inactive terminal storage lysosomes that we observed in NRK and MCF7 cells do contain cathepsins by both the criteria of activation of hydrolase activity when the intra-organelle pH is equilibrated to pH 5 with ionophores and immuno-EM. Our data are consistent with a recent report from Johnson et al. [35] that several cell types have a subpopulation of lysosomes with pH values >6. However, in contrast to our proposal that the pH of endolysosomes and lysosomes is linked to their position in the lysosome regeneration cycle, Johnson et al. suggested that it is a consequence of their subcellular location, with more peripheral lysosomes being less acidic. Johnston et al. also found that less acidic, peripheral lysosomes have more limited access to material exported by the biosynthetic pathway, but the existence of inactive cathepsins in terminal lysosomes in our experiments implies that the major reason for loss of acid hydrolase activity in these lysosomes is the increase in pH.

Several mechanisms may be functioning together to result in terminal lysosomes having a higher pH than endolysosomes, although the relative importance of each is not yet clear. The accumulation of acidotropic molecules could itself be affected by other factors including the presence of transporters in the limiting membrane and the available aqueous volume, which will alter as a result of content condensation occurring during lysosome re-formation [9, 36]. Johnson et al. [35] investigated why some lysosomes are more alkaline than others by measuring buffering capacity as well as using assays of passive H^+^ permeability and lysosome reacidification, in the presence and absence of a V-ATPase inhibitor, after lysosomal transmembrane pH gradient dissipation with a protonophore. These assays provided evidence that increased passive (leak) permeability to protons together with reduced V-ATPase activity accounts for the reduced acidifying ability of some lysosomes. In this context, it is interesting to note the removal of V-ATPase to generate a neutral post-lysosomal compartment in *Dictyostelium discoideum* [37], the reversible dissociation/assembly of the V-ATPase V0V1 complex to regulate ATP-dependent proton transport [38] and the evidence that modulation of the activity of the Rab7 effector RILP (Rab-interacting lysosomal protein) may be used to control V-ATPase function [39].

The re-formation of terminal lysosomes from endolysosomes is not well understood, although previous studies of lysosome re-formation in cultured cells and in cell-free experiments have suggested that it requires budding and tubulation events at the surface of the endolysosome and is a maturation process that includes content condensation [9, 36, 40, 41]. Interestingly, re-formation of lysosomes from autolysosomes, which result from autophagosome-lysosome fusion [42], has been previously shown to occur via lysosomal membrane glycoprotein-positive tubules [43, 44, 45, 46, 47], just as we observed when studying re-formation of lysosomes after invertase uptake into cells containing sucrosomes (swollen endolysosomes). We did not extend our sucrosome/invertase experiments to the other cell lines that we studied because we observed that generating sucrosomes in HeLa and MCF7 cells, while possible (see also [48] for HeLa cells), was not as straightforward as in NRK cells. For these cells types, it may be preferable to use a different non-hydrolysable disaccharide or other indigestible substance [49, 50]. Consistent with our data on lysosome re-formation from sucrosomes, a recent study of tubulation of late endocytic compartments in macrophages showed that tubules forming from a parent endolysosomal compartment, and considered to be nascent lysosomes, contained the same luminal fluorescent marker as the parent compartment [40]. The fact that lysosome re-formation from endolysosomes is a maturation process implies that the endolysosomal compartment will be heterogeneous since each organelle will be at a different stage in the formation/re-formation cycle. This is consistent with the heterogeneous ultrastructural morphology seen in the present study and with the wide range of pH in individual endolysosomes observed by DAMP labeling or ratiometric imaging. It also means that simply investigating whether a marker such as MPR or a specific Rab is present may not be a sufficient means of determining intermediate stages in the cycle. At present we also do not know the fusogenic capability of tubules breaking off from endolysosomes or how this alters during the maturation process leading to lysosome re-formation.

The availability of a pool of inactive acid hydrolases in terminal storage lysosomes could allow the cell to respond to altered extracellular conditions or cellular stress by increasing the rate at which terminal lysosomes fuse with endosomes, autophagosomes or phagosomes and thus increase degradative activity. Maintaining part of the cell’s complement of acid hydrolases in an environment where they are inactive may also increase the protection of both limiting membrane and luminal contents, thereby enhancing lysosome longevity. At present, we do not know whether there are nutritional or signaling conditions under which the ratio of acid-hydrolase-active endolysosomes to inactive lysosomes would alter, nor do we fully understand the relationship of endolysosomes to autolysosomes, although the two may be synonymous depending on nutritional state. These are likely to be fruitful areas of further study, not least because of the increasingly recognized importance of the cytoplasmic surface of the limiting membranes of endolysosomes/lysosomes as the site of action of signaling complexes that regulate cellular metabolism (reviewed in [3]).

## Experimental Procedures

Further details on experimental procedures (e.g., reagents, antibodies, plasmids, and microscopy) are provided in the Supplemental Experimental Procedures.

### Endocytic Uptake of Fluorescent Dextrans and DQ-BSA

Terminal endocytic compartments in cultured NRK fibroblasts, HeLa-M epithelial cells, or MCF7 human breast adenocarcinoma cells were loaded with 0.5 mg/ml lysine-fixable dextran-OG 488, dextran-Texas red, or dextran-Alexa 647, all from Thermo Fisher Scientific in culture medium for 4 hr at 37°C followed by incubation in conjugate-free medium for 20 hr as previously described [9]. For labeling of proteolytically active endocytic compartments, cells were incubated with 0.1 mg/ml DQ Green BSA from Thermo Fisher Scientific for 15–30 min at 37°C in culture medium.

### Incubation with Cathepsin Substrates

To label endocytic organelles in which cathepsin B or cathepsin L was catalytically active, cells were incubated with the respective Magic Red substrates from ImmunoChemistry Technologies, at a final concentration of 673 nM. Glycyl-L-phenylalanine 2-naphthylamide (GPN) from Santa Cruz Biotechnology was used at a final concentration of 200 μM.

### Accumulation of Acidotropic Compounds

For labeling of acidic organelles for fluorescence microscopy, cells were incubated for 5 min at 37°C with 50 nM LysoTracker Green from Thermo Fisher Scientific. For TEM, cells were incubated for 30 min at 37°C with 30 μM DAMP, before aldehyde fixation, processing for TEM and immunolabeling with anti-dinitrophenol (DNP) antibodies.

### Microscopy

Preparation, fixation, and labeling of cells for microscopy, acid phosphatase cytochemistry, preparation of sections for TEM, CLEM, immuno-EM, use of microscopes, image analysis, and quantification were carried out as described in the Supplemental Experimental Procedures. Confocal and live-cell microscopy was carried out on Zeiss LSM710, 780, or 880 confocal microscopes. EM sections were examined with an FEI Tecnai G2 Spirit BioTwin transmission electron microscope. For quantification of fluorescence microscopy, Pearson’s correlation coefficients and Manders’ colocalization coefficients were used, respectively, to measure the overall association of two fluorescent probes and the fraction of the total fluorescence of one probe overlapping with a second as described in the Supplemental Experimental Procedures. In the figure legends, Manders’ coefficients are presented such that M1, DexOG:MR, indicates the fraction of DexOG-positive pixels containing cresyl violet after hydrolysis of a MR substrate and M2, MR:DexOG, indicates the fraction of MR-positive pixels containing DexOG, likewise for other Manders’ coefficients presented. Quantified data are presented as mean ± SEM (number of experiments with ≥17 cells per experiment, unless otherwise stated). Quantitation of LC3 fluorescence intensity to assess autophagy is presented as mean ± SEM (number of experiments with >100 cells per condition in each experiment). Nuclear translocation of TFEB-GFP was assessed with a Cellomics ArrayScan VTi high content screening microscope (Thermo Fisher Scientific), and data are presented as mean ± SEM (number of experiments with 750 cells per condition in each experiment).

## Author Contributions

N.A.B. and J.P.L. conceived and directed the study. N.A.B. and L.J.D. performed all the confocal microscopy experiments including quantification, with N.A.B. additionally undertaking all the EM studies and L.J.D. preparing and studying cells stably expressing TFEB-GFP. All the authors discussed the results, interpreted data, and co-wrote the paper.

## Conflicts of Interest

L.J.D. is supported by a BBSRC industrial CASE studentship with GSK Research and Development.

## Supplemental Information

Document S1. Supplemental Experimental Procedures and Figures S1–S6

Movie S1. Magic Red-Positive Endolysosomes Contain Active Cathepsin C Terminal endocytic compartments of NRK cells were loaded with DexOG for 4 h followed by a 20 h chase in DexOG-free medium. Cathepsin-active endolysosomes were revealed by incubation with cathepsin B Magic Red substrate for 2 min and time-lapse images of the living cell collected on the confocal microscope. Addition of 200 μM GPN resulted in rapid dissipation of cresyl violet from the cathepsin-active endolysosomes but the DexOG (Mr 10,000) was retained in these organelles. Upper panels: false color; lower panels: grayscale.

Movie S2. Lucifer Yellow Is Dissipated from a Subpopulation of Organelles by Incubation with GPN Terminal endocytic compartments of NRK cells were loaded with LucY for 4 h followed by a 20 h chase in LucY -free medium and the cells then incubated for 2 min with cathepsin B Magic Red substrate. Time-lapse images of the living cells were collected on the confocal microscope. Addition of 200 μM GPN resulted in rapid dissipation of cresyl violet and LucY from the cahepsin-active endolysosomes but LucY was retained in a population of the terminal endocytic organelles revealing that it also resided in organelles that were not catalytically active for cathepsin C. Scale Bar: 10 μm. Upper panels: false color; lower panels: grayscale.

Movie S3. Cathepsin B Catalytic Activity Recovers after GPN Washout Terminal endocytic compartments of NRK cells were loaded with DexOG for 4 h followed by a 20 h chase in DexOG-free medium. Cathepsin-active endolysosomes were revealed by incubation with cathepsin B Magic Red substrate for 2 min and images of the living cells collected on the confocal microscope. Addition of 200 μM GPN resulted in rapid dissipation of cresyl violet from the cathepsin-active endolysosomes but the DexOG was retained. Washout of the GPN with medium containing cathepsin B Magic Red substrate showed that cathepsin B was retained and remained catalytically active upon removal of GPN. Upper panels: false color; lower panels: grayscale.

Movie S4. Organelle Kissing Causes Cathepsin Activation in the Newly Forming Endolysosome Terminal endocytic compartments of NRK cells were pre-loaded with DexA for 4 h followed by a 20 h chase in DexA-free medium and then late endosomes were loaded with DexOG by uptake for 10 min followed by a 5 min chase in DexOG-free medium containing cathepsin B Magic Red substrate. Time-lapse confocal microscopy of the living cell revealed that transient fusions of a DexOG-positive late endosome with DexA-laden terminal endocytic compartments resulted in the gradual acquisition of DexA (blue) in the DexOG-positive organelle (green) and a subsequent rise in cresyl violet fluorescence from the cleaved Magic Red substrate (red).

Movie S5. Emergence of Tubules after Formation of a Cathepsin-Active Endolysosome Terminal endocytic compartments of NRK cells were loaded with DexA for 4 h followed by a 20 h chase in DexA-free medium and late endosomes were loaded with DexOG by uptake for 10 min followed by a 5 min chase in DexOG-free medium containing cathepsin B Magic Red substrate. Time-lapse confocal microscopy of a living cell revealed a complete fusion between a DexOG-laden organelle and DexA-laden organelle(s) and subsequent rise in cresyl violet fluorescence from the cleaved cathepsin B Magic Red substrate. Extrusion and detachment of a re-formation tubule (arrowheads) containing each of the 3 fluorochromes was seen after the fusion.

Movie S6. Tubulation of Sucrosomes after Invertase Uptake Sucrosomes were formed in NRK cells by endocytosis of 30 mM sucrose for 24 h and subsequently incubated with medium containing 0.5mg/ml invertase for 1 h followed by the addition of cathepsin B Magic Red substrate for 2 min. Incubation with cathepsin B Magic Red substrate revealed that the swollen sucrosomes were cathepsin-active and a time-lapse series of images collected on the confocal microscope showed that addition of invertase resulted in extensive tubulation of the cathepsin-active sucrosomes and in some instances tubules could be observed detaching from the parent sucrosome (arrowhead).

Document S2. Article plus Supplemental Information
