# Supplementary material for: Endolysosomes Are the Principal Intracellular Sites of Acid Hydrolase Activity
Source: Curr Biol. 2016 Sep 12;26(17):2233–45. doi: 10.1016/j.cub.2016.06.046 (PMC5026700; doi:10.1016/j.cub.2016.06.046)
Supplement: Document S1. Supplemental Experimental Procedures and Figures S1–S6 [file mmc1.pdf]

Current Biology, Volume *26*

Supplemental Information

Endolysosomes Are the Principal Intracellular Sites of Acid Hydrolase Activity

Nicholas A. Bright, Luther J. Davis, and J. Paul Luzio

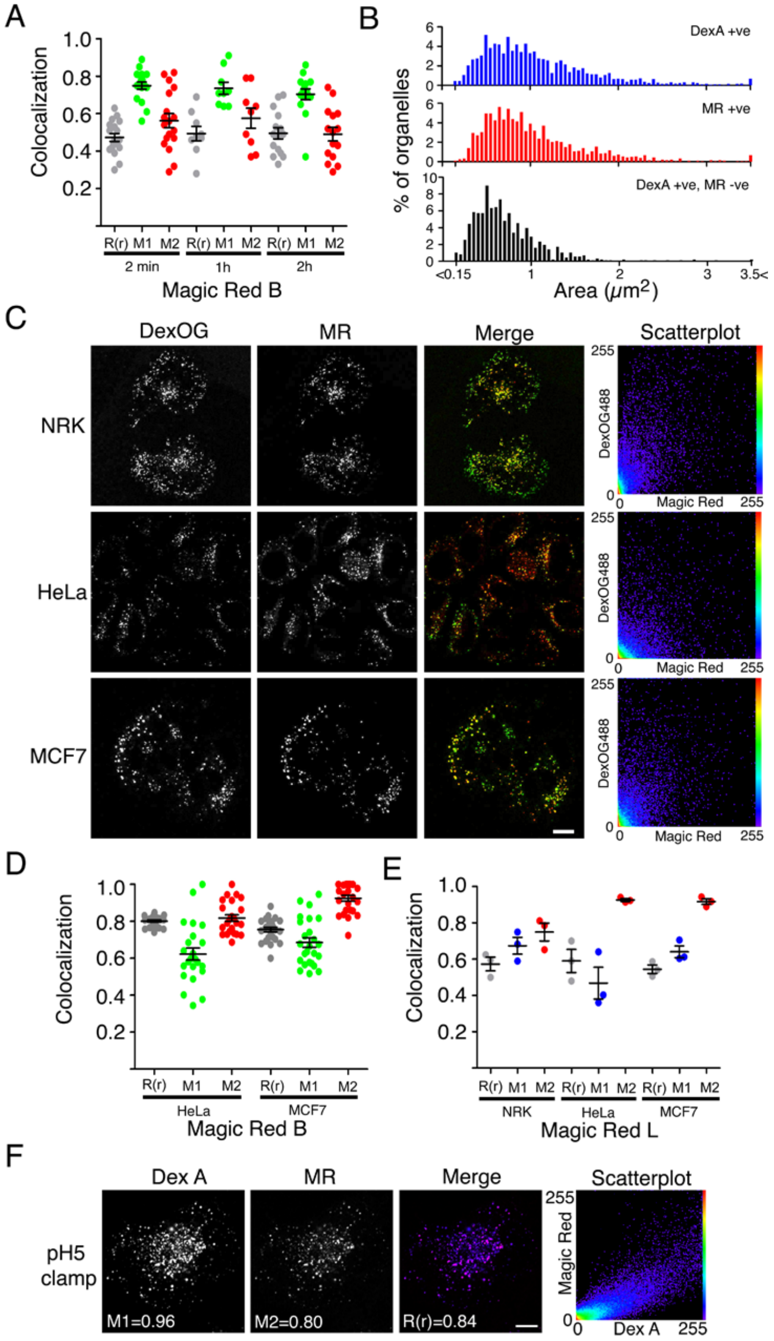

Figure S1: Endolysosomes are the principal sites of cathepsin B and cathepsin L catalytic activity. Related to Figure 1. Terminal endocytic compartments were loaded with fluorescent dextran (DexOG or DexA) as for Figure 1. **A**) Pearson's (R(r); gray) and Mander's (M1, green, DexOG:MR and M2, red, MR:DexOG) correlation coefficients (mean ± SEM within a single experiment), for colocalization of pre-loaded DexOG and hydrolysed (different times) cathepsin B MR substrate in NRK cells (all data from the same experiment shown in Figure 1B). **B)** Size distribution of all DexA-positive; MR-positive; MR-negative, DexA-positive organelles in NRK cells (single experiment). **C**) Cathepsin-active terminal endocytic organelles in NRK, HeLa and MCF7 cells revealed by incubation with 1 h hydrolysed cathepsin B MR substrate. **D**) Pearson's (R(r), gray) and Manders' (M1, green, DexOG:MR and M2, red, MR:DexOG) correlation coefficients for colocalization of pre-loaded DexOG and 1 h hydrolysed cathepsin B MR substrate in HeLa or MCF7 cells (mean ± SEM within a single experiment). **E**) Pearson's (R(r); gray) and Mander's (M1, blue, DexA:MR and M2, red,MR:DexA) correlation coefficients for colocalization of pre-loaded DexA and 1 h hydrolysed cathepsin L MR substrate in NRK, HeLa and MCF7 cells (mean ± SEM (3)). **F)** Cathepsin B MR substrate access to all terminal endocytic organelles in a NRK cell in which the culture medium was replaced with a pH 5-buffered medium at 37°C containing the cathepsin B MR substrate, 10 μM monensin and 10 μM nigericin. The representative image taken after 5 min on the confocal microscope shows the coincidence of DexA-positive and MR-positive organelles (Pearson's R(r) and Manders' M1, DexA:MR and M2, MR:DexA values given for cells shown), and a scatterplot of the merged image reveals the coincidence of the two fluorochromes. Scale Bars: C) 10 μm, F) 5 μm.

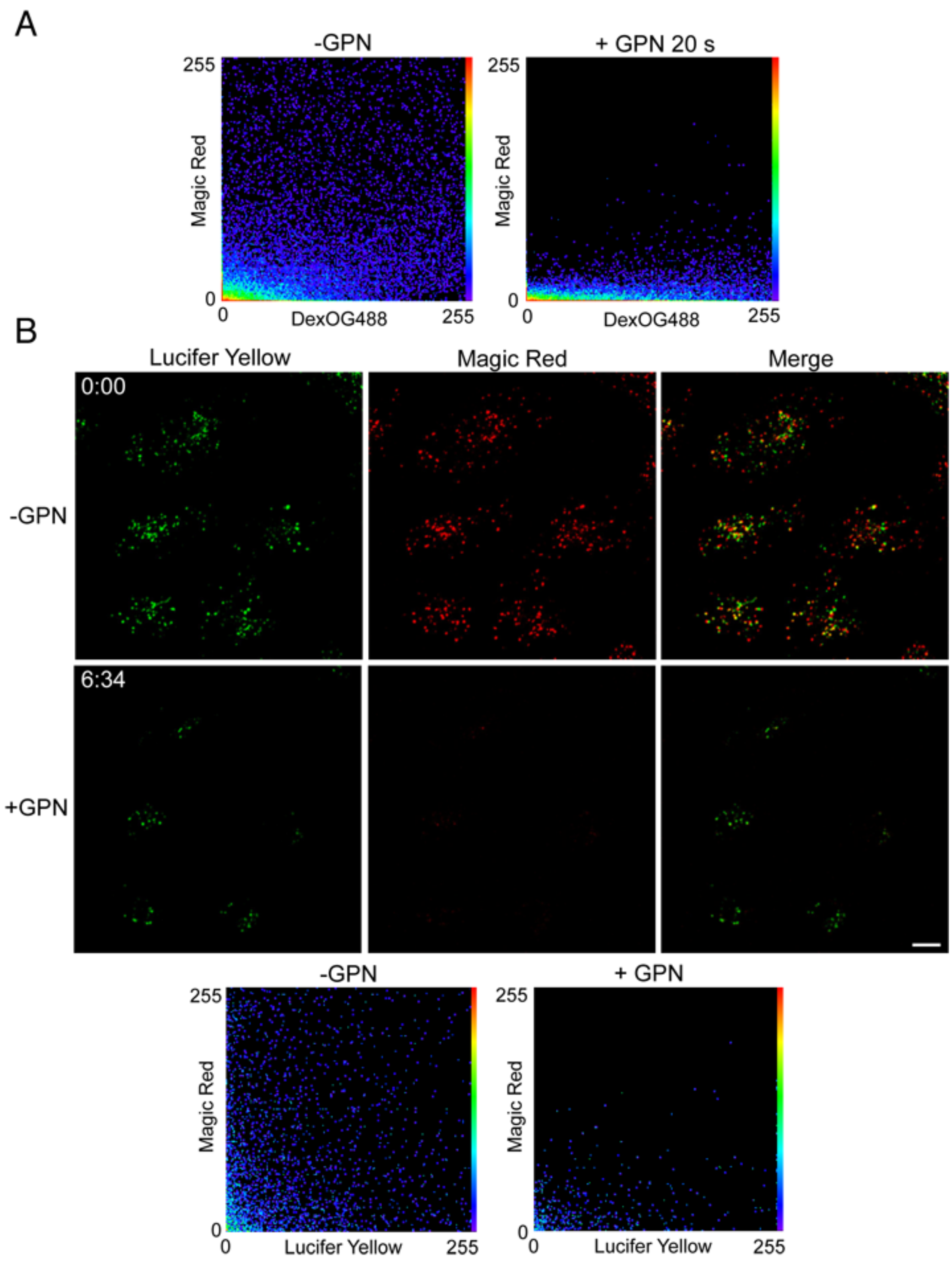

Figure S2: Magic Red-positive endolysosomes contain active cathepsin C. Related to Figure 2.
A) Scatterplots of the pixel intensities of the merged images shown in Figure 2A, taken from a timelapse confocal microscopy series before, or 20 s after addition of 200 μM GPN, revealing rapid dissipation of cresyl violet (Magic Red). **B)** Terminal endocytic compartments of NRK cells were loaded with Lucifer Yellow for 4 h followed by a 20 h chase in Lucifer Yellow-free medium and the cells then incubated for 2 min with cathepsin B MR substrate. Timelapse images of the living cells were collected on the confocal microscope. Addition of 200 μM GPN resulted in rapid dissipation of cresyl violet (Magic Red) from cathepsin-active endolysosomes but the Lucifer Yellow (Mr 457) was retained in a population of the terminal endocytic organelles revealing that it also resided in organelles that were not catalytically active for cathepsin C. Scatterplots of the pixel intensities of the images taken before and after the addition of GPN clearly show the dissipation of cresyl violet (Magic Red) from the cells but the retention of Lucifer Yellow. Scale Bar: 10 μm.

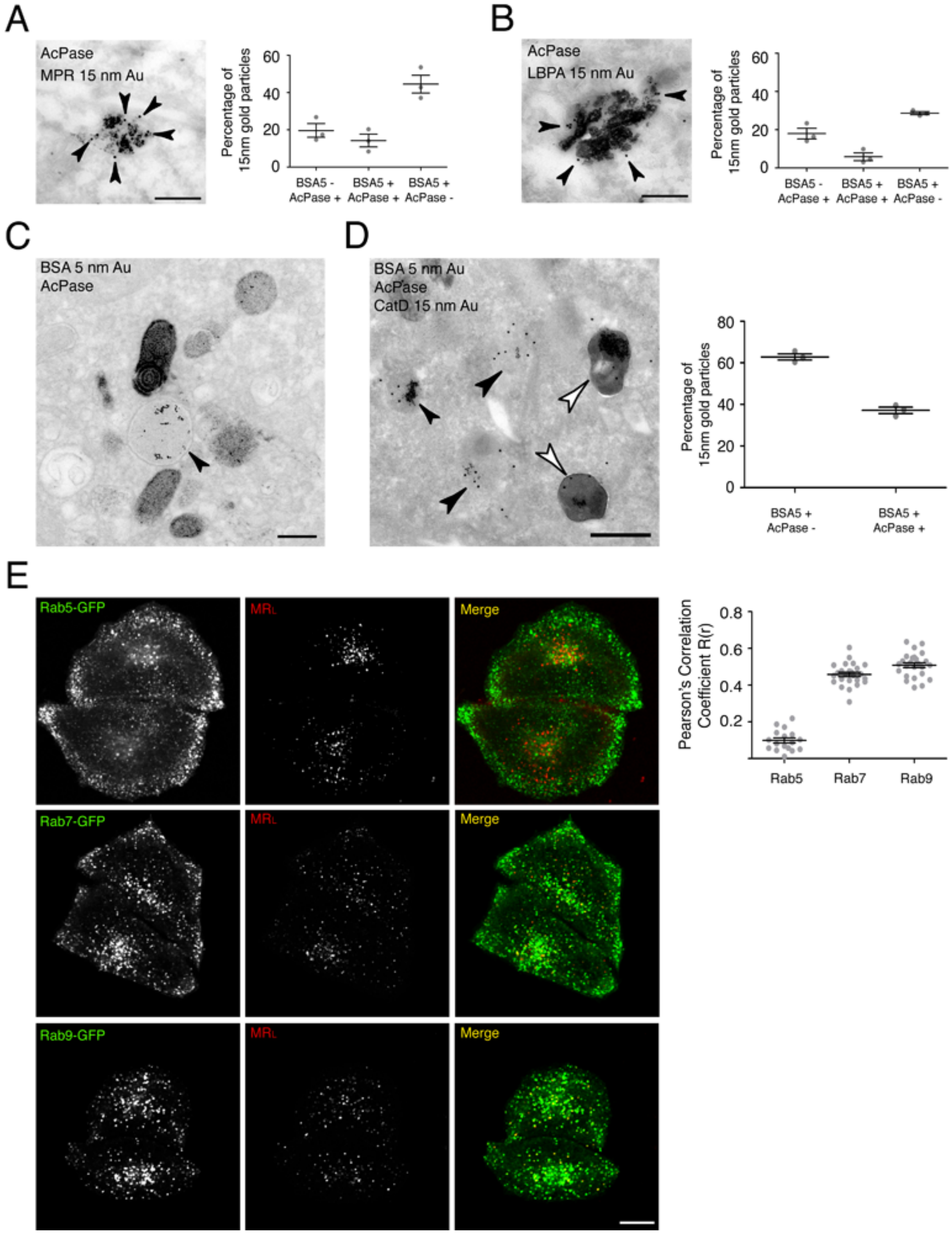

Figure S3: Characterisation of endolysosomes. Related to Figure 3. Terminal endocytic compartments of NRK (A-C) and MCF7 (D) cells were loaded with BSA-gold as for Figure 3. **A-B)** Immuno-EM of NRK cells in which the presence of active AcPase was revealed by cytochemistry, with cytidine 5'-monophosphate and lead nitrate capture for 1 h, showed that the AcPase-active organelles labelled with lead phosphate deposits were also immunolabelled with **A**) anti-MPR (15 nm colloidal gold, arrowheads) and **B**) anti-LBPA (15 nm colloidal gold, arrowheads). Graphs show distribution of label (in 3 experiments 1729 gold particles counted for MPR and 1860 for LBPA). **C)** TEM of NRK cells in which the presence of active AcPase was revealed by cytochemistry using β-glycerophosphate and cerium chloride capture, revealed the presence of 5 nm gold-positive organelles that were AcPase-negative (black arrowhead). **D)** Immuno-EM of MCF7 cells (active AcPase was revealed by cytochemistry as in panel C), showed that both AcPase-active (white arrowheads) and inactive (black arrowheads) organelles were immunolabelled with anti-cathepsin D (15 nm colloidal gold). Graph shows the distribution of label (in 3 experiments 1592 gold particles counted). **E)** Confocal immunofluorescence microscopy images and colocalization analysis (graph shows Pearson's (R(r) correlation coefficients) of HeLa cells stably expressing either GFP-labelled Rab5, Rab7 or Rab9 after 2 min incubation with cathepsin L MR substrate (MR_L). Scale Bars: A-C) 200 nm, D) 500 nm, E) 10 µm.

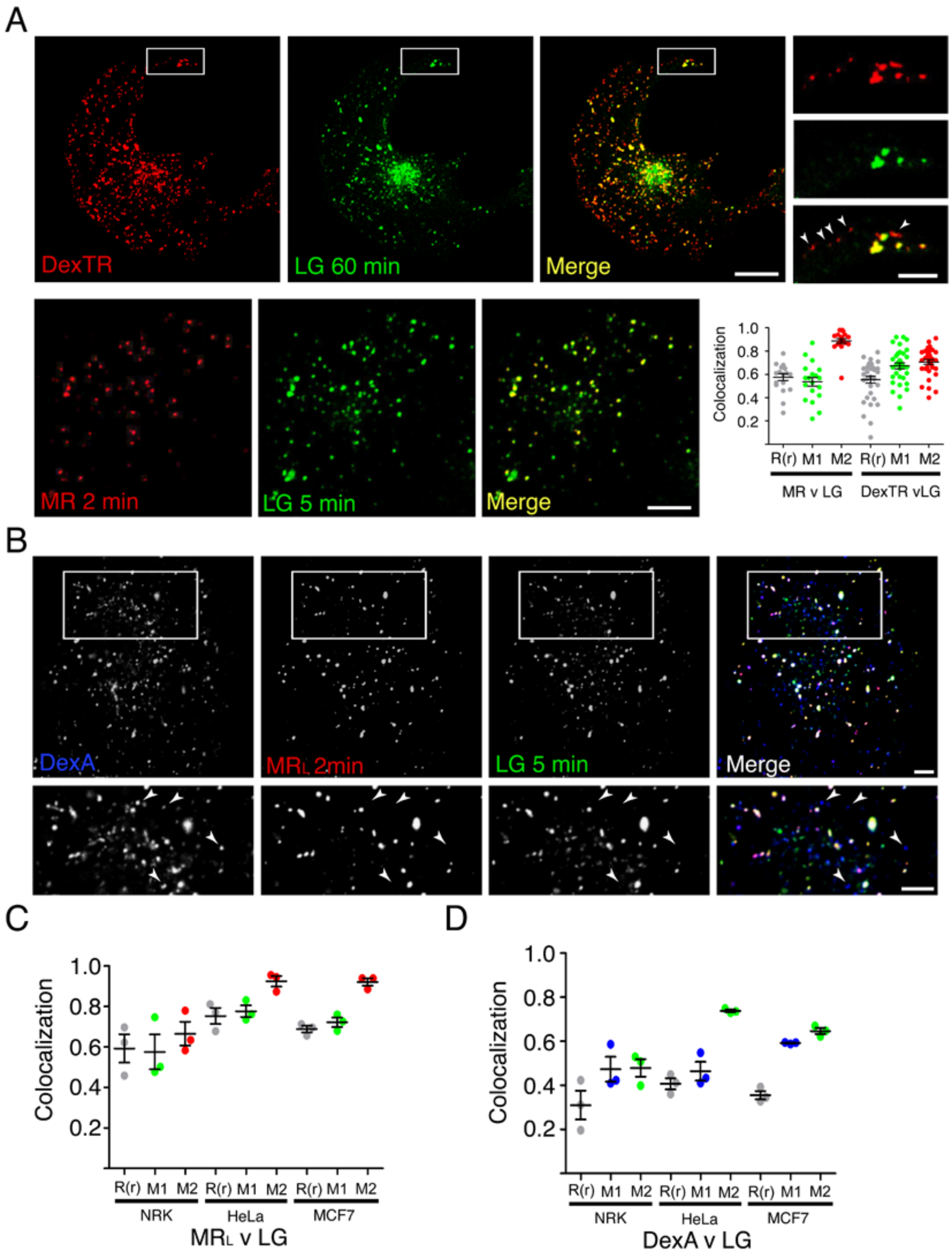

Figure S4: Cathepsin-active endolysosomes accumulate acidotropic molecules but terminal lysosomes do not. Related to Figure 4. A) Confocal fluorescence microscopy of NRK cells, pre-loaded with DexTR, to label terminal endocytic compartments, and incubated for 1 h with 50 nM LysoTracker™ Green (LG; upper panel) or with Magic Red B substrate (MR) for 2 min followed by 50 nM LysoTracker™ Green (LG) for 5 min (lower panel). An enlargement of the boxed region in the cell shown in the upper panel shows DexTR-loaded terminal endocytic compartments that did not accumulate the acidotropic molecule (arrowheads). Pearson's (R(r); gray) and Manders' (M1 and M2) correlation coefficients for colocalization of LG and MR or DexTR are shown (mean ± SEM of ≥ 17 cells). M1(left), green, LG:MR; M2 (left), red, MR:LG; M1 (right), green, LG:DexTR; M2 (right), red, DexTR:LG. **B)** Enlarged confocal immunofluorescence microscopy images of NRK cells pre-loaded with DexA, to label terminal endocytic compartments, and incubated for 2 min with the cathepsin L MR substrate (MR_L) followed by addition of 50 nM LysoTracker™ Green (LG) for 5 min. Arrowheads show DexA-positive terminal endocytic organelles that are both MR_L-negative and LG-negative (correlation coefficients for colocalization are shown in Figure S1E). **C)** Pearson's (R(r); gray) and Manders' (M1, green, LG:MR and M2, red, MR:LG)) correlation coefficients for colocalization of MR_L and LG in NRK, HeLa or MCF7 cells (mean ± SEM, 3 separate experiments). **D)** Pearson's (R(r); gray) and Mander's (M1, blue, DexA:LG and M2, green, LG:DexA) correlation coefficients for colocalization of LG and DexA in NRK, HeLa or MCF7 cells (mean ± SEM (3)). Scale Bars: A) 20 µm (enlargement 5 µm), B) 5 µm.

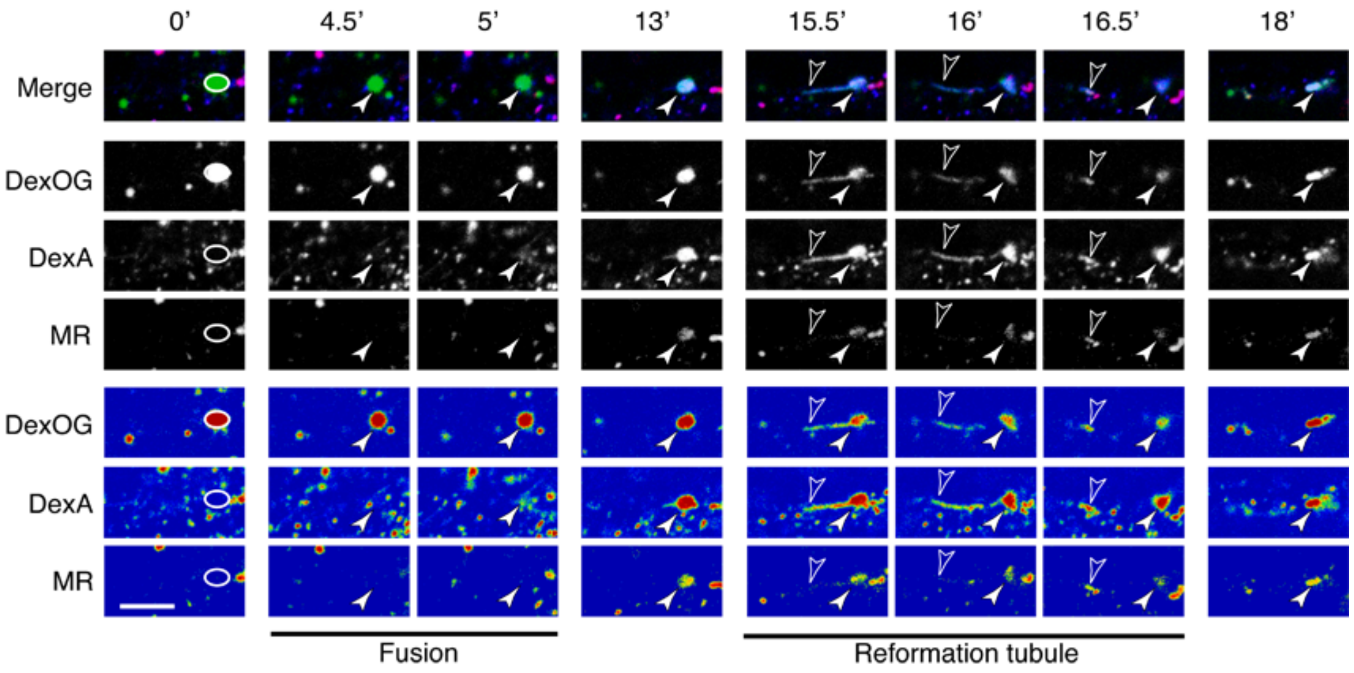

Figure S5: Emergence of tubules after formation of a cathepsin-active endolysosome. Related to Figure 5. Terminal endocytic compartments of NRK cells were pre-loaded with DexA and late endosomes loaded with DexOG and chased in medium containing cathepsin B MR substrate as for Figure 5. Timelapse confocal microscopy of a living cell showing a complete fusion between a DexOG-laden organelle and DexA-laden organelle(s) and subsequent rise in cresyl violet fluorescence from the cleaved cathepsin B MR substrate. The DexOG labelled late endocytic organelle is circled in the first panel and indicated by the solid arrowheads in subsequent panels. The false-colour images of the data (bottom 3 rows) depict background intensity (blue), low (green), intermediate (yellow) and high (red) fluorescence intensity of each of the fluorochromes. Extrusion and detachment of a reformation tubule (open arrowheads) containing each of the 3 fluorochromes was seen after 15.5 min. Scale Bar: 5 μm.

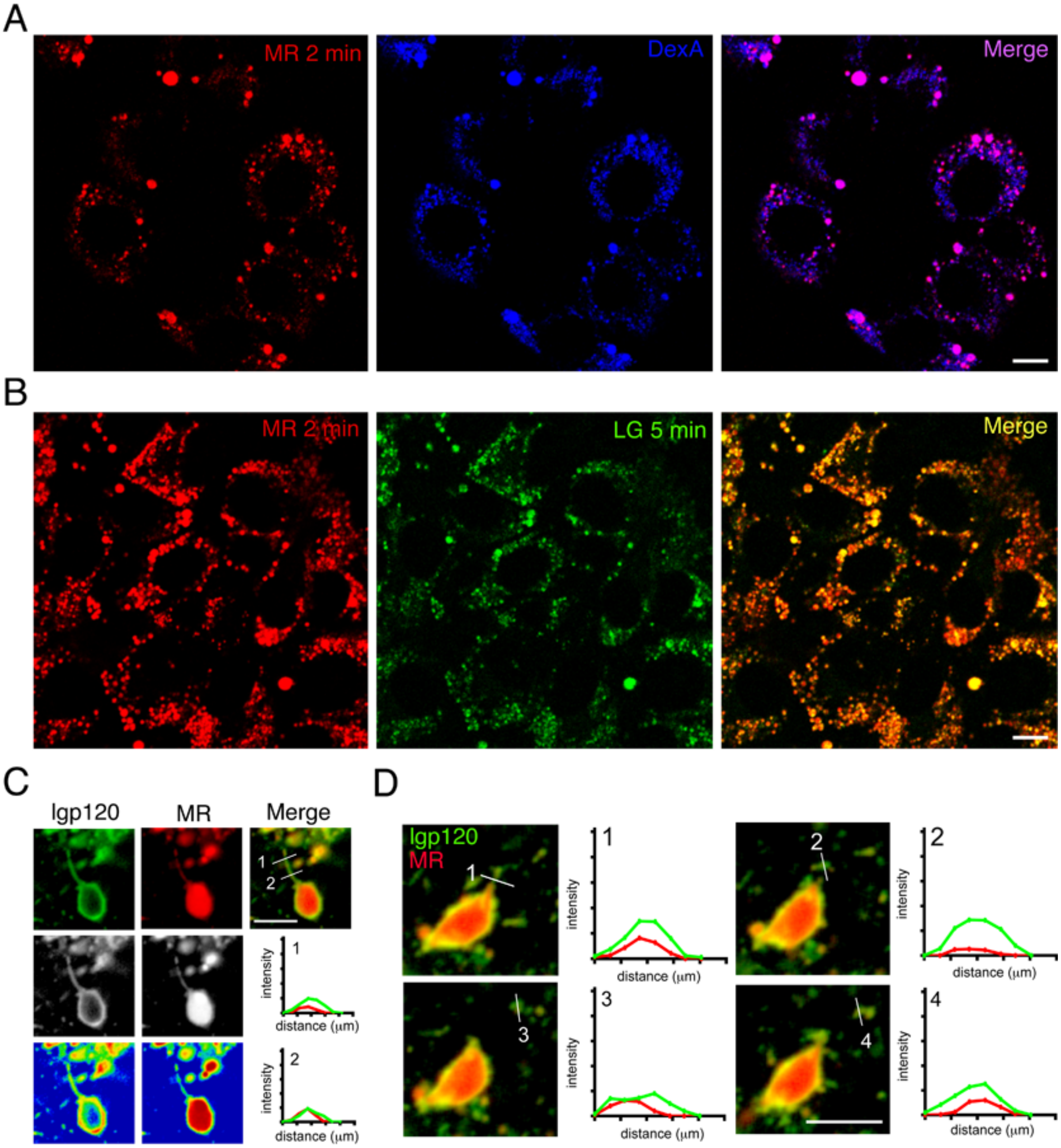

Figure S6: Sucrosomes and the re-formation of lysosomes. Related to Figure 6.
A and B. Terminal endocytic compartments of NRK cells were loaded with DexA, sucrosomes formed and cells incubated with cathepsin L MR substrate (MR_L) and LysoTracker^{TM} Green (LG) as for Figure 6. Sucrosomes (swollen vacuoles) contained DexA, were cathepsin L –active (A) and acidic (B). **C)** Sucrosomes were formed in NRK cells stably expressing lgp120-GFP as for Figure 6 and the cells subsequently incubated for 1 h in medium containing 0.5 mg/ml invertase followed by cathepsin B MR substrate for 2 min. A representative image, and pixel intensity line-scans, taken from a timelapse series demonstrate a reformation tubule containing the limiting membrane marker lgp120-GFP (green) and the cresyl violet lumenal content marker (red, MR). The false-colour images of the data (bottom panels) depict background intensity (blue), low (green), intermediate (yellow) and high (red) fluorescence intensity of each of the fluorochromes. **D)** Four sequential images and pixel intensity line-scans taken from a timelapse series of NRK cells stably expressing lgp120-GFP treated as in (C) show that the reformation tubule observed contains the limiting membrane marker lgp120-GFP (green) and the cresyl violet lumenal content marker (red, MR), both during extrusion and subsequent detachment from the parent sucrosome. Scale Bars: A-B) 10 µm, C-D) 5 µm.

Supplemental Experimental Procedures

Reagents

Dulbecco's modified Eagle's medium (DMEM), Roswell Park Memorial Institute medium (RPMI 1640), fetal calf serum (FCS), cytidine 5'-monophosphate di-sodium salt, cerium(III) chloride heptahydrate, beta-glycerophosphate di-sodium salt hydrate, Lucifer Yellow di-lithium salt, chloroquine and invertase were from Sigma-Aldrich (Poole, UK). Magic Red™ cathepsin B and L substrates were from ImmunoChemistry Technologies (Bloomington, MN USA). Glycyl-L-phenylalanine 2-naphthylamide (GPN) was from Santa Cruz Biotechnology (Dallas, USA). Nigericin (sodium salt) and monensin (sodium salt) were from the Cayman Chemical Company (Ann Arbor, MI, USA). *N*-(3-((2,4-dinitrophenyl)amino)propyl)-*N*-(3-aminopropyl)methylamine, dihydrochloride (DAMP) was from ThermoFisher Scientific (Paisley, UK). Dextran-Oregon Green 488 (DexOG; M_r 10,000, anionic, fixable), dextran-Texas Red™ (DexTR; M_r 10,000, anionic, fixable), dextran-Alexa 647 (DexA; M_r 10,000, anionic, fixable), LysoTracker™ Green, DQ™ Green BSA, rabbit anti-dinitrophenol (DNP), rabbit anti-Texas Red™, donkey anti-mouse: Alexa 594, donkey anti-rabbit: Alexa 594, donkey anti-mouse: Alexa 488, donkey anti-mouse: Alexa 647, Hoechst 33342, Geneticin(G418) and CO_2-independent medium were from ThermoFisher Scientific (Paisley, UK). Lead nitrate was from Agar Scientific (Stansted, UK). Protein A gold conjugates were from the Department of Cell Biology, University of Utrecht. BSA conjugated to 5 nm colloidal gold (BSA-gold) was prepared as described previously [S1]. The mouse monoclonal anti-cation-independent mannose-6-phosphate receptor antibody (clone 2G11) was from Abcam (Cambridge, UK), the mouse monoclonal anti-LC3 (4E12) from MBL International (Woburn, MA USA) and the rabbit anti-cathepsin D antibody was from EMD Millipore (Billerica, MA USA). The mouse monoclonal anti-LBPA antibody (clone 6C4) was a generous gift from Professor Jean Gruenberg, University of Geneva. The rabbit anti-lgp 110 antibody has been described previously [S2]. Torin 1 was from Tocris Bioscience (Bristol, UK), and bafilomycin A1 from Alfa Aesar (Heysham, UK). The TFEB-GFP construct in a pEGFP vector was a gift from Andrea Ballabio (Telethon Institute of Genetics and Medicine, Pozzuoli, NA, Italy) and was subcloned into a pLXIN retroviral expression vector (Clontech, Mountain View, CA, USA). The lgp120-GFP in pEGFP N1, with a 10 amino acid linker between the lgp120 cytosolic tail and the eGFP is available from Addgene (Cambridge, MA, USA) and was subcloned into pLXIN. HeLa cells stably expressing EGFP-tagged Rab5, Rab 7 and Rab9 [S3] were a gift from Dr Matthew Seaman (Cambridge Institute for Medical Research, UK).

Cell Culture and Transduction

NRK fibroblasts and human breast adenocarcinoma MCF7 cells were cultured in DMEM and HeLa-M epithelial cells in RPMI 1640, both media supplemented with 10% (v/v) FCS, 100 IU/ml penicillin, 100 μg/ml streptomycin, 4.5 g/l glucose, and 2 mM L-glutamine. NRK cells stably expressing TFEB-GFP or lgp120-GFP were generated using the pLXIN retroviral system as previously described [S4]. Transduced cells of mixed expression levels were selected by culture in medium supplemented with 0.5 mg/ml Geneticin (G418) and a clonal NRK cell line expressing TFEB-GFP was subsequently generated after isolating single cells by fluorescence activated cell sorting.

Cells were seeded onto 15 mm glass coverslips for fixed-cell confocal microscopy, 42 mm diameter glass coverslips (PeCon, GmbH, Germany) for live cell imaging, gridded glass bottom dishes (MatTek Ashland, MA, USA) for CLEM, 25 cm^2 tissue culture flasks for immuno-EM, glass-bottom 96-well plates for Cellomics ArrayScan™ wide-field microscopy, Deckgläser 25 mm coverslips (Techmate Ltd, Milton Keynes, UK) for ratiometric imaging and grown in a 5% CO_2 incubator at 37°C.

Incubation with cathepsin substrates and acidotropic compounds

To label endocytic organelles in which cathepsin B or cathepsin L was catalytically active, cells were incubated respectively with cathepsin B MR (Magic Red™) substrate or cathepsin L MR substrate, prepared in CO_2-independent medium / 10 % FCS, but used at a 10-fold dilution (final concentration 673 nM) compared to the manufacturers recommendations. The substrate was added directly to cells growing at 37°C on PeCon coverslips or MatTek glass bottom dishes on the incubated stage of a Zeiss confocal microscope (Carl Zeiss Ltd, Welwyn Garden City, UK) and the live cells were imaged to assay the liberation of cresyl violet fluorescence. Alternatively, cells were incubated, in a 5% CO_2 incubator, with Magic Red™ cathepsin substrates, prepared in DMEM / 10% FCS, for 2-60 min prior to imaging. GPN was prepared as a 200 mM stock in dimethyl sulphoxide and used at a final concentration of 200 μM.

LysoTracker™ Green was prepared in CO_2-independent medium / 10 % FCS and added to cells on PeCon coverslips or MatTek glass bottom dishes at a final concentration of 50 nM for 5 min at 37°C. Alternatively, cells were incubated in 5% CO_2 incubator in the presence of 50 nM LysoTracker™ Green prepared in DMEM / 10% FCS for 60 min prior to imaging. DAMP was made up as a 100 mM stock solution in H_2O and stored at -20°C before application to cells at 30 μM in DMEM for 30 min at 37°C.

pH clamping

Nigericin and monensin were reconstituted in ethanol as 10 mM stock solutions and stored at -20°C. pH clamping was by modification of a previously described method [S5]. A pH 5 clamping solution was prepared using 25 mM sodium acetate buffer, pH 5 containing 5 mM NaCl, 1 mM $CaCl_2$, 115 mM KCl, 1.2 mM $MgSO_4$, 10 mM glucose, 10 μM nigericin and 10 μM monensin. For fluorescence confocal microscopy, NRK cells in which DexA had been endocytosed for 4 h followed by a 24 h chase were incubated with the pH 5 clamping solution containing 637 nM cathepsin B MR substrate for 5 min.

Fluorescence microscopy

For immunofluorescence microscopy cells were fixed in 4% (w/v) paraformaldehyde in phosphate buffered saline pH 7.4 (PBS) for 10 min and permeabilized with 0.05% (w/v) saponin for 5 min at 20°C. For cytosolic washout cells were rinsed with PBS and incubated with PBS containing 0.05% (w/v) saponin for 30 s at 20°C before fixing with 4% (w/v) paraformaldehyde. All antibody incubations were carried out in PBS with 0.1% (w/v) BSA and 5% (v/v) FCS. Secondary antibodies were Alexa Fluor conjugates used at a final concentration of 4 μg/ml. Images of immunolabelled cells were collected with a x63 1.4 NA Plan Apochromat oil-immersion lens on Zeiss LSM 710, 780 or 880 confocal microscopes. Nuclear translocation of TFEB-GFP in NRK cells stably expressing TFEB-GFP was quantified after fixing with 4% (w/v) paraformaldehyde and staining nuclei with Hoechst 33342 using a Cellomics ArrayScan^{TM} VTi high content screening, wide-field microscope. Images of nuclear translocation were obtained using a Zeiss LSM 780 confocal microscope.

Rapid immunofluorescence protocol for cells incubated with cathepsin MR substrates

A rapid immunolabelling protocol was used to detect antigens in cells in which cresyl violet had been liberated from cathepsin MR substrate, before it dissipated from labelled organelles. Cells were grown on Mattek dishes, incubated with DMEM containing cathepsin MR substrate as described above and fixed with 4% PFA (w/v) in PBS for 5 min at 37°C. Cells were then permeabilised with 0.05% saponin in PBS for 5 min at room temperature, washed with PBS and incubated with 100 μl primary antibody diluted in PBS containing 5% (v/v) FCS and 0.1% (w/v) BSA for 10 min at room temperature. The cells were then washed and incubated with secondary fluorochrome-conjugated antibodies diluted in PBS containing 5% (v/v) FCS and 0.1% (w/v) BSA for 10 min at room temperature. The cells were washed and viewed immediately on the confocal microscope. Using this protocol sufficient cresyl violet was retained to demonstrate positive colocalizations but not for robust quantitation since the cresyl violet had begun to dissipate.

Ratiometric imaging

Dual-fluorochrome ratiometric imaging was performed utilising Oregon Green 488 as the pH-sensitive fluorochrome and Alexa 647 as the pH-insensitive fluorochrome. NRK cells cultured on Deckgläser 25 mm coverslips were incubated in DMEM containing 0.5 mg/ml DexOG and 0.5 mg/ml DexA for 4 h followed by a 20 h chase in conjugate-free DMEM to load terminal endocytic compartments as described above. The coverslips were placed in an Attofluor® Cell Chamber (ThermoFisher Scientific, Paisley, UK), incubated with Magic Red^{TM} cathepsin B substrate as described above and placed on the incubated stage of a Zeiss LSM 780 confocal microscope. To construct a pH calibration curve, cells were incubated with 25 mM sodium acetate buffer, pH 4 or pH 5, 25 mM MES buffer, pH 6 and 25 mM Hepes buffer, pH 7 containing 5 mM NaCl, 1 mM $CaCl_2$, 115 mM KCl, 1.2 mM $MgSO_4$, 10 mM glucose, 10 μM nigericin and 10 μM monensin for 5 min. Images were collected as described above and the mean background-corrected A647/OG488 fluorescence ratio was calculated at each pH. We recorded 12 images and selected a total of 300 regions of interest using NIH Image J (v1.48r) to quantify individual terminal endocytic organelles using the Alexa 647 channel. The background-corrected A647/OG488 ratio of each organelle was used to calculate the pH by interpolation against the calibration curve and plotted against the mean fluorescence intensity of cresyl violet liberated from cleavage of Magic Red^{TM} cathepsin B in each organelle.

Live Cell Imaging

Cells were seeded onto 42 mm diameter glass coverslips (PeCon, GmbH, Germany), inserted into a POC cell chamber and incubated at 37°C. They were then transferred to the incubated stage of an LSM710, 780 or 880 confocal microscope (Carl Zeiss, Ltd., Welwyn Garden City, UK). Live cells were imaged using a x63 1.4 NA Plan Apochromat oil-immersion lens using an Argon laser line at 488 nm (used to excite Oregon Green 488 or EGFP), a HeNe laser line at 543 nm (used to excite Texas Red^{TM} or cresyl violet) and a HeNe laser line at 633 nm (used to excite Alexa 647). Band pass (505–530 nm) and long pass (560 nm) filters were used to separate emission wavelengths of Oregon Green 488 and Texas Red^{TM} respectively. Laser power was attenuated to 2% of maximum to minimize photobleaching and phototoxicity. The detector pinholes were set to give a 0.7-1.4 μm optical slice. Pixel dwell times varied from 1μs to 1.58μs using multitracking (line switching) with a line average of 4. Acquisition was performed using Zen software (Zen 2012 black edition v 8.1) and processing was completed using Adobe Photoshop CS4 (version 11). Time-lapse images of the living cells were recorded every 2-30s and movies were assembled using Adobe Illustrator CS4 (version 14) and NIH Image J (v1.48r).

Acid Phosphatase Cytochemistry

NRK cells were grown in MatTek glass bottom dishes or 25 cm^2 tissue culture flasks and were first incubated with BSA-gold for 4 h followed by a 20 h chase in conjugate-free medium to label terminal endocytic compartments. For TEM of resin-embedded material, cells grown on MatTek glass bottom dishes were fixed by addition of 2% (wt/vol) paraformaldehyde / 2.5% (vol/vol) glutaraldehyde in 0.1 M sodium cacodylate, pH 7.2, to an equal volume of tissue culture medium at 37°C for 1 h. For TEM of cells prepared for immunogold electron microscopy, cells were fixed by addition of 8% (wt/vol) paraformaldehyde / 0.2% (vol/vol) glutaraldehyde in 0.1 M sodium cacodylate, pH 7.2, to an equal volume of tissue culture medium at 37°C for 1 h. The cells were then washed with 0.1 M cacodylate buffer, pH 7.2.

AcPase cytochemistry was performed according to principles established over 50 years ago [S6-S8] using a modified procedure with cerium chloride as a capture reagent for liberated inorganic phosphate [S9]. Cells were incubated for 2 x 15 min with 0.1 M acetate buffer, pH 5, containing 2 mM cerium chloride at 37°C and then for 2 x 60 min with 0.1 M acetate buffer, pH 5, containing 2 mM cerium chloride and 5 mM sodium β-glycerophosphate. The cells were then washed and processed for TEM as described below. For immuno-EM, cells were either processed as described above with cerium chloride or incubated for 2 x 15 min with 0.1 M acetate buffer, pH 5 containing 3.6 mM lead nitrate at 37°C and then for 2 x 30 min with 0.1 M acetate buffer, pH 5 containing 3.6 mM lead nitrate and 1 mM cytidine 5'-monophosphate. The cells were then washed and processed for immuno-EM as described below.

Correlative Light and Electron Microscopy

After visualization of living cells growing on MatTek gridded, glass bottom dishes by confocal microscopy they were fixed by addition of 4% paraformaldehyde / 5% glutaraldehyde in 0.1 M Na cacodylate buffer (pH 7.2) to an equal volume of culture medium. The position of the cells was recorded and confocal Z-series and DIC images were acquired to enable subsequent identification of the cells by TEM. After incubation for 5 min at 37°C, the solution was replaced with 2% paraformaldehyde / 2.5% glutaraldehyde in 0.1 M Na cacodylate buffer (pH 7.2). Cells were processed for TEM as detailed below, embedded in Agar 100 resin, cured, and the glass coverslip was removed by immersion in liquid nitrogen. Ultrathin sections (50 nm) were cut "en face" to the plane of the coverslip and mounted on formvar/carbon-coated slot EM grids.

TEM

Cells were fixed with 2% (wt/vol) paraformaldehyde / 2.5% (vol/vol) glutaraldehyde in 0.1 M sodium cacodylate buffer, pH 7.2, at 37°C, post fixed with 1% OsO_4 in 0.1 M sodium cacodylate buffer, pH 7.2, 'en bloc' stained with 0.5% (wt/vol) uranyl acetate in 50 mM sodium maleate buffer, pH 5.2 and processed for TEM as previously described [S1]. Cell monolayers were embedded in Agar 100 resin (Agar Scientific, Stansted, United Kingdom) by inverting a resin-filled BEEM capsule over them, polymerised and the coverslip was removed by immersion in liquid nitrogen. Ultrathin sections (50 nm) were cut parallel to the plane of the coverslip and mounted on formvar/carbon-coated slot EM grids. Sections for CLEM were stained with uranyl acetate and lead citrate and sections for AcPase cytochemistry were viewed unstained. Sections were examined with an FEI Tecnai G2 Spirit BioTwin transmission electron microscope (Eindhoven, The Netherlands) at an operating voltage of 80 kV and images were recorded with an Eagle 4K CCD camera.

Immuno-EM

Cells were fixed with 4% (wt/vol) paraformaldehyde / 0.1% (vol/vol) glutaraldehyde in 0.1 M sodium cacodylate buffer (pH 7.2), and pelleted in an Eppendorf tube (11,000 rpm for 5 min). The fixative was aspirated, and the cell pellet was resuspended in warm 12% (wt/vol) gelatin in PBS. The cells were then pelleted (11,000 rpm for 5 min) and the gelatin-enrobed cells were set on ice, trimmed into 1 mm^3 blocks and infused with 1.7 M sucrose / 15% (wt/vol) polyvinylpyrolidone for 24 h at 4°C. The blocks were subsequently mounted on cryostubs and snap-frozen in liquid nitrogen. Frozen ultrathin sections were cut using a diamond knife in an ultramicrotome with a cryochamber attachment (Leica, Milton Keynes UK) at −120°C, collected from the knife-edge with 50:50 2% (wt/vol) methyl cellulose: 2.3 M sucrose [S10] and mounted on formvar-carbon–coated EM grids.

Immunolabeling of the cation-independent mannose 6-phosphate receptor, LBPA, DAMP, cathepsin D and Texas Red^{TM} was performed using the protein A-gold technique at room temperature [S11] as described previously [S1, S12]. The sections were contrasted by embedding in 1.8% (wt/vol) methyl cellulose / 0.3% (wt/vol) uranyl acetate and air-dried before observation in a TEM as described above.

Quantitative analysis of the subcellular distribution of gold particles was as described previously [S1].

Quantitation of DAMP labelled organelles

Quantitation of the labelling density of DAMP-labelled organelles was performed using iTEM software (Olympus Soft Imaging Solutions, Melville, NY). EM blocks, grids, and sections were selected randomly, sections were scanned systematically and an Eagle 4K CCD camera (FEI, Einhoven, The Netherlands) was used to record images. In NRK cells in which terminal endocytic compartments were pre-loaded with dextran-Texas Red^{TM} (DexTR), the density of DAMP

immunolabelling was recorded in 71 individual organelles co-immunolabelled with anti-TR antibodies (2012 gold particles) and 49 nuclei (2020 gold particles).

Quantitation of fluorescence microscopy

Fluorescence colocalization analysis was carried out with Imaris software (Imaris x64 v7.7.1, Bitplane Scientific Software, Zürich, Switzerland) using its automated thresholding algorithm on confocal images with detectors adjusted such that the peak intensity in each channel was just below saturation. Pearson's correlation coefficients and Manders' colocalization coefficients were calculated and used respectively to measure the overall association of two fluorescent probes and the fraction of the total fluorescence of one probe overlapping with a second [S13, S14]. The size distribution of organelles (Figure S1B) in 15 NRK cells from a single experiment was analysed with the Imaris software. The MR-negative, DexA-positive organelles were identified as 35.5% of the total of 2447 DexA-positive organelles, based on Manders'coefficient (M1) data shown in Figure S1D. None of these organelles had MR-channel fluorescence >1.5x maximum background fluorescence. Quantitation of LC3 fluorescence intensity to assess autophagy was carried out as previously described [S15]. To quantify nuclear translocation of TFEB-GFP, Cellomics ArrayScan™ software was used. After autofocussing using the Hoechst 33342 channel to detect the nuclei, the programme maps and outlines the area of each nucleus (Nuc) and a region extending outwards by an adjustable number of pixels (Cyt), representing a significant portion of the cytoplasm). Nuclear translocation (NucCyt difference) is calculated as the mean intensity of pixels in Nuc after subtracting the mean intensity of pixels in Cyt.

Supplemental References

S1. Bright, N.A., Reaves, B.J., Mullock, B.M., and Luzio, J.P. (1997). Dense core lysosomes can fuse with late endosomes and are re-formed from the resultant hybrid organelles. J Cell Sci *110 (Pt 17)*, 2027-2040.

S2. Reaves, B.J., Bright, N.A., Mullock, B.M., and Luzio, J.P. (1996). The effect of wortmannin on the localisation of lysosomal type I integral membrane glycoproteins suggests a role for phosphoinositide 3-kinase activity in regulating membrane traffic late in the endocytic pathway. J Cell Sci *109 (Pt 4)*, 749-762.

S3. Seaman, M.N., Harbour, M.E., Tattersall, D., Read, E., and Bright, N. (2009). Membrane recruitment of the cargo-selective retromer subcomplex is catalysed by the small GTPase Rab7 and inhibited by the Rab-GAP TBC1D5. J Cell Sci *122*, 2371-2382.

S4. Gordon, D.E., Mirza, M., Sahlender, D.A., Jakovleska, J., and Peden, A.A. (2009). Coiled-coil interactions are required for post-Golgi R-SNARE trafficking. EMBO Rep *10*, 851-856.

S5. Lange, P.F., Wartosch, L., Jentsch, T.J., and Fuhrmann, J.C. (2006). ClC-7 requires Ostm1 as a beta-subunit to support bone resorption and lysosomal function. Nature *440*, 220-223.

S6. Gomori, G. (1952). Microscopic histochemistry: principles and practice, (University of Chicago Press).

S7. Essner, E., and Novikoff, A.B. (1961). Localization of acid phosphatase activity in hepatic lysosomes by means of electron microscopy. J Biophys Biochem Cytol *9*, 773-784.

S8. Holt, S.J., and Hicks, R.M. (1961). The localization of acid phosphatase in rat liver cells as revealed by combined cytochemical staining and electron microscopy. J Biophys Biochem Cytol *11*, 47-66.

S9. Robinson, J.M., and Karnovsky, M.J. (1983). Ultrastructural localization of several phosphatases with cerium. J Histochem Cytochem *31*, 1197-1208.

S10. Liou, W., Geuze, H.J., and Slot, J.W. (1996). Improving structural integrity of cryosections for immunogold labeling. Histochem Cell Biol *106*, 41-58.

S11. Slot, J.W., and Geuze, H.J. (2007). Cryosectioning and immunolabeling. Nat Protoc *2*, 2480-2491.

S12. Bright, N.A., Gratian, M.J., and Luzio, J.P. (2005). Endocytic delivery to lysosomes mediated by concurrent fusion and kissing events in living cells. Curr Biol *15*, 360-365.

S13. Dunn, K.W., Kamocka, M.M., and McDonald, J.H. (2011). A practical guide to evaluating colocalization in biological microscopy. Am J Physiol Cell Physiol *300*, C723-742.

S14. Manders, E.M.M., Verbeek, F.J., and Aten, J.A. (1993). Measurement of Colocalization of Objects in Dual-Color Confocal Images. J Microsc-Oxford *169*, 375-382.

S15. Wartosch, L., Gunesdogan, U., Graham, S.C., and Luzio, J.P. (2015). Recruitment of VPS33A to HOPS by VPS16 Is Required for Lysosome Fusion with Endosomes and Autophagosomes. Traffic *16*, 727-742.
