# Supplementary figures and images for: Endolysosomes Are the Principal Intracellular Sites of Acid Hydrolase Activity
Source: Curr Biol. 2016 Sep 12;26(17):2233–45. doi: 10.1016/j.cub.2016.06.046 (PMC5026700; doi:10.1016/j.cub.2016.06.046)

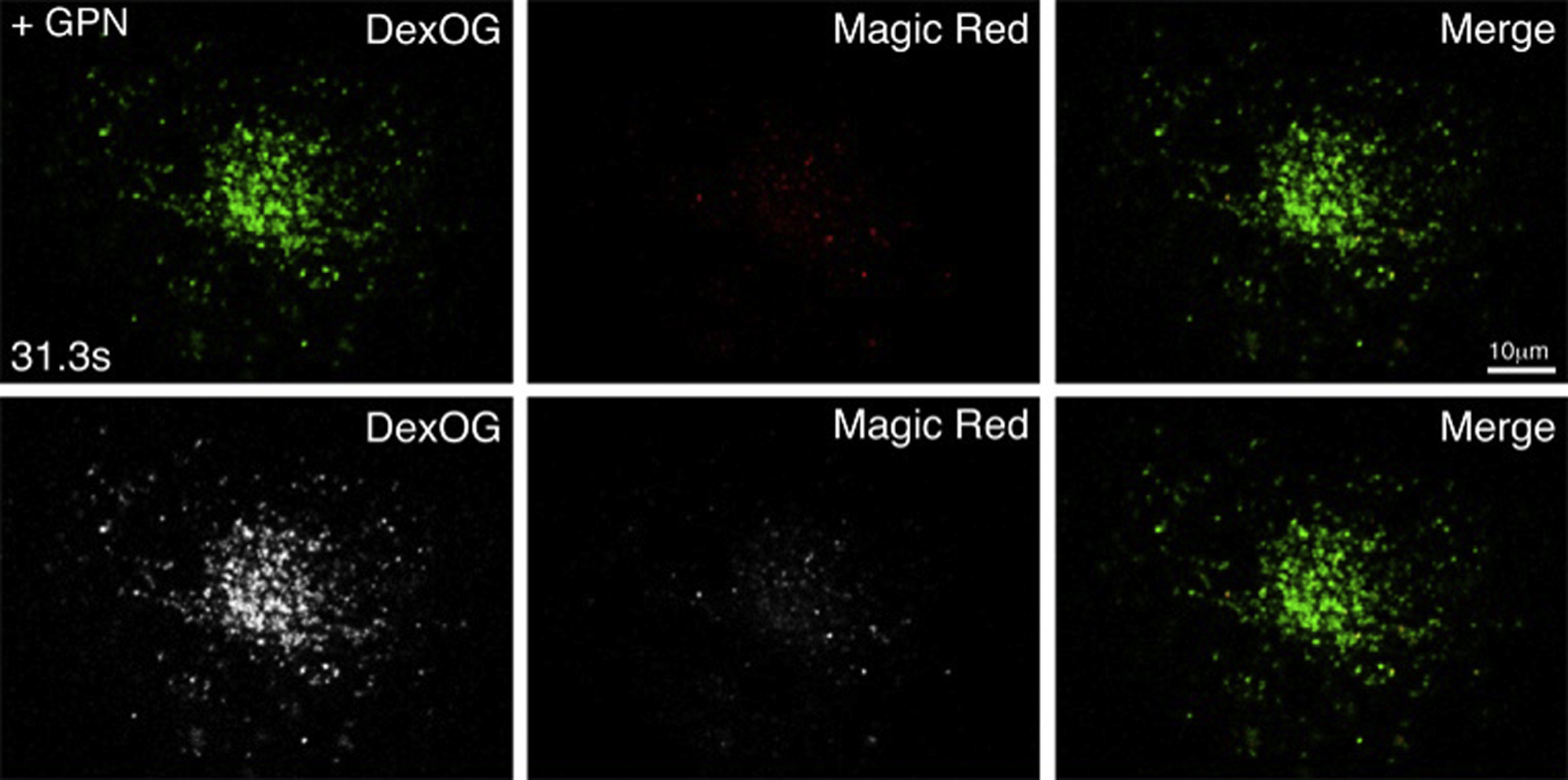

Supplement: Movie S1. Magic Red-Positive Endolysosomes Contain Active Cathepsin C — Terminal endocytic compartments of NRK cells were loaded with DexOG for 4 h followed by a 20 h chase in DexOG-free medium. Cathepsin-active endolysosomes were revealed by incubation with cathepsin B Magic Red substrate for 2 min and time-lapse images of the living cell collected on the confocal microscope. Addition of 200 μM GPN resulted in rapid dissipation of cresyl violet from the cathepsin-active endolysosomes but the DexOG (Mr 10,000) was retained in these organelles. Upper panels: false color; lower panels: grayscale. [file mmc2.jpg]

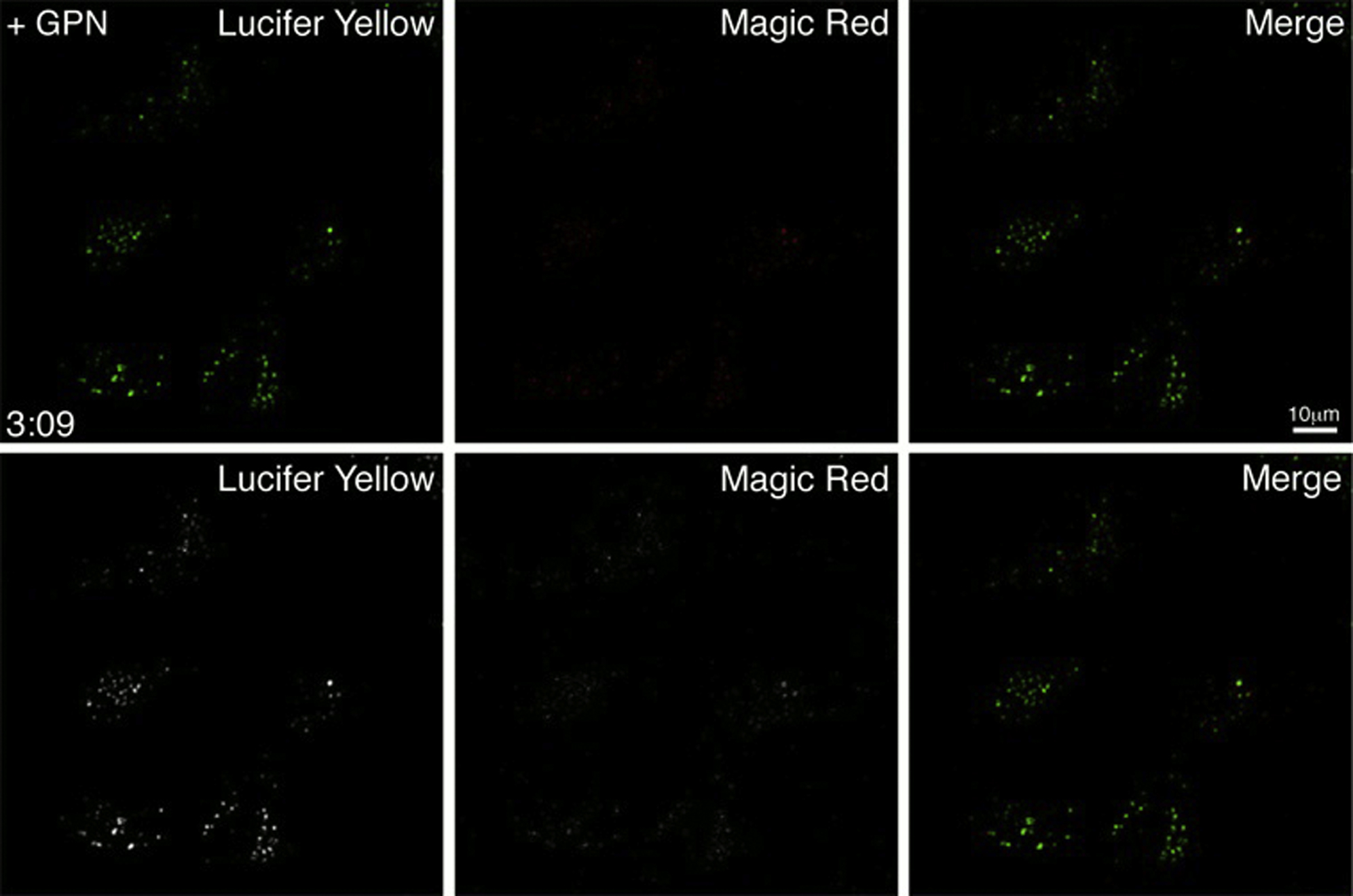

Supplement: Movie S2. Lucifer Yellow Is Dissipated from a Subpopulation of Organelles by Incubation with GPN — Terminal endocytic compartments of NRK cells were loaded with LucY for 4 h followed by a 20 h chase in LucY -free medium and the cells then incubated for 2 min with cathepsin B Magic Red substrate. Time-lapse images of the living cells were collected on the confocal microscope. Addition of 200 μM GPN resulted in rapid dissipation of cresyl violet and LucY from the cahepsin-active endolysosomes but LucY was retained in a population of the terminal endocytic organelles revealing that it also resided in organelles that were not catalytically active for cathepsin C. Scale Bar: 10 μm. Upper panels: false color; lower panels: grayscale. [file mmc3.jpg]

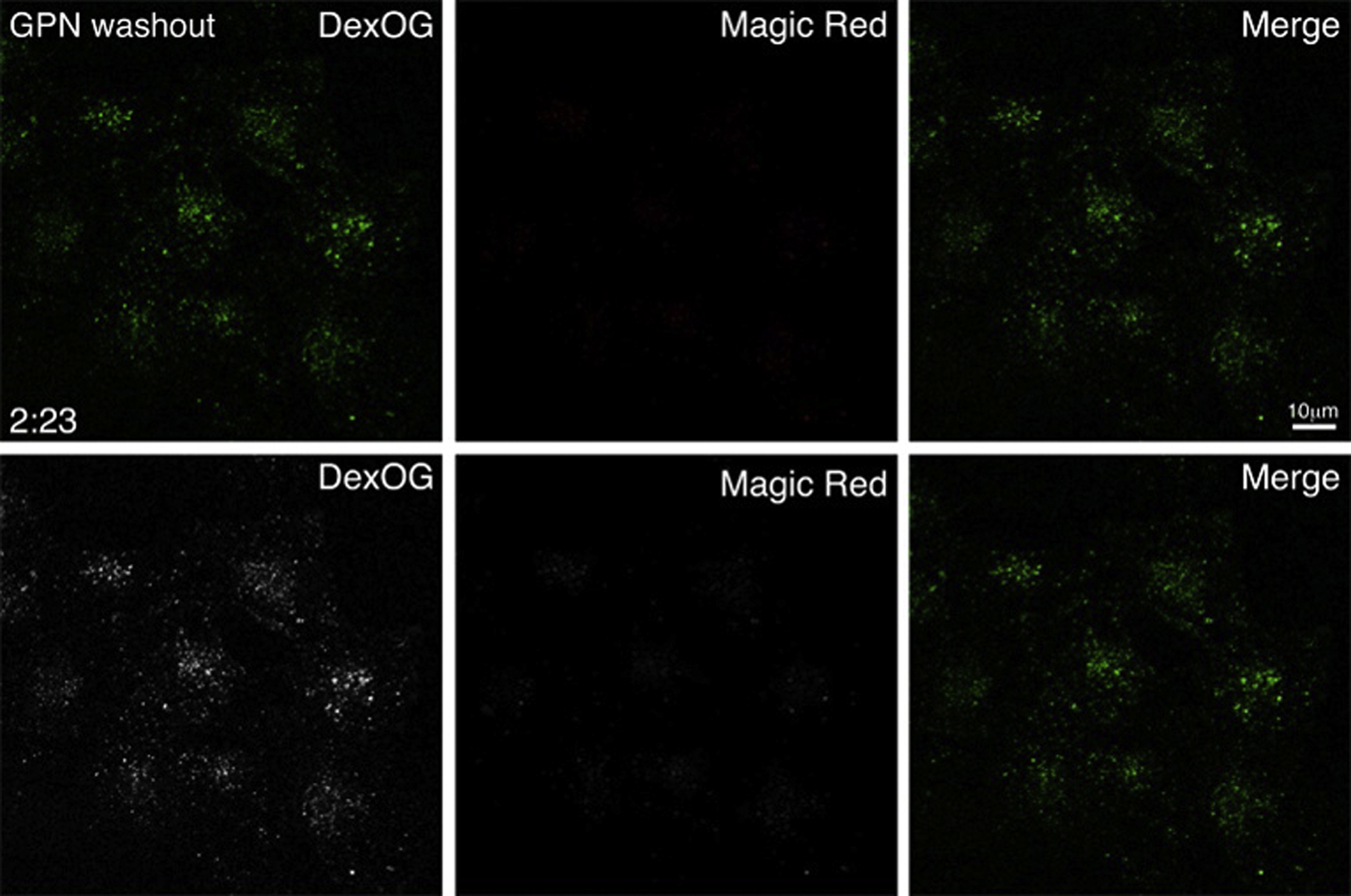

Supplement: Movie S3. Cathepsin B Catalytic Activity Recovers after GPN Washout — Terminal endocytic compartments of NRK cells were loaded with DexOG for 4 h followed by a 20 h chase in DexOG-free medium. Cathepsin-active endolysosomes were revealed by incubation with cathepsin B Magic Red substrate for 2 min and images of the living cells collected on the confocal microscope. Addition of 200 μM GPN resulted in rapid dissipation of cresyl violet from the cathepsin-active endolysosomes but the DexOG was retained. Washout of the GPN with medium containing cathepsin B Magic Red substrate showed that cathepsin B was retained and remained catalytically active upon removal of GPN. Upper panels: false color; lower panels: grayscale. [file mmc4.jpg]

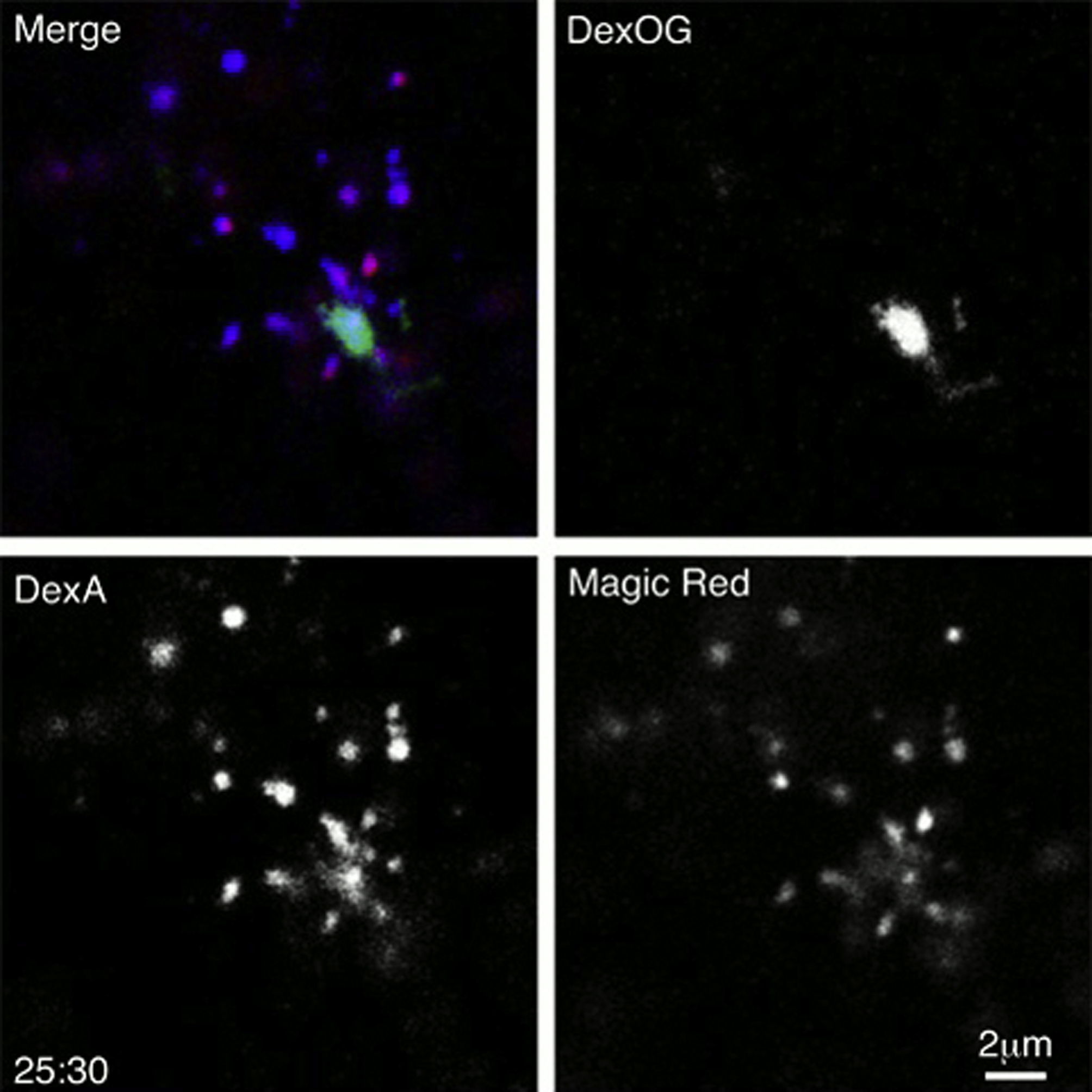

Supplement: Movie S4. Organelle Kissing Causes Cathepsin Activation in the Newly Forming Endolysosome — Terminal endocytic compartments of NRK cells were pre-loaded with DexA for 4 h followed by a 20 h chase in DexA-free medium and then late endosomes were loaded with DexOG by uptake for 10 min followed by a 5 min chase in DexOG-free medium containing cathepsin B Magic Red substrate. Time-lapse confocal microscopy of the living cell revealed that transient fusions of a DexOG-positive late endosome with DexA-laden terminal endocytic compartments resulted in the gradual acquisition of DexA (blue) in the DexOG-positive organelle (green) and a subsequent rise in cresyl violet fluorescence from the cleaved Magic Red substrate (red). [file mmc5.jpg]

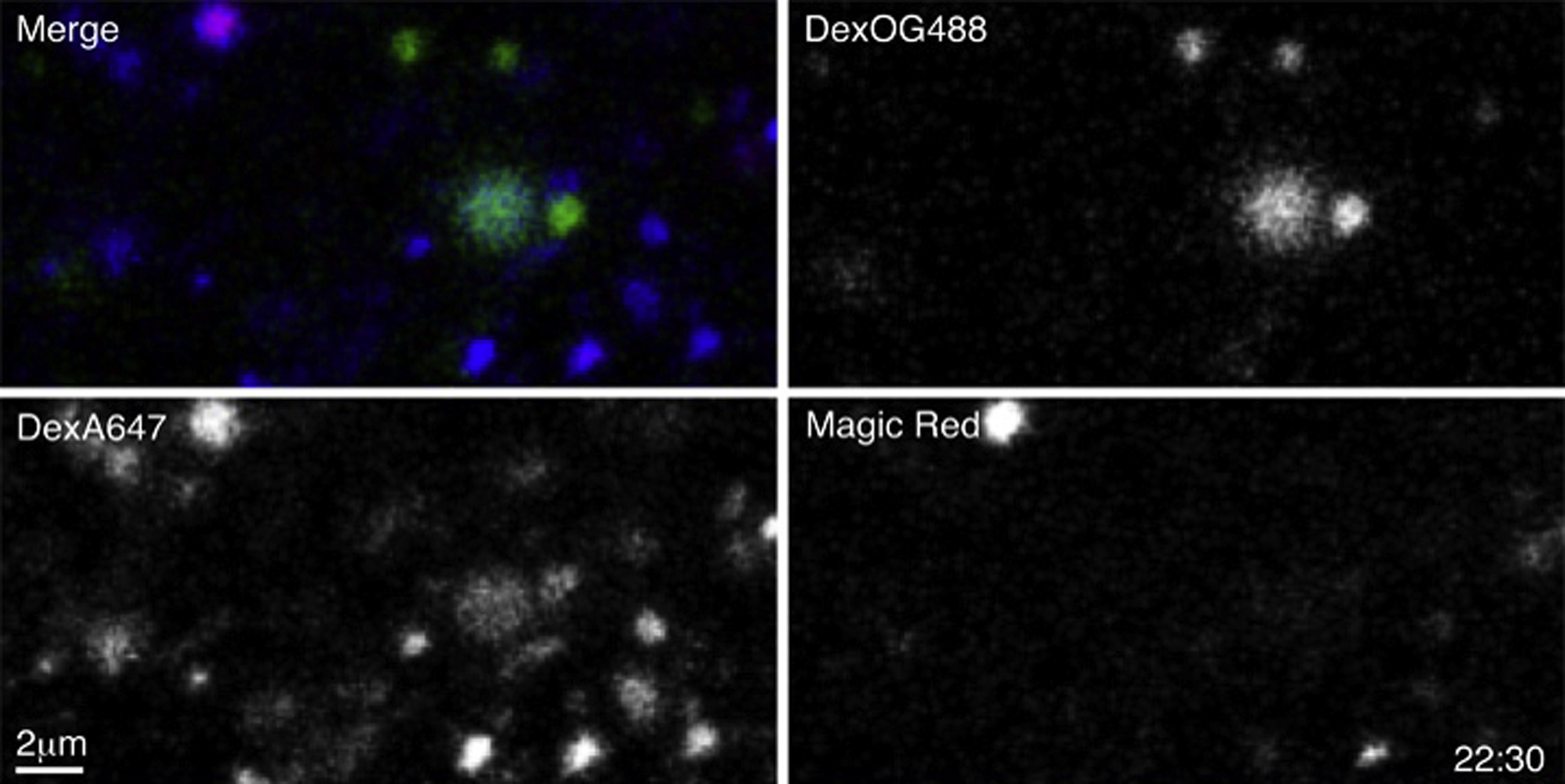

Supplement: Movie S5. Emergence of Tubules after Formation of a Cathepsin-Active Endolysosome — Terminal endocytic compartments of NRK cells were loaded with DexA for 4 h followed by a 20 h chase in DexA-free medium and late endosomes were loaded with DexOG by uptake for 10 min followed by a 5 min chase in DexOG-free medium containing cathepsin B Magic Red substrate. Time-lapse confocal microscopy of a living cell revealed a complete fusion between a DexOG-laden organelle and DexA-laden organelle(s) and subsequent rise in cresyl violet fluorescence from the cleaved cathepsin B Magic Red substrate. Extrusion and detachment of a re-formation tubule (arrowheads) containing each of the 3 fluorochromes was seen after the fusion. [file mmc6.jpg]

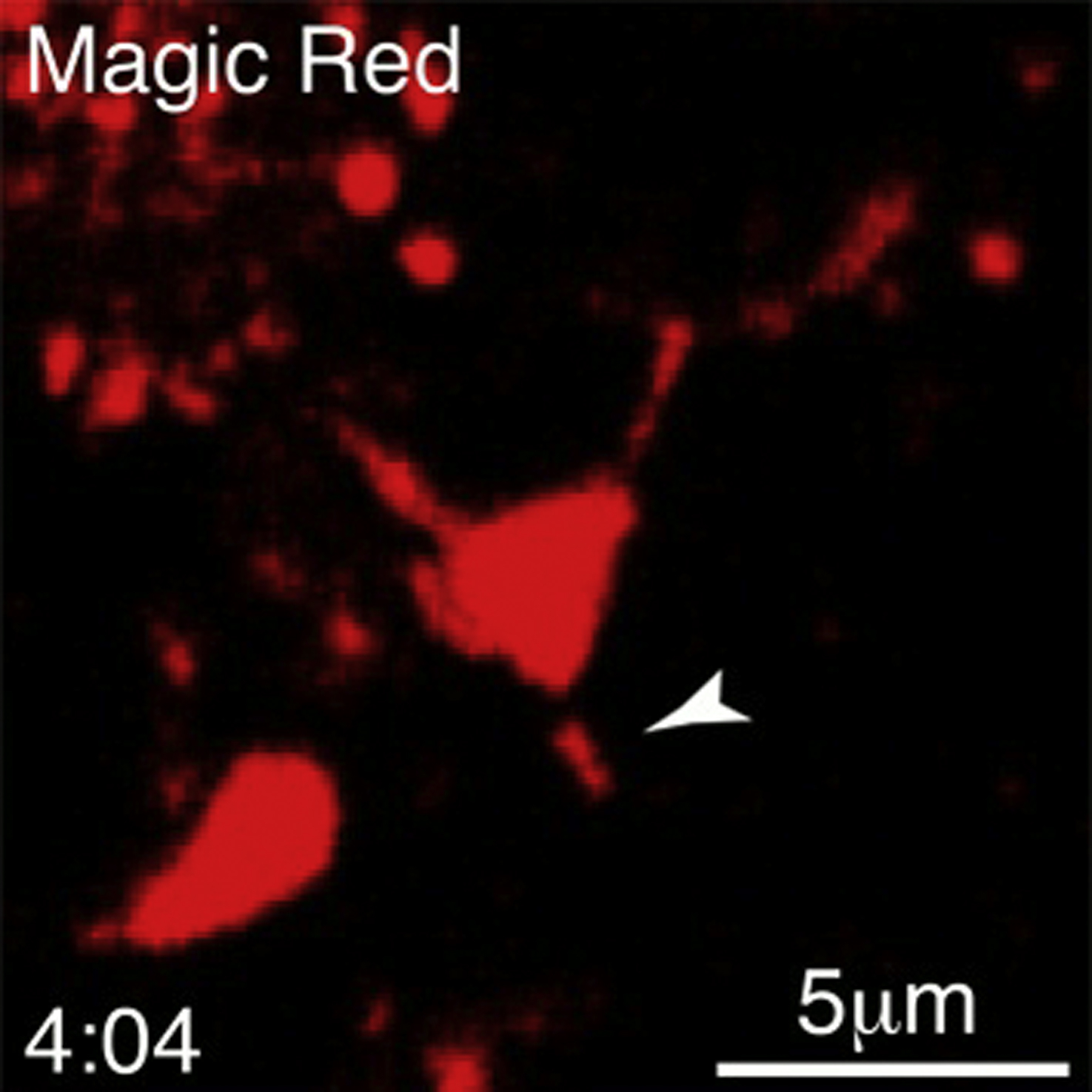

Supplement: Movie S6. Tubulation of Sucrosomes after Invertase Uptake — Sucrosomes were formed in NRK cells by endocytosis of 30 mM sucrose for 24 h and subsequently incubated with medium containing 0.5mg/ml invertase for 1 h followed by the addition of cathepsin B Magic Red substrate for 2 min. Incubation with cathepsin B Magic Red substrate revealed that the swollen sucrosomes were cathepsin-active and a time-lapse series of images collected on the confocal microscope showed that addition of invertase resulted in extensive tubulation of the cathepsin-active sucrosomes and in some instances tubules could be observed detaching from the parent sucrosome (arrowhead). [file mmc7.jpg]
