# Supplementary material for: Endolysosomes Are the Principal Intracellular Sites of Acid Hydrolase Activity
Source: Curr Biol. 2016 Sep 12;26(17):2233–45. doi: 10.1016/j.cub.2016.06.046 (PMC5026700; doi:10.1016/j.cub.2016.06.046)
Supplement: Document S2. Article plus Supplemental Information [file mmc8.pdf]

Article

Current Biology

Endolysosomes Are the Principal Intracellular Sites of Acid Hydrolase Activity

Highlights

- Late endosome-lysosome fusion creates acidic, cathepsin-active endolysosomes
- Terminal storage lysosomes are cathepsin inactive and not acidic
- Fusion events creating endolysosomes precede the onset of cathepsin activity
- A lysosome regeneration cycle links endolysosomes and terminal storage lysosomes

Authors

Nicholas A. Bright, Luther J. Davis, J. Paul Luzio

Correspondence

nab21@cam.ac.uk (N.A.B.), jpl10@cam.ac.uk (J.P.L.)

In Brief

Bright et al. provide evidence that the terminal endocytic compartment is composed of acid-hydrolase-active, acidic endolysosomes and acid hydrolase-inactive, non-acidic, terminal storage lysosomes, which are linked and function in a lysosome regeneration cycle.

Bright et al., 2016, Current Biology *26*, 2233–2245
September 12, 2016 © 2016 The Author(s). Published by Elsevier Ltd.
http://dx.doi.org/10.1016/j.cub.2016.06.046

CellPress

Current Biology
Article

CellPress

Endolysosomes Are the Principal Intracellular Sites of Acid Hydrolase Activity

Nicholas A. Bright,[1,*] Luther J. Davis,[1] and J. Paul Luzio[1,*]
[1]Cambridge Institute for Medical Research and Department of Clinical Biochemistry, University of Cambridge School of Clinical Medicine, Wellcome Trust/MRC Building, Cambridge Biomedical Campus, Hills Road, Cambridge CB2 0XY, UK
*Correspondence: nab21@cam.ac.uk (N.A.B.), jpl10@cam.ac.uk (J.P.L.)
http://dx.doi.org/10.1016/j.cub.2016.06.046

SUMMARY

The endocytic delivery of macromolecules from the mammalian cell surface for degradation by lysosomal acid hydrolases requires traffic through early endosomes to late endosomes followed by transient (kissing) or complete fusions between late endosomes and lysosomes. Transient or complete fusion results in the formation of endolysosomes, which are hybrid organelles from which lysosomes are re-formed. We have used synthetic membrane-permeable cathepsin substrates, which liberate fluorescent reporters upon proteolytic cleavage, as well as acid phosphatase cytochemistry to identify which endocytic compartments are acid hydrolase active. We found that endolysosomes are the principal organelles in which acid hydrolase substrates are cleaved. Endolysosomes also accumulated acidotropic probes and could be distinguished from terminal storage lysosomes, which were acid hydrolase inactive and did not accumulate acidotropic probes. Using live-cell microscopy, we have demonstrated that fusion events, which form endolysosomes, precede the onset of acid hydrolase activity. By means of sucrose and invertase uptake experiments, we have also shown that acid-hydrolase-active endolysosomes and acid-hydrolase-inactive, terminal storage lysosomes exist in dynamic equilibrium. We conclude that the terminal endocytic compartment is composed of acid-hydrolase-active, acidic endolysosomes and acid hydrolase-inactive, non-acidic, terminal storage lysosomes, which are linked and function in a lysosome regeneration cycle.

INTRODUCTION

Lysosomes were first discovered by De Duve and colleagues over 60 years ago, and their importance in the degradation of endocytosed, phagocytosed, and autophagocytosed macromolecules was soon recognized [1]. For a long time, lysosomes were simply regarded as endpoint, degradatively active compartments. Their morphological heterogeneity was recognized early, but only over the past two decades have their dynamic, functional interactions with other organelles of the secretory, endocytic, and autophagic pathways been properly appreciated. In addition, it has recently been shown that lysosomes play a central role in nutrient sensing and signaling to the nucleus, to mediate cellular responses to starvation and the regulation of energy metabolism [2–5]. The delivery of endocytosed macromolecules to lysosomal acid hydrolases for degradation occurs as a result of transient (kissing or kiss-and-run) and complete fusion events between lysosomes and late endosomes/multivescular bodies (MVBs) [6–8]. A consequence of these transient and complete fusion events is the formation of hybrid organelles, now called endolysosomes [8], which have properties intermediate between late endosomes and lysosomes, and from which lysosomes are re-formed. While the presence of cathepsins in endolysosomes has been shown in cells [9] and cell-free systems by immuno-electron microscopy (immuno-EM); reviewed in [7]), the extent to which endolysosomes or more terminal lysosomal compartments are involved in hydrolysis of macromolecules has not been determined. In the present study, we have identified the late endocytic organelles in which acid hydrolases are catalytically active by using synthetic membrane-permeable cathepsin substrates, which liberate fluorescent reporters that remain within the organelle upon proteolytic cleavage, as well as by acid phosphatase (AcPase) cytochemistry. Using fluorescence microscopy and EM, we observed that endolysosomes are the principal organelles in which substrates are hydrolyzed and that accumulate acidotropic probes. They could be distinguished from terminal storage lysosomes, which are acid hydrolase inactive and do not accumulate acidotropic probes. We have also used live-cell microscopy to study the dynamic interaction of late endosomes, endolysosomes, and acid-hydrolase-inactive terminal storage lysosomes, to determine the relationship of organelle kissing to the development of acid hydrolase activity and to study the re-formation of terminal storage lysosomes from endolysosomes.

RESULTS

Fluorescent Cresyl Violet Is Liberated from Hydrolyzed Cathepsin Substrates Only in a Subpopulation of Terminal Endocytic Compartments

Terminal endocytic compartments of cultured normal rat kidney (NRK) fibroblasts were first loaded with a DexOG (dextran-Oregon green 488) conjugate using a 4 hr pulse/20 hr chase protocol to establish an equilibrium of labeling between lysosomes and endolysosomes as previously described [6]. The cells were then incubated with the membrane permeable cathepsin B MR (Magic Red) substrate that, upon hydrolysis, liberates

CrossMark

This is an open access article under the CC BY license (http://creativecommons.org/licenses/by/4.0/).

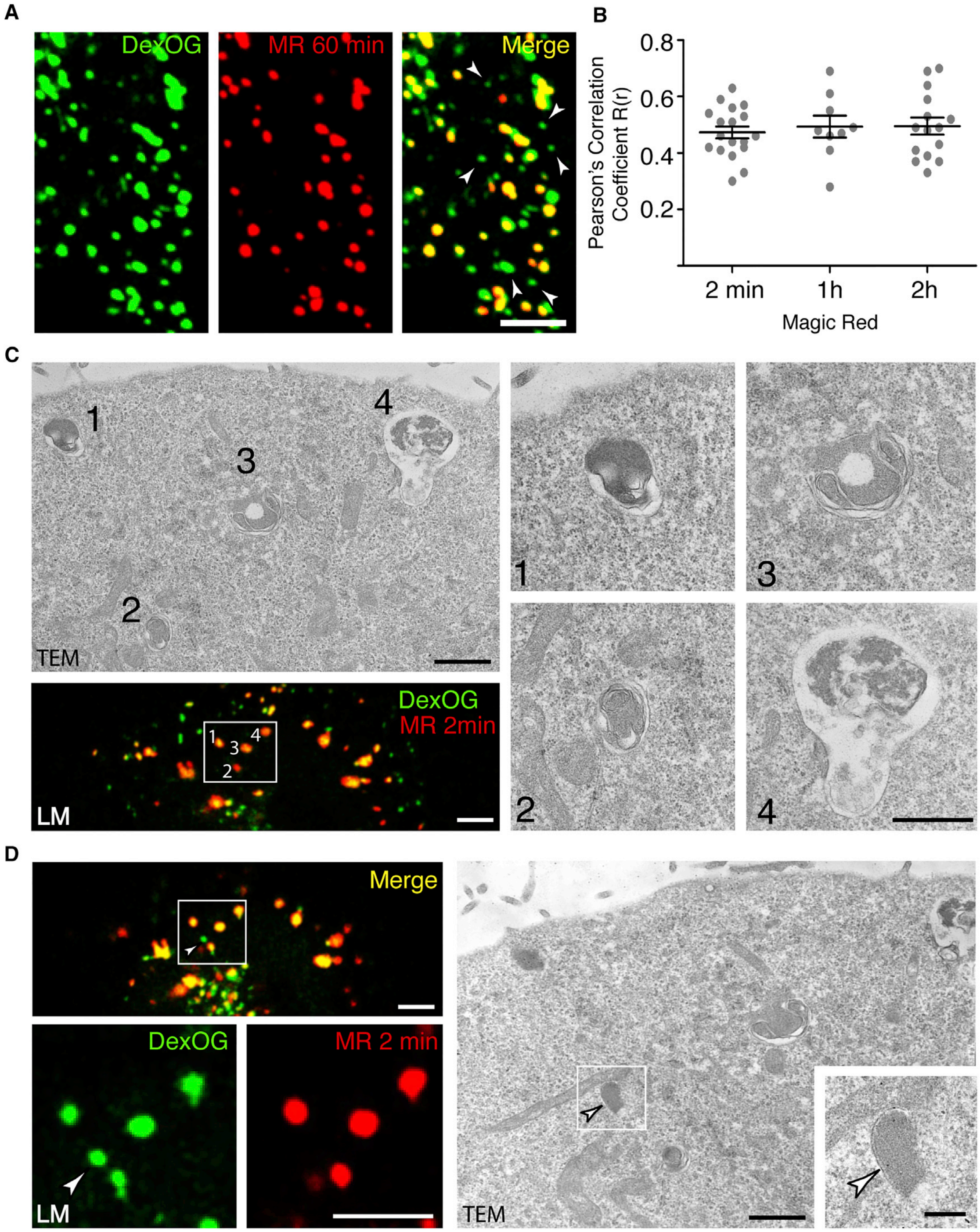

Figure 1. Endolysosomes Are the Principal Sites of Cathepsin B Catalytic Activity

Terminal endocytic compartments of NRK cells were loaded with DexOG for 4 hr followed by a 20 hr chase in DexOG-free medium.

(A) Part of a single cell showing that cresyl violet fluorescence (red) was present only in a subset of terminal endocytic organelles containing pre-loaded dextran after incubation with cathepsin B MR substrate for 1 hr. Arrowheads show examples where terminal endocytic organelles have not hydrolyzed the MR substrate.

(B) Pearson's correlation coefficients (R(r); mean ± SEM of nine or more cells within a single experiment) for colocalization of pre-loaded DexOG and liberated cresyl violet fluorescence after hydrolysis of cathepsin B MR substrate for different times (see also Figure S1A).

(C) CLEM of the cathepsin-active organelles. DexOG-positive, MR-positive organelles were identified by confocal microscopy before processing for TEM to show their ultrastructure. The boxed region of the confocal image and corresponding section in the TEM shows the ultrastructure of four cathepsin-active endolysosomes. The enlargements show that these organelles contain multi-lamellar intralumenal membranes and electron dense content.

(legend continued on next page)

membrane-impermeable fluorescent cresyl violet within organelles containing catalytically active cathepsin B [10, 11]. Using confocal microscopy either of live cells or after fixing cells with paraformaldehyde, the liberation of cresyl violet fluorescence in some of the DexOG-positive organelles was detected within 30 s of the addition of cathepsin B MR substrate (data not shown). Even after 60 min, with cathepsin B MR substrate present in the medium throughout, only some of the DexOG-positive compartments were labeled (Figures 1A and S1A). There was no significant difference in the overall colocalization of cresyl violet and DexOG between cells incubated with the cathepsin B MR substrate for 2 min or 2 hr (Figure 1B). To visualize the ultrastructure of the cresyl violet-containing organelles, we carried out correlative light and electron microscopy (CLEM) after incubation with cathepsin B MR substrate for 2 min, to ensure that the population of cresyl violet-containing (MR-positive) organelles included few, if any, re-formed lysosomes. The majority of the MR-positive organelles were characterized by the presence of multi-lamellar intralumenal membranes and an electron-dense lumenal content containing electron-lucent sub-domains, but we also observed electron-lucent vacuolar organelles containing intralumenal membrane, intralumenal vesicles (ILVs) and diffuse electron-dense content (Figure 1C). Thus, there was a diverse array of ultrastructural morphology among the organelles demonstrating acid hydrolase activity. In contrast, Figure 1D shows a different confocal plane to that illustrated in Figure 1C, revealing an organelle containing DexOG, but not cresyl violet which, in transmission electron microscopy (TEM), possessed the characteristic electron dense, granular morphology of a dense core lysosome. The smaller size of this dense core lysosome compared to the MR-positive organelles is consistent with data from confocal microscopy showing the size distribution of MR-positive and MR-negative terminal endocytic compartments (Figure S1B).

To confirm that our observations were neither cell type dependent nor specific for only one cathepsin, we repeated our confocal microscopy experiments with HeLa and MCF7 cells as well as with a cathepsin L MR substrate (Figures S1C–S1E). In all cases, only some of the fluorescent dextran-positive terminal endocytic compartments were MR positive. We interpreted the MR experiments combined with the CLEM as showing two types of terminal endocytic compartment: cathepsin-active organelles (predicted to be endolysosomes) and cathepsin-inactive terminal lysosomes. In our confocal fluorescence microscopy experiments, we assessed colocalization by calculating Pearson's and Manders' coefficients and used the latter (see the Supplemental Experimental Procedures), to estimate the proportion of fluorescent dextran-positive terminal endocytic compartments that were cathepsin-active endolysosomes as 67.4% ± 4.6% in NRK cells, 46.8% ± 8.7% in HeLa cells, and 64.1% ± 3.3% in MCF7 cells (see values for M1 in Figure S1E). The remainder of the fluorescent dextran-positive terminal endocytic compartments were cathepsin-inactive terminal lysosomes. The lack of hydrolysis of cathepsin MR substrates in the cathepsin-inactive terminal lysosomes was not due to a problem of substrate access or the absence of enzyme in these compartments, since incubation of the cells with the cathepsin B MR substrate in a pH 5 buffer in the presence of nigericin and monensin to equilibrate pH across compartments allowed us to capture images of the appearance of cresyl violet fluorescence in all of a cell's DexOG-positive terminal endocytic compartments before it dissipated across the organelles' limiting membranes within 5 min (Figure S1F; see also immuno-EM of cathepsin D in MCF7 cells below).

Further evidence for a distinction between cathepsin-active endolysosomes and inactive terminal lysosomes came from experiments with the membrane permeable cathepsin C substrate GPN (glycyl-D-phenylalanine-2-naphthylamide) [12]. When GPN is added to cultured cells, its hydrolysis in organelles in which cathepsin C is catalytically active causes a transient permeabilization of the limiting membrane and the leak of small molecular mass solutes (relative molecular weight [Mr] <10,000) [13–16]. In NRK cells in which terminal endocytic compartments were pre-loaded with DexOG, we observed a rapid dissipation of cresyl violet (Mr 321), but not DexOG (Mr 10,000) from the endolysosomes when GPN was added after the cathepsin B MR substrate (Figures 2A, 2B, and S2A; Movie S1). The GPN treatment also failed to dissipate DexOG from the cathepsin-inactive terminal lysosomes. In addition, GPN treatment of NRK cells in which terminal endocytic compartments were pre-loaded by fluid phase endocytosis of Lucifer Yellow (LucY, Mr 457) showed loss of both cresyl violet and LucY from the MR-positive endolysosomes, but not from the MR-negative terminal endocytic compartments (Movie S2; Figure S2B). The transient nature of the GPN effect allowed us to show that following washout of the GPN, individual endolysosomes, which had leaked cresyl violet, rapidly regained the ability to hydrolyze the cathepsin B MR substrate (Figure 2C; Movie S3).

Cathepsin-Active Endolysosomes Are Acid Phosphatase Positive and Can Degrade Endocytosed Macromolecules

CLEM was used to examine whether the MR-positive compartments in NRK cells were also AcPase positive by applying EM cytochemistry. For these experiments, terminal endocytic compartments of NRK cells were loaded with DexOG and BSA-gold (BSA conjugated to 5 nm colloidal gold) [9] before the cells were incubated with the cathepsin B MR substrate for 2 min. As expected, the MR-positive endolysosomes were labeled with BSA-gold and these compartments were also AcPase-positive (Figure 3A). The AcPase-positive endolysosomes, which account for 61.8% ± 5.7% (three experiments) of the BSA-gold positive organelles were also positive for cation-independent mannose 6-phosphate receptor (MPR) and lysobisphosphatidic acid/bis(monoacylglycerol)phosphate (LBPA/BMP) by immuno-EM (Figures S3A and S3B). They were sometimes closely

(D) An image from an adjacent confocal plane to that shown in (C) with enlargements and corresponding TEM image of the boxed region identifying a DexOG-positive, MR-negative terminal endocytic organelle (arrowhead) within the constellation of endolysosomes. The TEM image of this organelle reveals it to be a granular dense core lysosome (boxed region of TEM image and inset).

Scale bars represent 5 μm (A), 5 μm (C, light microscopy [LM]), 1 μm (C, TEM), 500 nm (C, TEM enlargements), 5 μm (D, LM), 1 μm (D, TEM), and 200 nm (D, TEM inset). See also Figure S1.

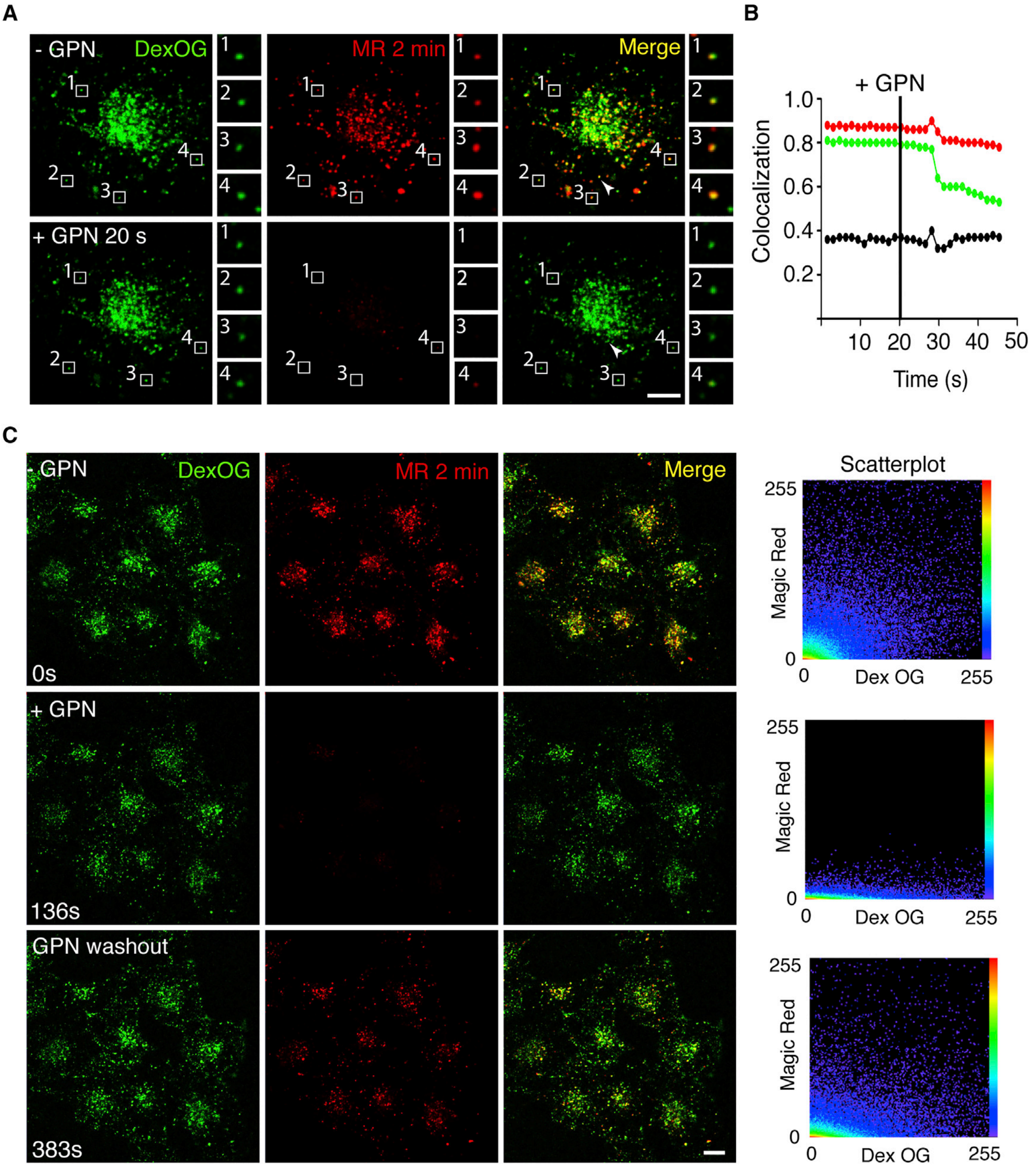

Figure 2. Magic-Red-Positive Endolysosomes Contain Active Cathepsin C and Can Recover Enzyme Activity after GPN Treatment

Terminal endocytic compartments of NRK cells were loaded with DexOG for 4 hr followed by a 20 hr chase in DexOG-free medium. (A) Cathepsin-active endolysosomes were revealed by incubation with cathepsin B MR substrate for 2 min, and images of the living cells were collected on the confocal microscope (top, single cell with enlargements of four endolysosomes). Addition of 200 μM GPN (bottom) resulted in rapid dissipation of cresyl violet from the cathepsin-active endolysosomes (loss of cresyl violet from four endolysosomes shown in enlargements) but the DexOG (Mr 10,000) was retained in these organelles. See also Figure S2A and Movie S1. The motility of transiently permeabilized endolysosomes (e.g., the organelle identified by arrowhead) was unaffected (see Movie S1).

(B) Pearson's (R(r); black) and Manders' (M1, green, DexOG:MR and M2, red, MR:DexOG) correlation coefficients for colocalization of pre-loaded DexOG and liberated cresyl violet fluorescence (MR) before and after addition (vertical black line) of 200 μM GPN to the cell shown in (A) and Movie S1.

(C) Cathepsin-active endolysosomes were revealed by incubation with Magic Red (MR) substrate for 2 min (top row), and images of the living cells were collected on the confocal microscope. Addition of 200 μM GPN (middle row) resulted in rapid dissipation of cresyl violet from the cathepsin-active endolysosomes, but the DexOG was retained as described above. Washout of the GPN with medium containing cathepsin B MR substrate (bottom) showed that cathepsin B was retained and remained catalytically active upon removal of GPN. The associated scatterplots of the merged images clearly show the dissipation and re-acquisition of cresyl violet fluorescence upon addition of GPN and subsequent washout. See also Movie S3. Scale bars represent 10 μm. See also Figure S2.

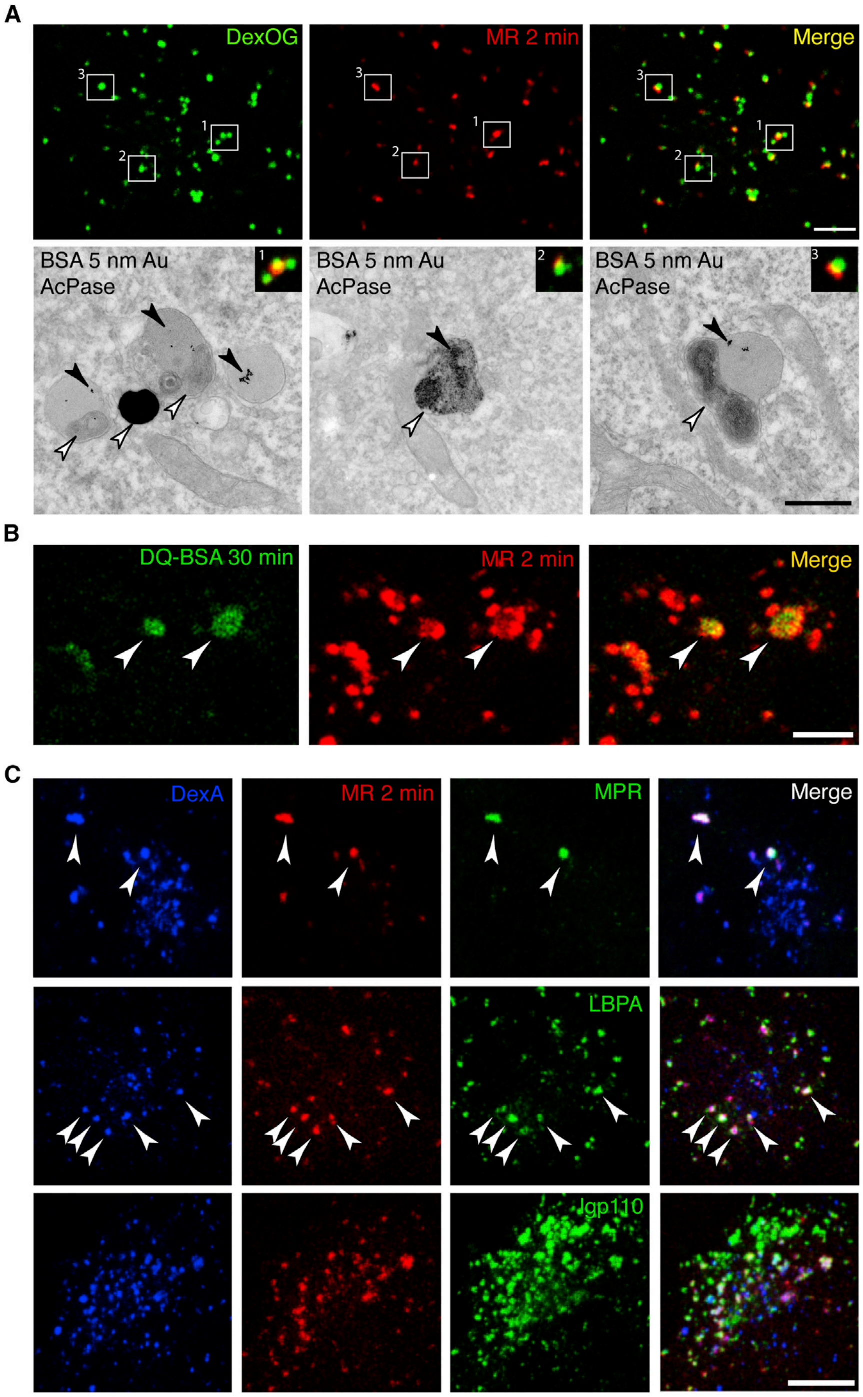

Figure 3. Characterization of Endolysosomes

(A) Terminal endocytic compartments of NRK cells were loaded with DexOG and BSA-gold for 4 hr followed by a 20 hr chase in DexOG/BSA-gold-free medium and incubation with cathepsin B MR substrate for 2 min. DexOG/MR-positive compartments were identified by confocal microscopy (top) and CLEM with AcPase cytochemistry, performed using β-glycerophosphate and cerium chloride capture for 2 hr prior to processing for TEM. This revealed cerium phosphate deposition in AcPase-active organelles (bottom, white arrowheads). BSA-gold (bottom, black arrowheads) labeled catalytically active endolysosomes and AcPase-inactive granular, dense core lysosomes; see also Figure S3C).

(B) Confocal fluorescence microscopy of NRK cells that had endocytosed DQ-BSA for 30 min and were then incubated with cathepsin B MR substrate for 2 min. Proteolytically cleaved DQ-BSA is indicated by white arrowheads and colocalizes with cresyl violet (MR).

(C) Confocal immunofluorescence microscopy of MR-positive organelles (red) in NRK cells preloaded with DexA (blue), to label terminal endocytic compartments and immunolabeled with antibodies against the cation-independent mannose 6-phosphate receptor (MPR), lysobisphosphatidic acid (LBPA), or lysosomal glycoprotein 110 (lgp110) followed by Alexa-488-conjugated secondary antibodies (green). Examples of cathepsin B catalytically active organelles labeled with antibodies to MPR and LBPA indicated by arrowheads. Extensive colocalization of both DexA and cresyl violet (MR) with lgp110 was observed.

Scale bars represent 10 μm (A, top), 500 nm (A, bottom TEM), and 10 μm (B and C). See also Figure S3.

associated with AcPase-negative, BSA-gold-positive terminal lysosomes (Figure 3A, box 1, lower-left panel, and Figure S3C). This association is consistent with observations from previous live-cell experiments in which several lysosomes were often seen very close to endolysosomes formed by earlier kissing events between endosomes and lysosomes (see, e.g., Movie 2 in [6]). Although we saw many instances in which AcPase reaction product was distributed throughout a BSA-gold-positive organelle (Figure 3A, box 2, lower-middle panel), we sometimes observed a region of AcPase reaction product within the same limiting membrane as an electron dense BSA-gold-positive region, as if a terminal AcPase-negative lysosome had recently fused with an endolysosome (Figure 3A, box 3, lower-right panel). The co-existence of AcPase-positive and AcPase-negative regions within the same endolysosome limiting membrane, despite the AcPase reaction being carried out at acid pH, could also imply that there are luminal regions in which the enzyme is present although inactive, but this was not explored further. Images showing that only some BSA-gold-positive organelles were AcPase positive were also obtained from MCF7 cells (Figure S3D). The availability of an antibody to human cathepsin D that was usable for immuno-EM enabled us to show that terminal endocytic organelles containing BSA-gold in MCF7 cells also contained immunoreactive cathepsin D irrespective of whether they were AcPase positive or negative, with 62.8% ± 1.5% (three experiments) of the immunoreactive cathepsin D molecules

CellPress

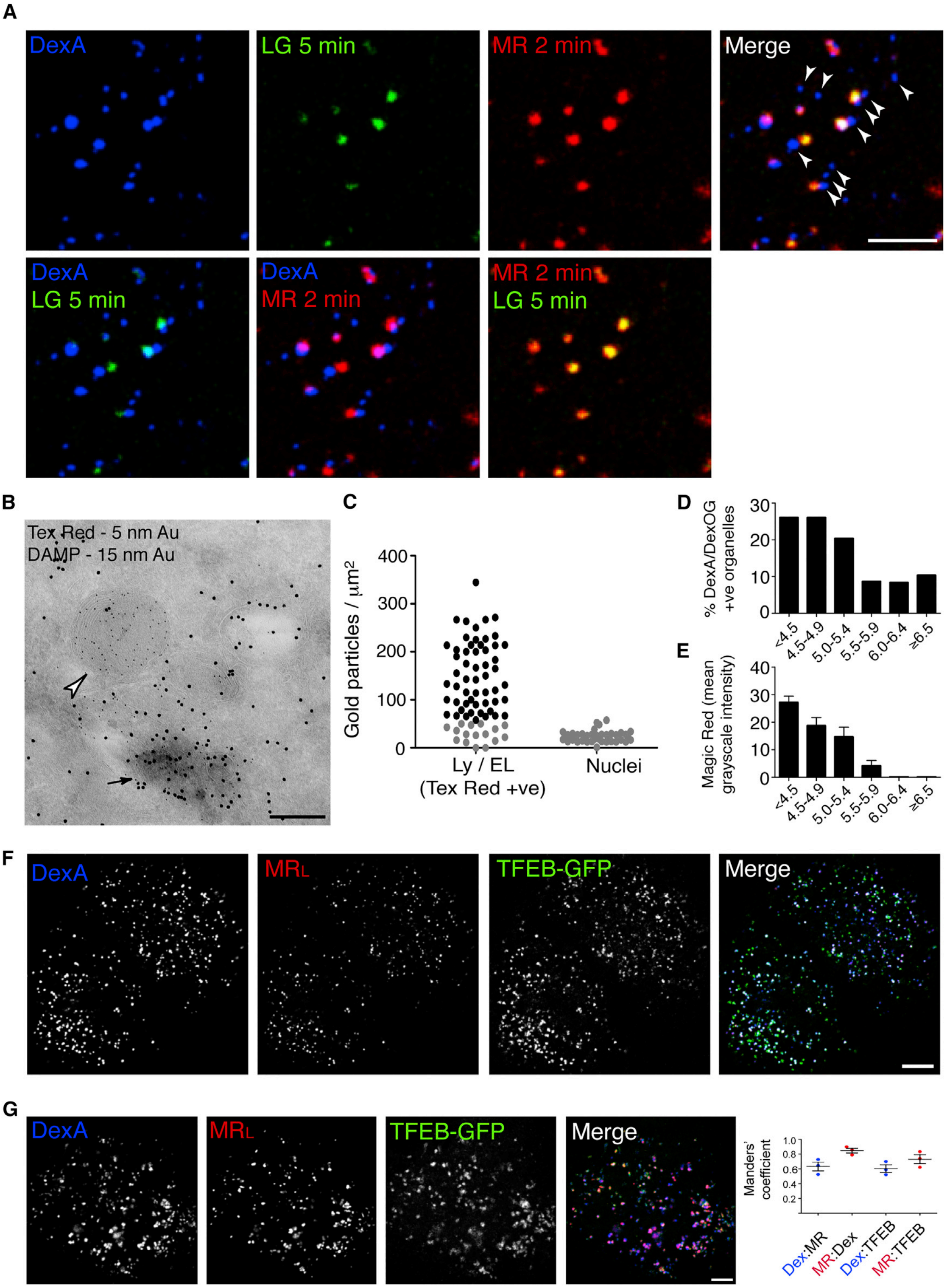

Figure 4. Cathepsin-Active Endolysosomes Accumulate Acidotropic Molecules but Terminal Lysosomes Do Not

(A) Confocal fluorescence microscopy images of cathepsin-active, acidic endolysosomes in a single NRK cell, in which terminal endocytic compartments were pre-loaded with DexA, revealed by incubation with cathepsin B MR substrate for 2 min followed by addition of 50 nM LysoTracker Green (LG) for 5 min. The merge shows that some DexA-positive compartments were both MR negative and LG negative (white arrowheads, see also Figure S4).

(B) Immuno-EM of DAMP accumulation in acidic organelles. NRK cells were pre-loaded with DexTR (5 nm gold) to label terminal endocytic compartments before incubation with 30 µM DAMP (15 nm gold) for 30 min to identify acidic organelles. An endolysosome has accumulated the acidotropic DAMP (arrow), but the adjacent terminal lysosome (arrowhead) has not.

(legend continued on next page)

associated with BSA-gold-positive organelles being in the AcPase-negative lysosomes (Figure S3D).

To confirm that the MR-positive endolysosomes are the site of hydrolysis of endocytosed macromolecules, we incubated NRK cells with DQ-BSA (DQ Green BSA), an endocytic probe that becomes fluorescent upon proteolytic cleavage [11, 17], before adding the cathepsin B MR substrate to the culture medium. Green fluorescent signal from the hydrolyzed DQ-BSA was first detected after 15 min of endocytic uptake (data not shown) and was clearly observed by 30 min (Figure 3B). The overlap of the green fluorescent signal with the cresyl violet increased when the cells were incubated with DQ-BSA for longer times (data not shown) and was consistent with the MR-positive endolysosomes being the site of proteolytic degradation of the endocytosed DQ-BSA.

Cathepsin-Active Endolysosomes Contain Markers Characteristic of Both Late Endosomes and Lysosomes

Fluorescence from the cresyl violet product of cathepsin MR substrate hydrolysis remains clearly visible for only 45–60 min after paraformaldehyde fixation. Nevertheless, we were able to use triple-labeling fluorescence confocal microscopy to characterize further the MR-positive terminal endocytic compartment by applying a rapid immunolabeling protocol. NRK cell terminal endocytic compartments were loaded with dextran Alexa (DexA) 647, and the cells then were incubated with the cathepsin B MR substrate for 2 min. After fixation and permeabilization, the cells were immunolabeled with antibodies specific for MPR, LBPA, and rat lysosomal glycoprotein110 (lgp110). There was extensive colocalization of cresyl violet with all three of these markers (Figure 3C), although the fading of the cresyl violet fluorescence after fixation prevented robust quantitative assessment of its colocalization with the antibodies used for immunolabeling. MPR has often been used to distinguish endosomes from lysosomes [18, 19], LBPA has been described as pre-lysosomal [20] and lgp110, like other lysosomal membrane glycoproteins [18] is regarded as a marker of both late endosomes and lysosomes. Using live-cell confocal fluorescence microscopy of HeLa cells stably expressing EGFP-tagged Rab5, Rab7, or Rab9, we observed that MR-positive organelles were negative for the early endosomal marker Rab5, but positive for the late endosomal markers Rab7 and Rab9 (Figure S3E). Taken together, our data showed that the MR-positive organelles demonstrated characteristics of both endosomes and lysosomes, consistent with them being endolysosomes.

Acidotropic Probes Accumulate in Endolysosomes, but Not in Terminal Lysosomes

Late endocytic organelles have been widely shown to accumulate acidotropic probes, which are membrane-permeable weak bases that have helped to define these organelles as having an acidic lumen [18, 21]. As expected, the MR-positive endolysosomes in NRK cells also accumulated the acidotropic probe LysoTracker Green (LG) when the cells were incubated with the cathepsin B MR substrate followed by the LG (Figures 4A, S4A, and S4B). However, if cells were first allowed to accumulate LG followed by incubation with the cathepsin B MR substrate, there was a failure to hydrolyze the substrate and liberate cresyl violet (data not shown), consistent with the accumulation of LG causing alkalinization of the organelle lumen and a sub-optimal pH for cathepsin B activity. In contrast to the endolysosomes, terminal lysosomes, which were DexA positive but MR negative, were unable to accumulate LG (Figures 4A and S4A). Even after incubation with LG for 60 min, large numbers of dextran-loaded, terminal endocytic organelles did not accumulate the acidotropic molecule (Figure S4A). Similar colocalization results were obtained when NRK, HeLa, and MCF7 cells, containing late endocytic compartments pre-loaded with DexA, were compared after incubation with the cathepsin L MR substrate for 2 min and LG for 5 min. These results were consistent with all three cell types having terminal lysosomes that were not acidic and therefore unable to accumulate the acidotropic probe LG (Figures S4C and S4D).

We confirmed the presence of acidic and non-acidic late endocytic compartments by EM, utilizing the fixable cell-permeant, acidotropic reagent DAMP (3-[2,4-dinitroanilino]-3′-amino-N-methyldipropylamine) [22]. NRK cells with terminal endocytic compartments pre-loaded with dextran-Texas red (DexTR) were incubated with 30 μM DAMP for 30 min prior to aldehyde fixation. The cells were processed for immuno-EM using antibodies against Texas red and DAMP. DAMP accumulated in some, but not all, of the late endocytic organelles (Figure 4B). Quantification of DAMP accumulation in the entire DexTR-positive compartment showed a wide range of accumulation consistent with heterogeneity of luminal pH across the organelles of the dextran-loaded late endocytic compartment. In a representative experiment, 22.5% of the DexTR-positive organelles accumulated DAMP to the same extent as nuclei and therefore had a neutral luminal pH (Figure 4C). Further validation of the heterogeneity of luminal pH in dextran-loaded terminal endocytic compartments was obtained using ratiometric imaging after loading with pH-sensitive DexOG and pH-insensitive DexA. In

(C) Quantitation of DAMP immunolabeling in endolysosomes and lysosomes that had accumulated DexTR. The heterogeneity of DAMP accumulation in individual organelles in a representative experiment is shown, with 22.5% of the DexTR-loaded compartments having only accumulated DAMP to the same degree as nuclei (gray points).

(D) Heterogeneity of pH in terminal endocytic compartments determined by ratiometric imaging using DexOG and DexA. Representative experiment is shown in which the pH of 300 organelles was measured.

(E) Cathepsin B activity, determined by incubation with cathepsin B MR substrate, in terminal endocytic compartments with different pH measured by ratiometric imaging (mean ± SEM). Same experiment as in (D).

(F) Confocal fluorescence microscopy images of NRK cells stably expressing TFEB-GFP, pre-loaded with DexA to label terminal endocytic compartments, incubated with cathepsin L MR substrate (MRL) for 3 min and fixed after cytosol washout.

(G) Confocal fluorescence microscopy images of NRK cells stably expressing TFEB-GFP, pre-loaded with DexA, to label terminal endocytic compartments, incubated with 250 nM Torin1 for 3 hr at 37°C, then with cathepsin L MR substrate (MRL) for 2 min before imaging. Graph to the right shows Manders' correlation coefficients (mean ± SEM; three experiments).

Scale bars represent 10 μm (A), 200 nm (B), 10 μm (F), and 5 μm (G). See also Figure S4.

a representative experiment 18% of dextran-loaded organelles had pH values >6.0 (Figure 4D), and few organelles with a pH value >5.5 showed any significant cathepsin B activity (Figure 4E). Overall, our observations were consistent with acid-hydrolase-active endolysosomes being acidic and acid-hydrolase-inactive, dense core, terminal lysosomes being neutral.

Transcription Factor TFEB Localizes to Both Endolysosomes and Terminal Lysosomes

The observation of a range of acidity in late endocytic compartments, including the presence of non-acidic lysosomes, led us to examine the localization of GFP-tagged TFEB (TFEB-GFP) in NRK cells stably expressing this protein. This was because of the reported role of the vacuolar ATPase (V-ATPase) in sensing the physiological and nutritional status of the cell and its upstream function in determining the phosphorylation status of TFEB and whether it traffics to the nucleus to activate transcription [4]. After releasing the cytosolic pool of TFEB-GFP by saponin-permeabilization of the plasma membrane, we observed that membrane-associated TFEB-GFP was present on both MR-positive and -negative terminal endocytic organelles containing fluorescent dextran (Figure 4F). Background fluorescence after plasma membrane permeabilization precluded quantitative analysis, and we therefore also assessed the localization of the TFEB-GFP remaining on endolysosomes and lysosomes after torin treatment. By inhibiting mTORC1, torin prevents the phosphorylation of TFEB causing rapid translocation of the cytosolic pool into the nucleus (see below) but also results in lysosomal accumulation of TFEB that is bound to inactive mTORC1 and no longer able to exchange with the cytosol [4]. Following torin treatment of NRK cells expressing TFEB-GFP, membrane-associated TFEB-GFP was observed on both MR-positive endolysosomes and MR-negative terminal lysosomes, confirmed by the similarity of the Manders' colocalization coefficients for overlap of TFEB-GFP with MR and fluorescent dextran (Figure 4G).

Organelle Kissing Nucleates Activation of the Acid Hydrolases

Given our evidence for a population of dense core, terminal lysosomes, which have a neutral luminal pH and contain immunoreactive but hydrolytically inactive cathepsins, we hypothesized that transient or complete fusion of terminal lysosomes and late endosomes should result in the creation of an appropriate intraorganelle endolysosomal environment to stimulate cathepsin activity. To test this hypothesis, we used a live-cell content mixing assay to investigate whether cathepsin B activity is activated when endosomes exchange content with acid-hydrolase-inactive terminal lysosomes. NRK cell terminal lysosomes were pre-loaded with DexA and late endosomes with DexOG as previously described [6] and the cells observed when incubated with cathepsin B MR substrate in the surrounding medium. We searched for fusion events between DexA-positive, MR-negative lysosomes and DexOG-positive, MR-negative endosomes. These fusion events (see e.g., fusion in Figure S5) were less frequently observed than kissing and fusion between DexA-positive, MR-positive endolysosomes with DexOG-positive, MR-negative endosomes. More often, when searching for interactions between MR-negative endosomes with MR-negative lysosomes, we also observed the close apposition of one or more MR-positive endolysosomes. Nevertheless, the appearance of the lysosomal DexA in a DexOG-positive endosome always commenced before the appearance of cresyl violet fluorescence in the newly forming endolysosome (Figures 5A and 5B; Movie S4). After fusion, we often saw the emergence of tubular budding profiles, containing both fluorescent dextran and cresyl violet, from the resultant endolysosomes (Figure S5; Movie S5), consistent with the tubular budding profiles observed previously after endosome-lysosome kissing and fusion (see, e.g., Figure 1C in [6]. Tubules often retracted back into the endolysosome from which they had emerged, but some broke off, potentially maturing to become re-formed lysosomes (Figure S5).

Cathepsin-Active Endolysosomes and Inactive Terminal Lysosomes Exist in Dynamic Equilibrium

We have previously shown in NRK cells that the swelling of terminal endocytic organelles following uptake of undigestible sucrose to form sucrosomes is explained by the swelling of compartments, which can be morphologically defined as endolysosomes and are able to recruit all of the terminal dense core lysosomes over a period of several hours [9]. Subsequent endocytic uptake of invertase results in collapse and disappearance of the sucrosomes and re-formation of dense core lysosomes, such that the normal equilibrium of endolysosomes and dense core lysosomes is re-established (see Figure 8 in [9]). In the present study, using NRK cells in which terminal endocytic compartments were pre-loaded with DexA, we observed that, following incubation with sucrose, the sucrosomes were MR-positive and could accumulate LG (Figures 6A, S6A, and S6B), confirming that they were swollen endolysosomes. Subsequent fluid phase endocytic uptake of invertase resulted in MR-positive tubules emanating from these endolysosomes, some of which broke off (Figures 6B, S6C, and S6D; Movie S6). The sucrosomes were AcPase positive when examined by TEM (Figure 6C) as were the tubules emanating from sucrosomes after invertase treatment (Figures 6C and 6D).

In other cell types, sucrose treatment has been shown to stimulate autophagy in a time- and sucrose concentration-dependent manner, causing an accumulation of autophagosomes [23] and also to result in the nuclear translocation of the lysosome-associated transcription factor TFEB (transcription factor EB) ([2] and our unpublished data on MCF7 cells). If that were also the case in NRK cells, it may have complicated the interpretation of our sucrosome experiments. However, under the conditions of our experiments, treatment of NRK cells with 30 mM sucrose for 24 hr caused no significant accumulation of LC3-positive autophagosomes (Figure 6E), and, in an NRK cell line stably expressing TFEB-GFP, there was no significant translocation of TFEB to the nucleus (Figure 6F).

DISCUSSION

Our data show that in mammalian cells endolysosomes are the principal sites of intracellular acid hydrolase activity and can be distinguished from acid-hydrolase-inactive terminal lysosomes, which act as a store of acid hydrolases. We have shown in three widely studied mammalian cell lines that endolysosomes, but not lysosomes, contain active cathepsins B, C, and

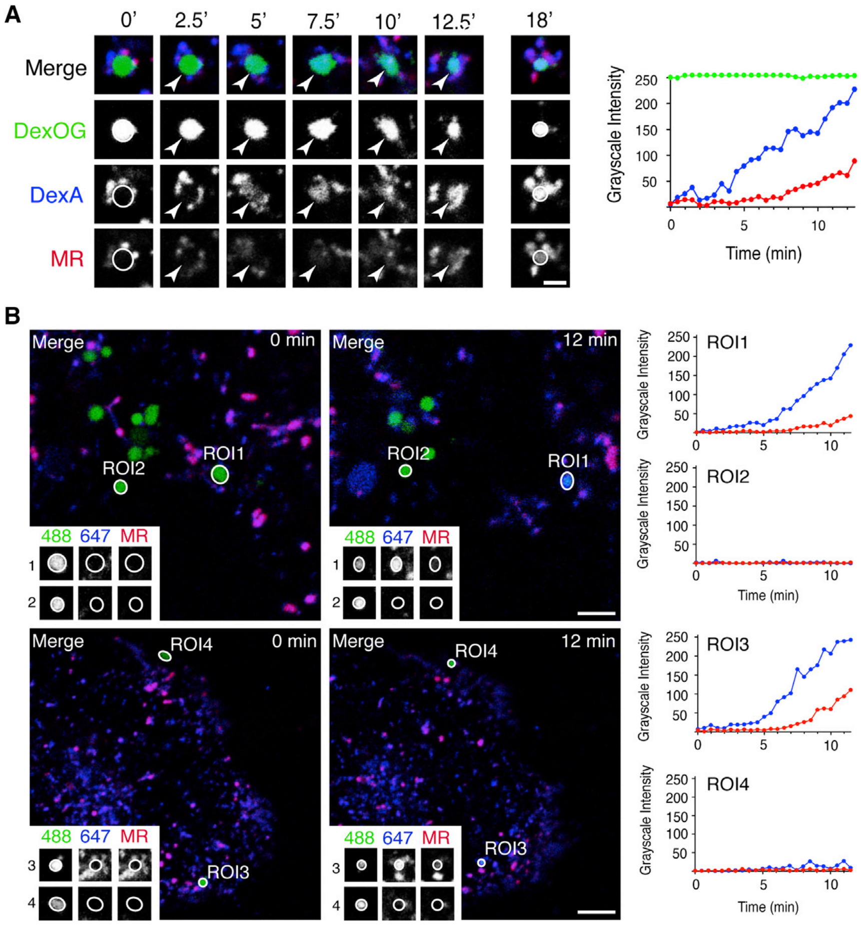

Figure 5. Organelle Kissing Causes Cathepsin Activation in the Newly Forming Endolysosome

Terminal endocytic compartments of NRK cells were pre-loaded with DexA for 4 hr followed by a 20-hr chase in DexA-free medium, and then late endosomes were loaded with DexOG by uptake for 10 min followed by a 5 min chase in DexOG-free medium containing cathepsin B MR substrate.

(A) Time-lapse confocal microscopy of the living cells for 12.5 min, showing that transient fusion of a DexOG-positive late endosome with DexA-laden terminal endocytic compartments resulted in the gradual acquisition of DexA (blue) in the DexOG-positive organelle (green) and a subsequent rise in cresyl violet fluorescence from the cleaved MR substrate (red), also demonstrated (right) by the mean grayscale intensity profile of each fluorochrome in the identified organelle.

(B) Further examples of organelles tracked after the addition of cathepsin B MR substrate. The graphs to the right show that DexOG-laden late endosomes (green, 488), which acquired DexA (blue, 647) after transient fusions with terminal endocytic organelles both exhibited a subsequent rise in cresyl violet fluorescence liberated from cleaved MR substrate (red; region of interest 1 [ROI1] and ROI3), but organelles that did not acquire DexA from terminal endocytic compartments during the time course also failed to acquire cresyl violet fluorescence (ROI2 and ROI4).

Scale bars represent 10 μm. See also Figure S5.

L, as well as AcPase. We observed that the acid-hydrolase-active endolysosomes and inactive terminal lysosomes are in dynamic equilibrium, linked in a lysosome regeneration cycle (Figure 7), consistent with previous studies showing the transient and complete fusion of late endosomes/endolysosomes with terminal lysosomes as well as the process of lysosome re-formation [6, 9, 24]. Our present data, based on the localization of hydrolase activity, show that a higher proportion of organelles in the lysosome regeneration cycle are endolysosomes compared with previous estimates based on morphological or marker localization criteria [9, 25]. The concept that endocytosed macromolecules are delivered to lysosomal enzymes through the formation of acid-hydrolase-active endolysosomes, as a result of transient and complete fusion events, is very different to such compartments simply being intermediates in a linear transport route between endosomes and inactive terminal storage lysosomes, with or without recycling pathways [26, 27]. The live-cell experiments, in which we imaged and recorded individual organelles, showed that fusion events, resulting in the formation of endolysosomes, preceded the onset of acid hydrolase activity. These experiments were not designed to provide detailed information about the time course and kinetics of delivery of endocytosed ligands from the cell surface to acid hydrolases, because we did not use an endocytic pulse, such as could be achieved with a receptor-binding ligand. Although ours is the first study formally demonstrating acid hydrolase activity in the endolysosomal hybrid organelle, the data obtained are consistent with previous observations of acid hydrolase and protein degradation activity in intracellular compartments with endosomal characteristics [18, 28, 29]. We also note, in particular, that the endolysosomal compartment that we have defined in NRK cells has many of the characteristics of the pre-lysosomal compartment previously described in the same cell type [18, 19].

A key feature of the hydrolase-inactive terminal lysosomes observed in the present study is that not only do they contain inactive acid hydrolases, but they can all be recruited to the endolysosome compartment and are re-formed from it. Thus, they are not inert residual bodies or post-lysosomes, nor simply a storage or accumulation compartment for slowly and/or nondegradable material [30]. In contrast, they appear to be readily usable storage organelles for mature acid hydrolases of a type previously proposed [31, 32], with the endolysosome representing the cell's stomach for digestion of endocytosed macromolecules. The observation that terminal storage lysosomes do not accumulate acidotropic reagents implies that these compartments are not acidic. The existence of non-acidic subsets of lysosomes has been reported previously, leading to the proposal that lysosomal pH fluctuates to allow different degradative processes to occur in sequence with some being mediated by lysosomal enzymes that are inactive in the acidic pH range [33]. In addition, a non-acidic post-lysosomal compartment that is cathepsin D negative by immunostaining depends on

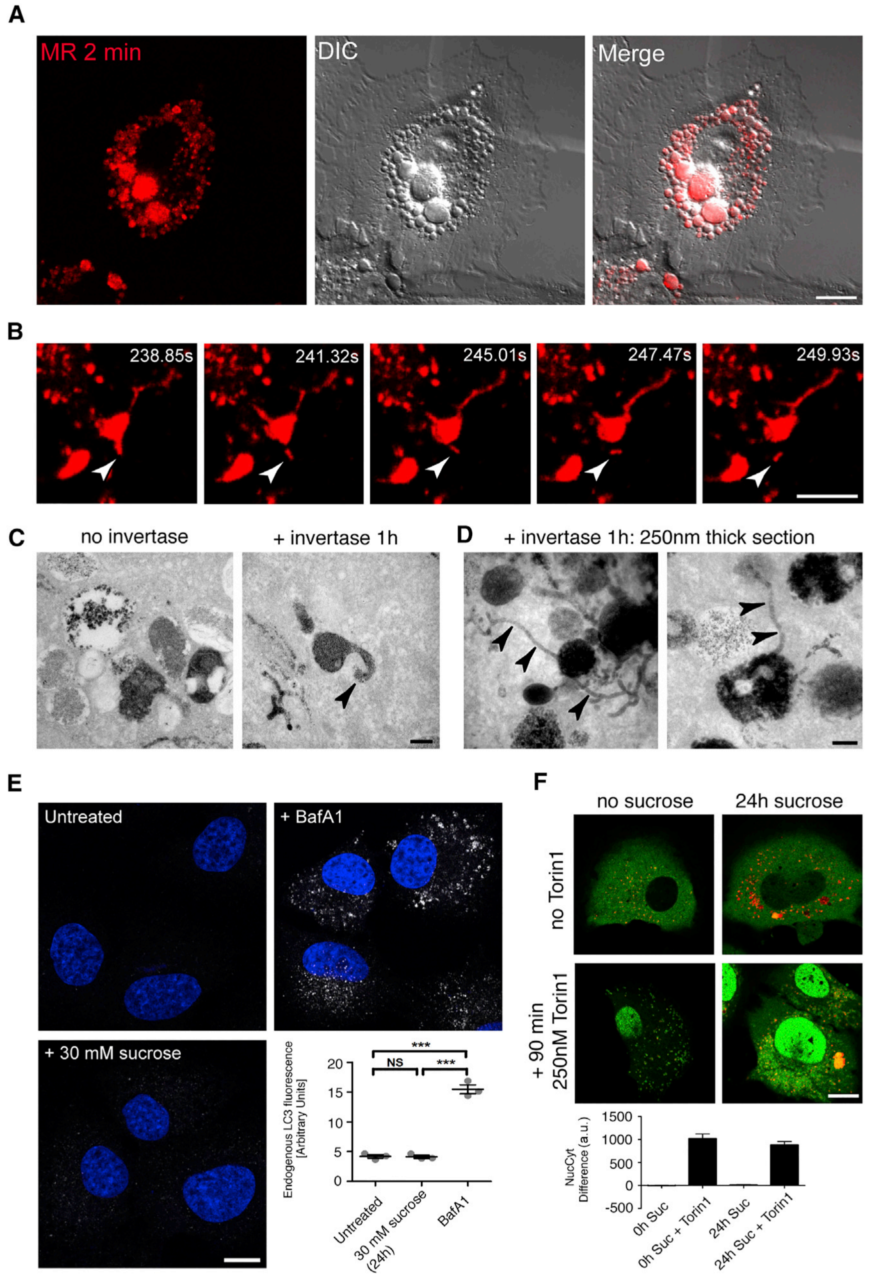

Figure 6. Sucrosomes Are Swollen Endolysosomes from which Lysosomes Can Be Re-formed

Sucrosomes were formed in NRK cells by endocytosis of 30 mM sucrose for 24 hr.

(A) Incubation of the cells with cathepsin B MR substrate for 2 min showed that the swollen vacuoles seen by differential interference contrast (DIC) microscopy were cathepsin active (MR).

(B) NRK cells containing sucrosomes were incubated with medium containing 0.5 mg/ml invertase for 1 hr followed by the addition of cathepsin B MR substrate for 2 min. A time-lapse series of images collected on the confocal microscope showed that addition of invertase resulted in extensive tubulation of the cathepsin-active sucrosomes and in some instances tubules could be observed detaching from the parent sucrosome (arrowhead). See also Movie S6.

(C) Following incubation with invertase as in (B), NRK cells containing sucrosomes were fixed for TEM and AcPase cytochemistry. Consistent with cleavage of the cathepsin B MR substrate, cerium phosphate deposition was seen in sucrosomes and tubules (arrowhead).

(D) When 250-nm-thick sections were prepared from the cells shown in (C), the extent of cathepsin-active tubules (arrowheads) emanating from sucrosomes was more clearly revealed.

(E) Anti-LC3 immunoreactivity was used as a marker of autophagosome accumulation in NRK cells following sucrosome formation (400 nM bafilomycin A1 [BafA1] for 8 hr was a control for autophagosome accumulation). Nuclei were identified by Hoechst staining and mean LC3 immunoreactivity intensity quantified (mean ± SEM; three experiments), following confocal microscopy. ***$p < 0.001$ (Student's t test).

(F) NRK cells stably expressing TFEB-GFP (green) were incubated with or without 30 mM sucrose (Suc) for 24 hr and subsequently with or without 250 nM Torin 1 for 90 min (as a control for TFEB-GFP translocation to the nucleus), before addition of cathepsin B MR substrate to show cathepsin-active organelles (red). The NucCyt difference plotted (mean ± SEM; three experiments) is the mean fluorescence intensity in the nuclei (Nuc) after subtracting the mean intensity of pixels in the cytoplasm (Cyt) in a.u.

Scale bars represent 10 μm (A and B), 200 nm (C and D), and 10 μm (E and F). See also Figure S6.

the accumulation of non-digestible material for its formation and is made up of inert residual bodies has been described [34]. In contrast to this latter report, the hydrolase-inactive terminal storage lysosomes that we observed in NRK and MCF7 cells do contain cathepsins by both the criteria of activation of hydrolase activity when the intra-organelle pH is equilibrated to pH 5 with ionophores and immuno-EM. Our data are consistent with a recent report from Johnson et al. [35] that several cell types have a subpopulation of lysosomes with pH values >6. However, in contrast to our proposal that the pH of endolysosomes and lysosomes is linked to their position in the lysosome regeneration cycle, Johnson et al. suggested that it is a consequence of their subcellular location, with more peripheral lysosomes being less acidic. Johnston et al. also found that less acidic, peripheral lysosomes have more limited access to material exported by the biosynthetic pathway, but the existence of inactive cathepsins in terminal lysosomes in our experiments implies that the major reason for loss of acid hydrolase activity in these lysosomes is the increase in pH.

Several mechanisms may be functioning together to result in terminal lysosomes having a higher pH than endolysosomes, although the relative importance of each is not yet clear. The accumulation of acidotropic molecules could itself be affected by other factors including the presence of transporters in the limiting membrane and the available aqueous volume, which will alter as a result of content condensation occurring during

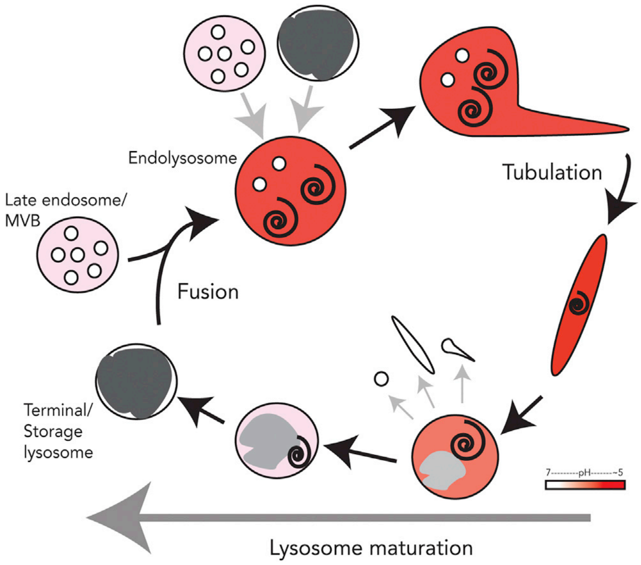

Figure 7. The Lysosome Regeneration Cycle

Scheme showing fusion of terminal storage lysosomes with late endosomes to form endolysosomes and the subsequent tubulation, maturation, and content condensation processes required for the re-formation of terminal storage lysosomes. Endolysosomes can undertake further fusions with late endosomes and/or terminal storage lysosomes. Acidic compartments are shaded pink (slightly acidic) to dark red (pH ~5). Electron-dense content is shaded dark gray.

lysosome re-formation [9, 36]. Johnson et al. [35] investigated why some lysosomes are more alkaline than others by measuring buffering capacity as well as using assays of passive H^+ permeability and lysosome reacidification, in the presence and absence of a V-ATPase inhibitor, after lysosomal transmembrane pH gradient dissipation with a protonophore. These assays provided evidence that increased passive (leak) permeability to protons together with reduced V-ATPase activity accounts for the reduced acidifying ability of some lysosomes. In this context, it is interesting to note the removal of V-ATPase to generate a neutral post-lysosomal compartment in *Dictyostelium discoideum* [37], the reversible dissociation/assembly of the V-ATPase V0V1 complex to regulate ATP-dependent proton transport [38] and the evidence that modulation of the activity of the Rab7 effector RILP (Rab-interacting lysosomal protein) may be used to control V-ATPase function [39].

The re-formation of terminal lysosomes from endolysosomes is not well understood, although previous studies of lysosome re-formation in cultured cells and in cell-free experiments have suggested that it requires budding and tubulation events at the surface of the endolysosome and is a maturation process that includes content condensation [9, 36, 40, 41]. Interestingly, re-formation of lysosomes from autolysosomes, which result from autophagosome-lysosome fusion [42], has been previously shown to occur via lysosomal membrane glycoprotein-positive tubules [43–47], just as we observed when studying re-formation of lysosomes after invertase uptake into cells containing sucrosomes (swollen endolysosomes). We did not extend our sucrosome/invertase experiments to the other cell lines that we studied because we observed that generating sucrosomes in HeLa and MCF7 cells, while possible (see also [48] for HeLa cells), was not as straightforward as in NRK cells. For these cells types, it may be preferable to use a different non-hydrolysable disaccharide or other indigestible substance [49, 50]. Consistent with our data on lysosome re-formation from sucrosomes, a recent study of tubulation of late endocytic compartments in macrophages showed that tubules forming from a parent endolysosomal compartment, and considered to be nascent lysosomes, contained the same luminal fluorescent marker as the parent compartment [40]. The fact that lysosome re-formation from endolysosomes is a maturation process implies that the endolysosomal compartment will be heterogeneous since each organelle will be at a different stage in the formation/re-formation cycle. This is consistent with the heterogeneous ultrastructural morphology seen in the present study and with the wide range of pH in individual endolysosomes observed by DAMP labeling or ratiometric imaging. It also means that simply investigating whether a marker such as MPR or a specific Rab is present may not be a sufficient means of determining intermediate stages in the cycle. At present we also do not know the fusogenic capability of tubules breaking off from endolysosomes or how this alters during the maturation process leading to lysosome re-formation.

The availability of a pool of inactive acid hydrolases in terminal storage lysosomes could allow the cell to respond to altered extracellular conditions or cellular stress by increasing the rate at which terminal lysosomes fuse with endosomes, autophagosomes or phagosomes and thus increase degradative activity. Maintaining part of the cell's complement of acid hydrolases in an environment where they are inactive may also increase the protection of both limiting membrane and luminal contents, thereby enhancing lysosome longevity. At present, we do not know whether there are nutritional or signaling conditions under which the ratio of acid-hydrolase-active endolysosomes to inactive lysosomes would alter, nor do we fully understand the relationship of endolysosomes to autolysosomes, although the two may be synonymous depending on nutritional state. These are likely to be fruitful areas of further study, not least because of the increasingly recognized importance of the cytoplasmic surface of the limiting membranes of endolysosomes/lysosomes as the site of action of signaling complexes that regulate cellular metabolism (reviewed in [3]).

EXPERIMENTAL PROCEDURES

Further details on experimental procedures (e.g., reagents, antibodies, plasmids, and microscopy) are provided in the Supplemental Experimental Procedures.

Endocytic Uptake of Fluorescent Dextrans and DQ-BSA

Terminal endocytic compartments in cultured NRK fibroblasts, HeLa-M epithelial cells, or MCF7 human breast adenocarcinoma cells were loaded with 0.5 mg/ml lysine-fixable dextran-OG 488, dextran-Texas red, or dextran-Alexa 647, all from Thermo Fisher Scientific in culture medium for 4 hr at 37°C followed by incubation in conjugate-free medium for 20 hr as previously described [9]. For labeling of proteolytically active endocytic compartments, cells were incubated with 0.1 mg/ml DQ Green BSA from Thermo Fisher Scientific for 15–30 min at 37°C in culture medium.

Incubation with Cathepsin Substrates

To label endocytic organelles in which cathepsin B or cathepsin L was catalytically active, cells were incubated with the respective Magic Red substrates from ImmunoChemistry Technologies, at a final concentration of 673 nM.

Glycyl-L-phenylalanine 2-naphthylamide (GPN) from Santa Cruz Biotechnology was used at a final concentration of 200 μM.

Accumulation of Acidotropic Compounds

For labeling of acidic organelles for fluorescence microscopy, cells were incubated for 5 min at 37°C with 50 nM LysoTracker Green from Thermo Fisher Scientific. For TEM, cells were incubated for 30 min at 37°C with 30 μM DAMP, before aldehyde fixation, processing for TEM and immunolabeling with anti-dinitrophenol (DNP) antibodies.

Microscopy

Preparation, fixation, and labeling of cells for microscopy, acid phosphatase cytochemistry, preparation of sections for TEM, CLEM, immuno-EM, use of microscopes, image analysis, and quantification were carried out as described in the Supplemental Experimental Procedures. Confocal and live-cell microscopy was carried out on Zeiss LSM710, 780, or 880 confocal microscopes. EM sections were examined with an FEI Tecnai G2 Spirit BioTwin transmission electron microscope. For quantification of fluorescence microscopy, Pearson's correlation coefficients and Manders' colocalization coefficients were used, respectively, to measure the overall association of two fluorescent probes and the fraction of the total fluorescence of one probe overlapping with a second as described in the Supplemental Experimental Procedures. In the figure legends, Manders' coefficients are presented such that M1, DexOG:MR, indicates the fraction of DexOG-positive pixels containing cresyl violet after hydrolysis of a MR substrate and M2, MR:DexOG, indicates the fraction of MR-positive pixels containing DexOG, likewise for other Manders' coefficients presented. Quantified data are presented as mean ± SEM (number of experiments with $\geq$17 cells per experiment, unless otherwise stated). Quantitation of LC3 fluorescence intensity to assess autophagy is presented as mean ± SEM (number of experiments with >100 cells per condition in each experiment). Nuclear translocation of TFEB-GFP was assessed with a Cellomics ArrayScan VTi high content screening microscope (Thermo Fisher Scientific), and data are presented as mean ± SEM (number of experiments with 750 cells per condition in each experiment).

SUPPLEMENTAL INFORMATION

Supplemental Information includes Supplemental Experimental Procedures, six figures, and six movies and can be found with this article online at http://dx.doi.org/10.1016/j.cub.2016.06.046.

AUTHOR CONTRIBUTIONS

N.A.B. and J.P.L. conceived and directed the study. N.A.B. and L.J.D. performed all the confocal microscopy experiments including quantification, with N.A.B. additionally undertaking all the EM studies and L.J.D. preparing and studying cells stably expressing TFEB-GFP. All the authors discussed the results, interpreted data, and co-wrote the paper.

CONFLICTS OF INTEREST

L.J.D. is supported by a BBSRC industrial CASE studentship with GSK Research and Development.

ACKNOWLEDGMENTS

This work was supported by MRC research grant MR/M010007/1. The CIMR is supported by Wellcome Trust Strategic Award 100140. The Cellomics ArrayScan VTi High Content Screening Microscope, Zeiss LSM710 confocal microscope, and FEI Tecnai G2 Spirit BioTWIN transmission EM were purchased with Wellcome Trust grants 079919 and 093026. We thank Sally Gray for preparing and sequencing pLXIN constructs and Matthew Gratian for help with light microscopy and analytical software.

Received: March 10, 2016
Revised: June 21, 2016
Accepted: June 21, 2016
Published: August 4, 2016

REFERENCES

1. de Duve, C. (2005). The lysosome turns fifty. Nat. Cell Biol. *7*, 847–849.
2. Sardiello, M., Palmieri, M., di Ronza, A., Medina, D.L., Valenza, M., Gennarino, V.A., Di Malta, C., Donaudy, F., Embrione, V., Polishchuk, R.S., et al. (2009). A gene network regulating lysosomal biogenesis and function. Science *325*, 473–477.
3. Settembre, C., Fraldi, A., Medina, D.L., and Ballabio, A. (2013). Signals from the lysosome: a control centre for cellular clearance and energy metabolism. Nat. Rev. Mol. Cell Biol. *14*, 283–296.
4. Settembre, C., Zoncu, R., Medina, D.L., Vetrini, F., Erdin, S., Erdin, S., Huynh, T., Ferron, M., Karsenty, G., Vellard, M.C., et al. (2012). A lysosome-to-nucleus signalling mechanism senses and regulates the lysosome via mTOR and TFEB. EMBO J. *31*, 1095–1108.
5. Roczniak-Ferguson, A., Petit, C.S., Froehlich, F., Qian, S., Ky, J., Angarola, B., Walther, T.C., and Ferguson, S.M. (2012). The transcription factor TFEB links mTORC1 signaling to transcriptional control of lysosome homeostasis. Sci. Signal. *5*, ra42.
6. Bright, N.A., Gratian, M.J., and Luzio, J.P. (2005). Endocytic delivery to lysosomes mediated by concurrent fusion and kissing events in living cells. Curr. Biol. *15*, 360–365.
7. Luzio, J.P., Pryor, P.R., and Bright, N.A. (2007). Lysosomes: fusion and function. Nat. Rev. Mol. Cell Biol. *8*, 622–632.
8. Huotari, J., and Helenius, A. (2011). Endosome maturation. EMBO J. *30*, 3481–3500.
9. Bright, N.A., Reaves, B.J., Mullock, B.M., and Luzio, J.P. (1997). Dense core lysosomes can fuse with late endosomes and are re-formed from the resultant hybrid organelles. J. Cell Sci. *110*, 2027–2040.
10. Creasy, B.M., Hartmann, C.B., White, F.K., and McCoy, K.L. (2007). New assay using fluorogenic substrates and immunofluorescence staining to measure cysteine cathepsin activity in live cell subpopulations. Cytometry A *71*, 114–123.
11. Pols, M.S., ten Brink, C., Gosavi, P., Oorschot, V., and Klumperman, J. (2013). The HOPS proteins hVps41 and hVps39 are required for homotypic and heterotypic late endosome fusion. Traffic *14*, 219–232.
12. Jadot, M., Colmant, C., Wattiaux-De Coninck, S., and Wattiaux, R. (1984). Intralysosomal hydrolysis of glycyl-L-phenylalanine 2-naphthylamide. Biochem. J. *219*, 965–970.
13. Haller, T., Dietl, P., Deetjen, P., and Völkl, H. (1996). The lysosomal compartment as intracellular calcium store in MDCK cells: a possible involvement in InsP3-mediated Ca2+ release. Cell Calcium *19*, 157–165.
14. Steinberg, B.E., Huynh, K.K., Brodovitch, A., Jabs, S., Stauber, T., Jentsch, T.J., and Grinstein, S. (2010). A cation counterflux supports lysosomal acidification. J. Cell Biol. *189*, 1171–1186.
15. López-Sanjurjo, C.I., Tovey, S.C., Prole, D.L., and Taylor, C.W. (2013). Lysosomes shape Ins(1,4,5)P3-evoked Ca2+ signals by selectively sequestering Ca2+ released from the endoplasmic reticulum. J. Cell Sci. *126*, 289–300.
16. Penny, C.J., Kilpatrick, B.S., Han, J.M., Sneyd, J., and Patel, S. (2014). A computational model of lysosome-ER Ca2+ microdomains. J. Cell Sci. *127*, 2934–2943.
17. Vázquez, C.L., and Colombo, M.I. (2009). Assays to assess autophagy induction and fusion of autophagic vacuoles with a degradative compartment, using monodansylcadaverine (MDC) and DQ-BSA. Methods Enzymol. *452*, 85–95.
18. Griffiths, G., Hoflack, B., Simons, K., Mellman, I., and Kornfeld, S. (1988). The mannose 6-phosphate receptor and the biogenesis of lysosomes. Cell *52*, 329–341.
19. Griffiths, G., Matteoni, R., Back, R., and Hoflack, B. (1990). Characterization of the cation-independent mannose 6-phosphate receptor-enriched prelysosomal compartment in NRK cells. J. Cell Sci. *95*, 441–461.

20. Kobayashi, T., Stang, E., Fang, K.S., de Moerloose, P., Parton, R.G., and Gruenberg, J. (1998). A lipid associated with the antiphospholipid syndrome regulates endosome structure and function. Nature *392*, 193–197.
21. Ohkuma, S., and Poole, B. (1981). Cytoplasmic vacuolation of mouse peritoneal macrophages and the uptake into lysosomes of weakly basic substances. J. Cell Biol. *90*, 656–664.
22. Anderson, R.G., Falck, J.R., Goldstein, J.L., and Brown, M.S. (1984). Visualization of acidic organelles in intact cells by electron microscopy. Proc. Natl. Acad. Sci. USA *81*, 4838–4842.
23. Higuchi, T., Nishikawa, J., and Inoue, H. (2015). Sucrose induces vesicle accumulation and autophagy. J. Cell. Biochem. *116*, 609–617.
24. Gan, Z., Ram, S., Vaccaro, C., Ober, R.J., and Ward, E.S. (2009). Analyses of the recycling receptor, FcRn, in live cells reveal novel pathways for lysosomal delivery. Traffic *10*, 600–614.
25. Kaufmann, A.M., and Krise, J.P. (2008). Niemann-Pick C1 functions in regulating lysosomal amine content. J. Biol. Chem. *283*, 24584–24593.
26. Berg, T., Gjøen, T., and Bakke, O. (1995). Physiological functions of endosomal proteolysis. Biochem. J. *307*, 313–326.
27. Humphries, W.H., 4th, Szymanski, C.J., and Payne, C.K. (2011). Endo-lysosomal vesicles positive for Rab7 and LAMP1 are terminal vesicles for the transport of dextran. PLoS ONE *6*, e26626.
28. Casciola-Rosen, L.A., Renfrew, C.A., and Hubbard, A.L. (1992). Lumenal labeling of rat hepatocyte endocytic compartments. Distribution of several acid hydrolases and membrane receptors. J. Biol. Chem. *267*, 11856–11864.
29. Galmes, R., ten Brink, C., Oorschot, V., Veenendaal, T., Jonker, C., van der Sluijs, P., and Klumperman, J. (2015). Vps33B is required for delivery of endocytosed cargo to lysosomes. Traffic *16*, 1288–1305.
30. Hellevik, T., Martinez, I., Olsen, R., Toh, B.H., Webster, P., and Smedsrød, B. (1998). Transport of residual endocytosed products into terminal lysosomes occurs slowly in rat liver endothelial cells. Hepatology *28*, 1378–1389.
31. Griffiths, G. (1996). On vesicles and membrane compartments. Protoplasma *195*, 37–58.
32. Tjelle, T.E., Brech, A., Juvet, L.K., Griffiths, G., and Berg, T. (1996). Isolation and characterization of early endosomes, late endosomes and terminal lysosomes: their role in protein degradation. J. Cell Sci. *109*, 2905–2914.
33. Butor, C., Griffiths, G., Aronson, N.N., Jr., and Varki, A. (1995). Co-localization of hydrolytic enzymes with widely disparate pH optima: implications for the regulation of lysosomal pH. J. Cell Sci. *108*, 2213–2219.
34. Hirota, Y., Masuyama, N., Kuronita, T., Fujita, H., Himeno, M., and Tanaka, Y. (2004). Analysis of post-lysosomal compartments. Biochem. Biophys. Res. Commun. *314*, 306–312.
35. Johnson, D.E., Ostrowski, P., Jaumouillé, V., and Grinstein, S. (2016). The position of lysosomes within the cell determines their luminal pH. J. Cell Biol. *212*, 677–692.
36. Pryor, P.R., Mullock, B.M., Bright, N.A., Gray, S.R., and Luzio, J.P. (2000). The role of intraorganellar Ca(2+) in late endosome-lysosome heterotypic fusion and in the reformation of lysosomes from hybrid organelles. J. Cell Biol. *149*, 1053–1062.
37. Carnell, M., Zech, T., Calaminus, S.D., Ura, S., Hagedorn, M., Johnston, S.A., May, R.C., Soldati, T., Machesky, L.M., and Insall, R.H. (2011). Actin polymerization driven by WASH causes V-ATPase retrieval and vesicle neutralization before exocytosis. J. Cell Biol. *193*, 831–839.
38. Stransky, L.A., and Forgac, M. (2015). Amino Acid Availability Modulates Vacuolar H+-ATPase Assembly. J. Biol. Chem. *290*, 27360–27369.
39. De Luca, M., Cogli, L., Progida, C., Nisi, V., Pascolutti, R., Sigismund, S., Di Fiore, P.P., and Bucci, C. (2014). RILP regulates vacuolar ATPase through interaction with the V1G1 subunit. J. Cell Sci. *127*, 2697–2708.
40. Miller, A., Schafer, J., Upchurch, C., Spooner, E., Huynh, J., Hernandez, S., McLaughlin, B., Oden, L., and Fares, H. (2015). Mucolipidosis type IV protein TRPML1-dependent lysosome formation. Traffic *16*, 284–297.
41. Mousavi, S.A., Brech, A., Berg, T., and Kjeken, R. (2003). Phosphoinositide 3-kinase regulates maturation of lysosomes in rat hepatocytes. Biochem. J. *372*, 861–869.
42. Jahreiss, L., Menzies, F.M., and Rubinsztein, D.C. (2008). The itinerary of autophagosomes: from peripheral formation to kiss-and-run fusion with lysosomes. Traffic *9*, 574–587.
43. Yu, L., McPhee, C.K., Zheng, L., Mardones, G.A., Rong, Y., Peng, J., Mi, N., Zhao, Y., Liu, Z., Wan, F., et al. (2010). Termination of autophagy and reformation of lysosomes regulated by mTOR. Nature *465*, 942–946.
44. Rong, Y., Liu, M., Ma, L., Du, W., Zhang, H., Tian, Y., Cao, Z., Li, Y., Ren, H., Zhang, C., et al. (2012). Clathrin and phosphatidylinositol-4,5-bisphosphate regulate autophagic lysosome reformation. Nat. Cell Biol. *14*, 924–934.
45. Chen, Y., and Yu, L. (2013). Autophagic lysosome reformation. Exp. Cell Res. *319*, 142–146.
46. Chang, J., Lee, S., and Blackstone, C. (2014). Spastic paraplegia proteins spastizin and spatacsin mediate autophagic lysosome reformation. J. Clin. Invest. *124*, 5249–5262.
47. Schulze, R.J., Weller, S.G., Schroeder, B., Krueger, E.W., Chi, S., Casey, C.A., and McNiven, M.A. (2013). Lipid droplet breakdown requires dynamin 2 for vesiculation of autolysosomal tubules in hepatocytes. J. Cell Biol. *203*, 315–326.
48. Bandyopadhyay, D., Cyphersmith, A., Zapata, J.A., Kim, Y.J., and Payne, C.K. (2014). Lysosome transport as a function of lysosome diameter. PLoS ONE *9*, e86847.
49. Cohn, Z.A., and Ehrenreich, B.A. (1969). The uptake, storage, and intracellular hydrolysis of carbohydrates by macrophages. J. Exp. Med. *129*, 201–225.
50. Montgomery, R.R., Webster, P., and Mellman, I. (1991). Accumulation of indigestible substances reduces fusion competence of macrophage lysosomes. J. Immunol. *147*, 3087–3095.

Current Biology, Volume *26*

Supplemental Information

Endolysosomes Are the Principal Intracellular Sites of Acid Hydrolase Activity

Nicholas A. Bright, Luther J. Davis, and J. Paul Luzio

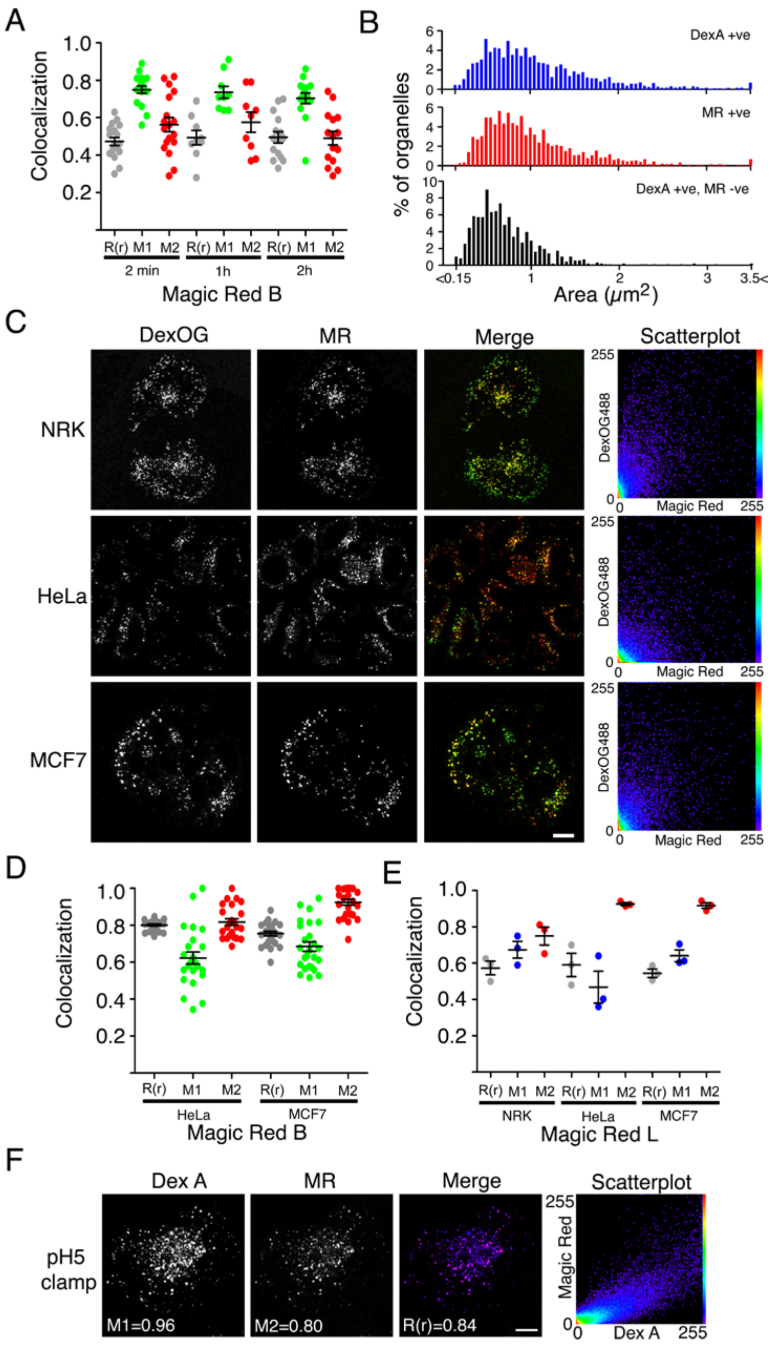

Figure S1: Endolysosomes are the principal sites of cathepsin B and cathepsin L catalytic activity. Related to Figure 1. Terminal endocytic compartments were loaded with fluorescent dextran (DexOG or DexA) as for Figure 1. **A**) Pearson's (R(r); gray) and Mander's (M1, green, DexOG:MR and M2, red, MR:DexOG) correlation coefficients (mean ± SEM within a single experiment), for colocalization of pre-loaded DexOG and hydrolysed (different times) cathepsin B MR substrate in NRK cells (all data from the same experiment shown in Figure 1B). **B)** Size distribution of all DexA-positive; MR-positive; MR-negative, DexA-positive organelles in NRK cells (single experiment). **C**) Cathepsin-active terminal endocytic organelles in NRK, HeLa and MCF7 cells revealed by incubation with 1 h hydrolysed cathepsin B MR substrate. **D**) Pearson's (R(r), gray) and Manders' (M1, green, DexOG:MR and M2, red, MR:DexOG) correlation coefficients for colocalization of pre-loaded DexOG and 1 h hydrolysed cathepsin B MR substrate in HeLa or MCF7 cells (mean ± SEM within a single experiment). **E**) Pearson's (R(r); gray) and Mander's (M1, blue, DexA:MR and M2, red,MR:DexA) correlation coefficients for colocalization of pre-loaded DexA and 1 h hydrolysed cathepsin L MR substrate in NRK, HeLa and MCF7 cells (mean ± SEM (3)). **F)** Cathepsin B MR substrate access to all terminal endocytic organelles in a NRK cell in which the culture medium was replaced with a pH 5-buffered medium at 37°C containing the cathepsin B MR substrate, 10 μM monensin and 10 μM nigericin. The representative image taken after 5 min on the confocal microscope shows the coincidence of DexA-positive and MR-positive organelles (Pearson's R(r) and Manders' M1, DexA:MR and M2, MR:DexA values given for cells shown), and a scatterplot of the merged image reveals the coincidence of the two fluorochromes. Scale Bars: C) 10 μm, F) 5 μm.

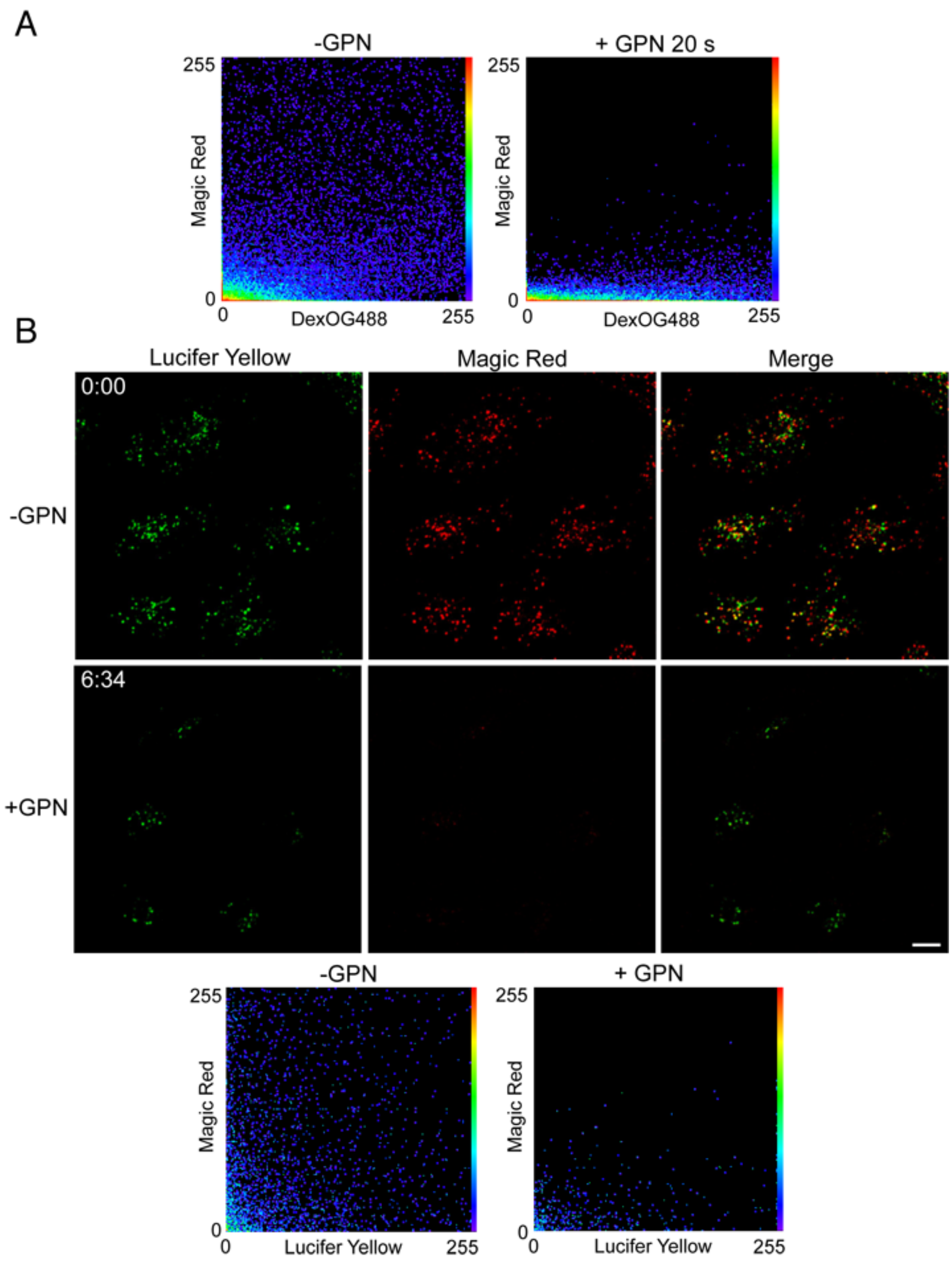

Figure S2: Magic Red-positive endolysosomes contain active cathepsin C. Related to Figure 2.
A) Scatterplots of the pixel intensities of the merged images shown in Figure 2A, taken from a timelapse confocal microscopy series before, or 20 s after addition of 200 μM GPN, revealing rapid dissipation of cresyl violet (Magic Red). **B)** Terminal endocytic compartments of NRK cells were loaded with Lucifer Yellow for 4 h followed by a 20 h chase in Lucifer Yellow-free medium and the cells then incubated for 2 min with cathepsin B MR substrate. Timelapse images of the living cells were collected on the confocal microscope. Addition of 200 μM GPN resulted in rapid dissipation of cresyl violet (Magic Red) from cathepsin-active endolysosomes but the Lucifer Yellow (Mr 457) was retained in a population of the terminal endocytic organelles revealing that it also resided in organelles that were not catalytically active for cathepsin C. Scatterplots of the pixel intensities of the images taken before and after the addition of GPN clearly show the dissipation of cresyl violet (Magic Red) from the cells but the retention of Lucifer Yellow. Scale Bar: 10 μm.

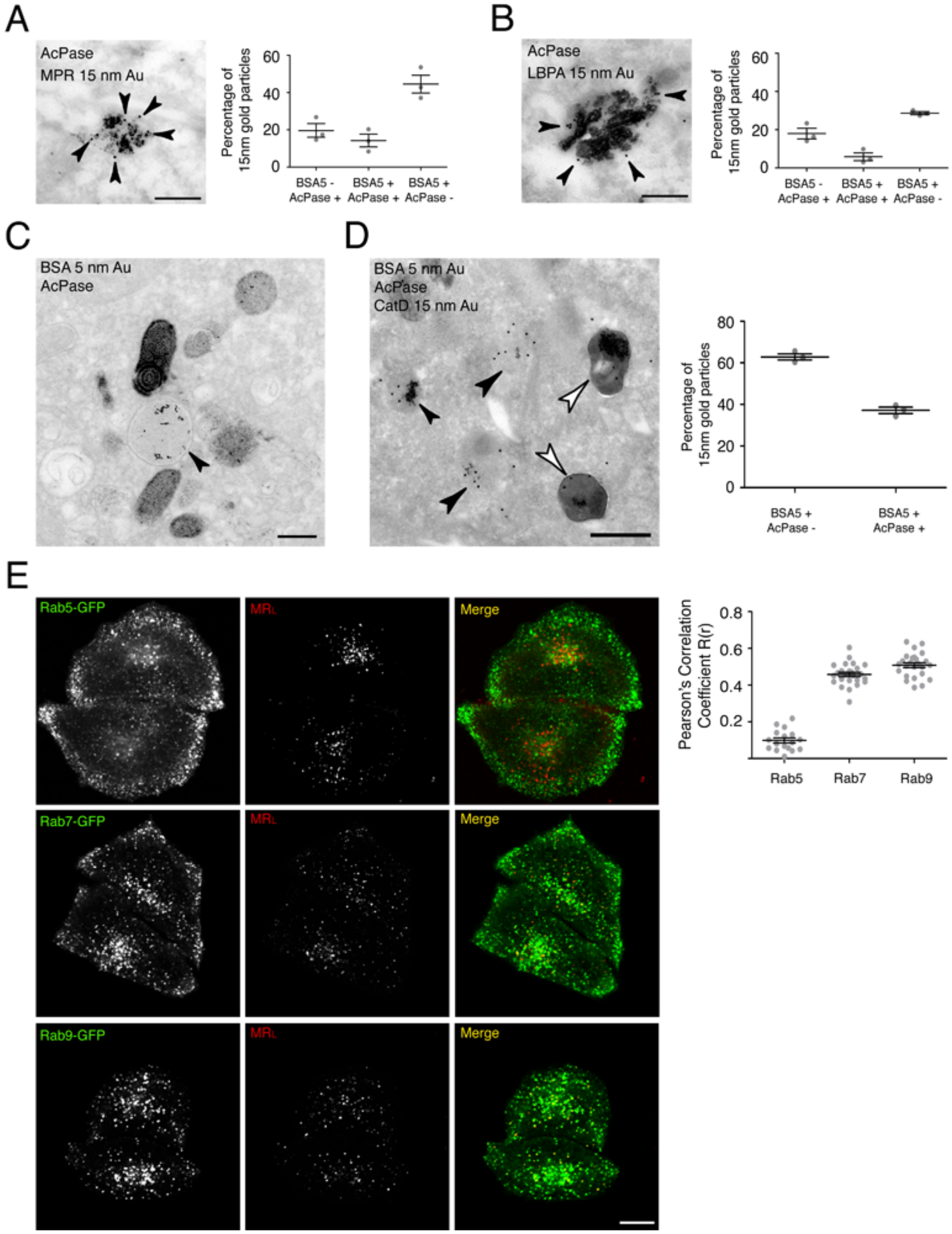

Figure S3: Characterisation of endolysosomes. Related to Figure 3. Terminal endocytic compartments of NRK (A-C) and MCF7 (D) cells were loaded with BSA-gold as for Figure 3. **A-B)** Immuno-EM of NRK cells in which the presence of active AcPase was revealed by cytochemistry, with cytidine 5'-monophosphate and lead nitrate capture for 1 h, showed that the AcPase-active organelles labelled with lead phosphate deposits were also immunolabelled with **A**) anti-MPR (15 nm colloidal gold, arrowheads) and **B**) anti-LBPA (15 nm colloidal gold, arrowheads). Graphs show distribution of label (in 3 experiments 1729 gold particles counted for MPR and 1860 for LBPA). **C)** TEM of NRK cells in which the presence of active AcPase was revealed by cytochemistry using β-glycerophosphate and cerium chloride capture, revealed the presence of 5 nm gold-positive organelles that were AcPase-negative (black arrowhead). **D)** Immuno-EM of MCF7 cells (active AcPase was revealed by cytochemistry as in panel C), showed that both AcPase-active (white arrowheads) and inactive (black arrowheads) organelles were immunolabelled with anti-cathepsin D (15 nm colloidal gold). Graph shows the distribution of label (in 3 experiments 1592 gold particles counted). **E)** Confocal immunofluorescence microscopy images and colocalization analysis (graph shows Pearson's (R(r) correlation coefficients) of HeLa cells stably expressing either GFP-labelled Rab5, Rab7 or Rab9 after 2 min incubation with cathepsin L MR substrate (MR_L). Scale Bars: A-C) 200 nm, D) 500 nm, E) 10 μm.

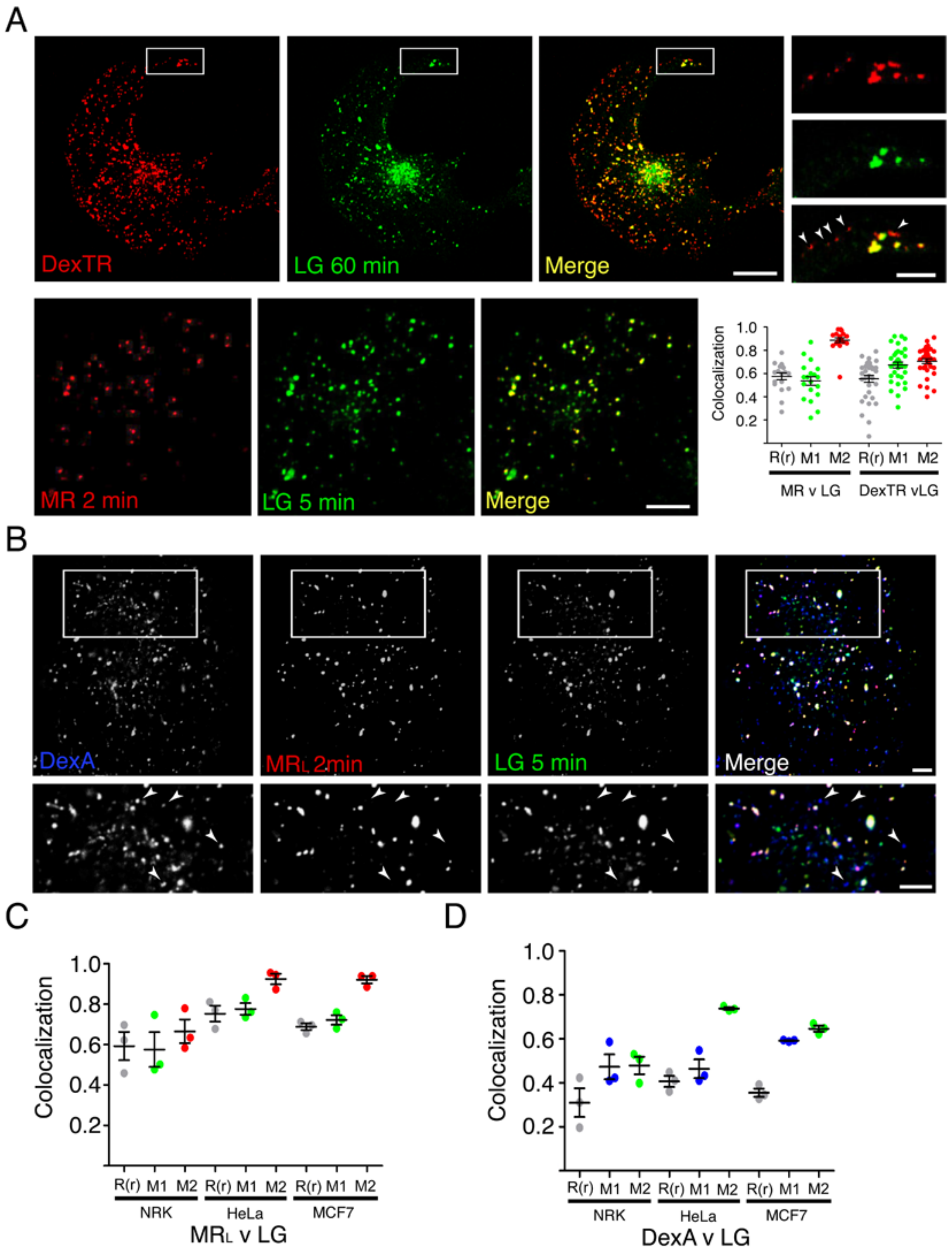

Figure S4: Cathepsin-active endolysosomes accumulate acidotropic molecules but terminal lysosomes do not. Related to Figure 4. **A**) Confocal fluorescence microscopy of NRK cells, pre-loaded with DexTR, to label terminal endocytic compartments, and incubated for 1 h with 50 nM LysoTracker^{TM} Green (LG; upper panel) or with Magic Red B substrate (MR) for 2 min followed by 50 nM LysoTracker^{TM} Green (LG) for 5 min (lower panel). An enlargement of the boxed region in the cell shown in the upper panel shows DexTR-loaded terminal endocytic compartments that did not accumulate the acidotropic molecule (arrowheads). Pearson's (R(r); gray) and Manders' (M1 and M2) correlation coefficients for colocalization of LG and MR or DexTR are shown (mean ± SEM of ≥ 17 cells). M1(left), green, LG:MR; M2 (left), red, MR:LG; M1 (right), green, LG:DexTR; M2 (right), red, DexTR:LG. **B)** Enlarged confocal immunofluorescence microscopy images of NRK cells pre-loaded with DexA, to label terminal endocytic compartments, and incubated for 2 min with the cathepsin L MR substrate (MR_L) followed by addition of 50 nM LysoTracker^{TM} Green (LG) for 5 min. Arrowheads show DexA-positive terminal endocytic organelles that are both MR_L-negative and LG-negative (correlation coefficients for colocalization are shown in Figure S1E). **C)** Pearson's (R(r); gray) and Manders' (M1, green, LG:MR and M2, red, MR:LG)) correlation coefficients for colocalization of MR_L and LG in NRK, HeLa or MCF7 cells (mean ± SEM, 3 separate experiments). **D)** Pearson's (R(r); gray) and Mander's (M1, blue, DexA:LG and M2, green, LG:DexA) correlation coefficients for colocalization of LG and DexA in NRK, HeLa or MCF7 cells (mean ± SEM (3)). Scale Bars: A) 20 μm (enlargement 5 μm), B) 5 μm.

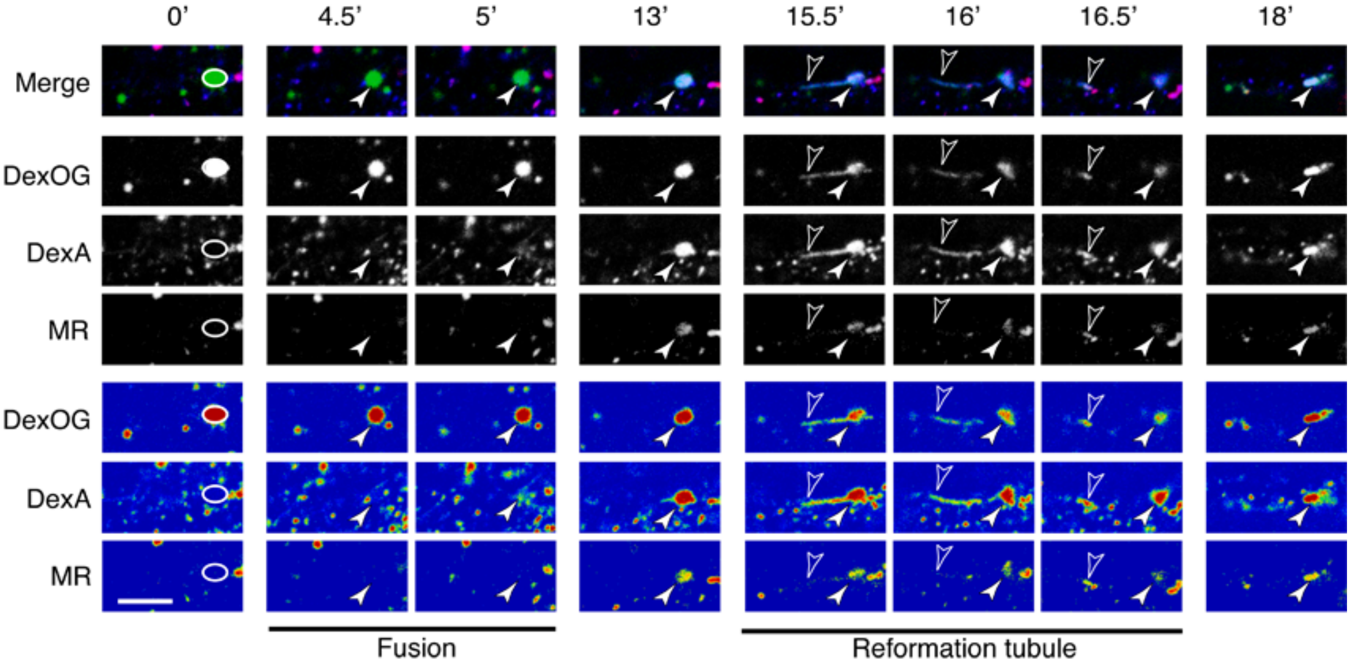

Figure S5: Emergence of tubules after formation of a cathepsin-active endolysosome. Related to Figure 5. Terminal endocytic compartments of NRK cells were pre-loaded with DexA and late endosomes loaded with DexOG and chased in medium containing cathepsin B MR substrate as for Figure 5. Timelapse confocal microscopy of a living cell showing a complete fusion between a DexOG-laden organelle and DexA-laden organelle(s) and subsequent rise in cresyl violet fluorescence from the cleaved cathepsin B MR substrate. The DexOG labelled late endocytic organelle is circled in the first panel and indicated by the solid arrowheads in subsequent panels. The false-colour images of the data (bottom 3 rows) depict background intensity (blue), low (green), intermediate (yellow) and high (red) fluorescence intensity of each of the fluorochromes. Extrusion and detachment of a reformation tubule (open arrowheads) containing each of the 3 fluorochromes was seen after 15.5 min. Scale Bar: 5 μm.

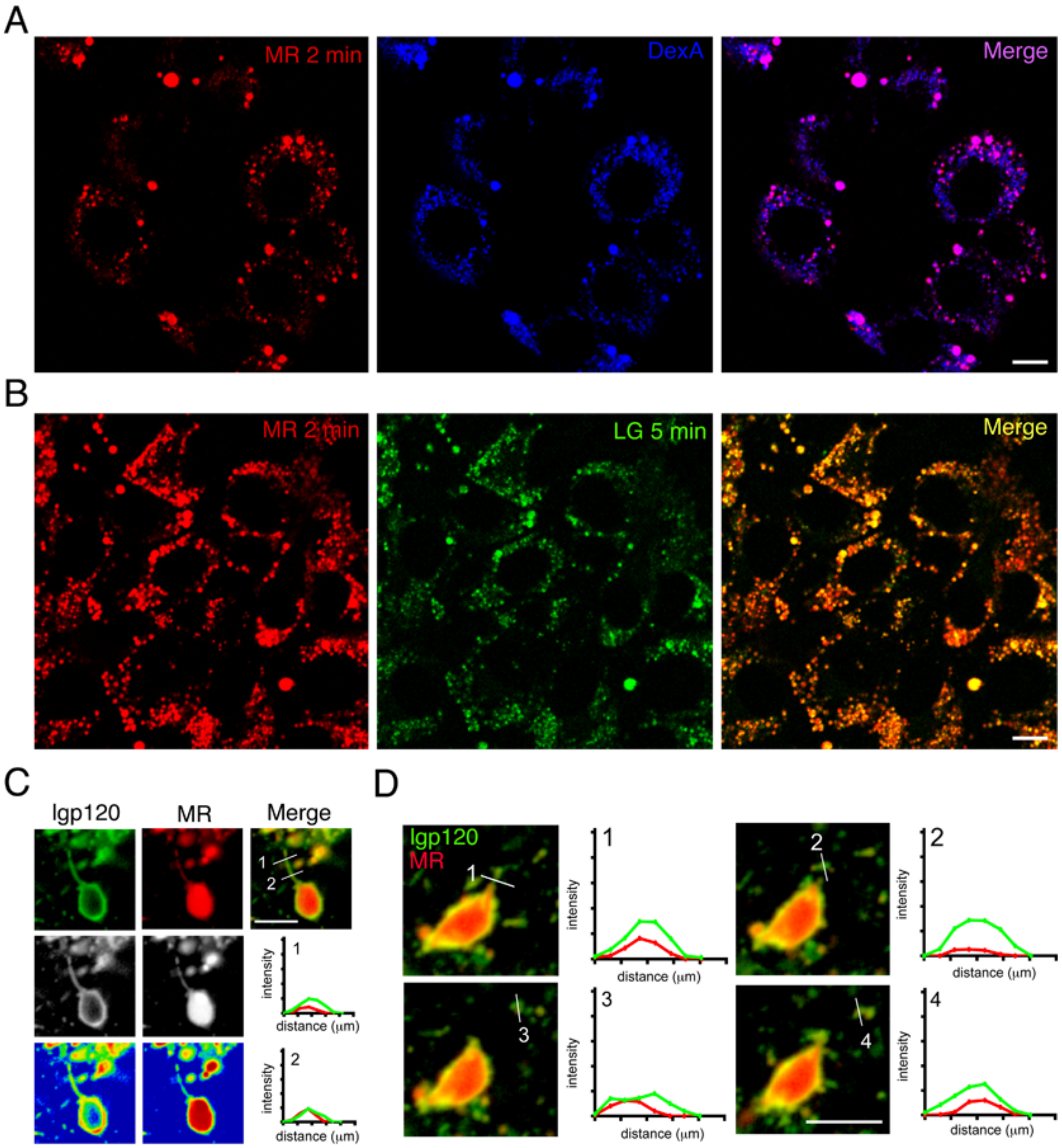

Figure S6: Sucrosomes and the re-formation of lysosomes. Related to Figure 6.

A and B. Terminal endocytic compartments of NRK cells were loaded with DexA, sucrosomes formed and cells incubated with cathepsin L MR substrate (MR_L) and LysoTracker^{TM} Green (LG) as for Figure 6. Sucrosomes (swollen vacuoles) contained DexA, were cathepsin L –active (A) and acidic (B). **C)** Sucrosomes were formed in NRK cells stably expressing lgp120-GFP as for Figure 6 and the cells subsequently incubated for 1 h in medium containing 0.5 mg/ml invertase followed by cathepsin B MR substrate for 2 min. A representative image, and pixel intensity line-scans, taken from a timelapse series demonstrate a reformation tubule containing the limiting membrane marker lgp120-GFP (green) and the cresyl violet lumenal content marker (red, MR). The false-colour images of the data (bottom panels) depict background intensity (blue), low (green), intermediate (yellow) and high (red) fluorescence intensity of each of the fluorochromes. **D)** Four sequential images and pixel intensity line-scans taken from a timelapse series of NRK cells stably expressing lgp120-GFP treated as in (C) show that the reformation tubule observed contains the limiting membrane marker lgp120-GFP (green) and the cresyl violet lumenal content marker (red, MR), both during extrusion and subsequent detachment from the parent sucrosome. Scale Bars: A-B) 10 μm, C-D) 5 μm.

Supplemental Experimental Procedures

Reagents

Dulbecco's modified Eagle's medium (DMEM), Roswell Park Memorial Institute medium (RPMI 1640), fetal calf serum (FCS), cytidine 5'-monophosphate di-sodium salt, cerium(III) chloride heptahydrate, beta-glycerophosphate di-sodium salt hydrate, Lucifer Yellow di-lithium salt, chloroquine and invertase were from Sigma-Aldrich (Poole, UK). Magic Red™ cathepsin B and L substrates were from ImmunoChemistry Technologies (Bloomington, MN USA). Glycyl-L-phenylalanine 2-naphthylamide (GPN) was from Santa Cruz Biotechnology (Dallas, USA). Nigericin (sodium salt) and monensin (sodium salt) were from the Cayman Chemical Company (Ann Arbor, MI, USA). *N*-(3-((2,4-dinitrophenyl)amino)propyl)-*N*-(3-aminopropyl)methylamine, dihydrochloride (DAMP) was from ThermoFisher Scientific (Paisley, UK). Dextran-Oregon Green 488 (DexOG; M_r 10,000, anionic, fixable), dextran-Texas Red™ (DexTR; M_r 10,000, anionic, fixable), dextran-Alexa 647 (DexA; M_r 10,000, anionic, fixable), LysoTracker™ Green, DQ™ Green BSA, rabbit anti-dinitrophenol (DNP), rabbit anti-Texas Red™, donkey anti-mouse: Alexa 594, donkey anti-rabbit: Alexa 594, donkey anti-mouse: Alexa 488, donkey anti-mouse: Alexa 647, Hoechst 33342, Geneticin(G418) and CO_2-independent medium were from ThermoFisher Scientific (Paisley, UK). Lead nitrate was from Agar Scientific (Stansted, UK). Protein A gold conjugates were from the Department of Cell Biology, University of Utrecht. BSA conjugated to 5 nm colloidal gold (BSA-gold) was prepared as described previously [S1]. The mouse monoclonal anti-cation-independent mannose-6-phosphate receptor antibody (clone 2G11) was from Abcam (Cambridge, UK), the mouse monoclonal anti-LC3 (4E12) from MBL International (Woburn, MA USA) and the rabbit anti-cathepsin D antibody was from EMD Millipore (Billerica, MA USA). The mouse monoclonal anti-LBPA antibody (clone 6C4) was a generous gift from Professor Jean Gruenberg, University of Geneva. The rabbit anti-lgp 110 antibody has been described previously [S2]. Torin 1 was from Tocris Bioscience (Bristol, UK), and bafilomycin A1 from Alfa Aesar (Heysham, UK). The TFEB-GFP construct in a pEGFP vector was a gift from Andrea Ballabio (Telethon Institute of Genetics and Medicine, Pozzuoli, NA, Italy) and was subcloned into a pLXIN retroviral expression vector (Clontech, Mountain View, CA, USA). The lgp120-GFP in pEGFP N1, with a 10 amino acid linker between the lgp120 cytosolic tail and the eGFP is available from Addgene (Cambridge, MA, USA) and was subcloned into pLXIN. HeLa cells stably expressing EGFP-tagged Rab5, Rab 7 and Rab9 [S3] were a gift from Dr Matthew Seaman (Cambridge Institute for Medical Research, UK).

Cell Culture and Transduction

NRK fibroblasts and human breast adenocarcinoma MCF7 cells were cultured in DMEM and HeLa-M epithelial cells in RPMI 1640, both media supplemented with 10% (v/v) FCS, 100 IU/ml penicillin, 100 μg/ml streptomycin, 4.5 g/l glucose, and 2 mM L-glutamine. NRK cells stably expressing TFEB-GFP or lgp120-GFP were generated using the pLXIN retroviral system as previously described [S4]. Transduced cells of mixed expression levels were selected by culture in medium supplemented with 0.5 mg/ml Geneticin (G418) and a clonal NRK cell line expressing TFEB-GFP was subsequently generated after isolating single cells by fluorescence activated cell sorting.

Cells were seeded onto 15 mm glass coverslips for fixed-cell confocal microscopy, 42 mm diameter glass coverslips (PeCon, GmbH, Germany) for live cell imaging, gridded glass bottom dishes (MatTek Ashland, MA, USA) for CLEM, 25 cm^2 tissue culture flasks for immuno-EM, glass-bottom 96-well plates for Cellomics ArrayScan™ wide-field microscopy, Deckgläser 25 mm coverslips (Techmate Ltd, Milton Keynes, UK) for ratiometric imaging and grown in a 5% CO_2 incubator at 37°C.

Incubation with cathepsin substrates and acidotropic compounds

To label endocytic organelles in which cathepsin B or cathepsin L was catalytically active, cells were incubated respectively with cathepsin B MR (Magic Red™) substrate or cathepsin L MR substrate, prepared in CO_2-independent medium / 10 % FCS, but used at a 10-fold dilution (final concentration 673 nM) compared to the manufacturers recommendations. The substrate was added directly to cells growing at 37°C on PeCon coverslips or MatTek glass bottom dishes on the incubated stage of a Zeiss confocal microscope (Carl Zeiss Ltd, Welwyn Garden City, UK) and the live cells were imaged to assay the liberation of cresyl violet fluorescence. Alternatively, cells were incubated, in a 5% CO_2 incubator, with Magic Red™ cathepsin substrates, prepared in DMEM / 10% FCS, for 2-60 min prior to imaging. GPN was prepared as a 200 mM stock in dimethyl sulphoxide and used at a final concentration of 200 μM.

LysoTracker™ Green was prepared in CO_2-independent medium / 10 % FCS and added to cells on PeCon coverslips or MatTek glass bottom dishes at a final concentration of 50 nM for 5 min at 37°C. Alternatively, cells were incubated in 5% CO_2 incubator in the presence of 50 nM LysoTracker™ Green prepared in DMEM / 10% FCS for 60 min prior to imaging. DAMP was made up as a 100 mM stock solution in H_2O and stored at -20°C before application to cells at 30 μM in DMEM for 30 min at 37°C.

pH clamping

Nigericin and monensin were reconstituted in ethanol as 10 mM stock solutions and stored at -20°C. pH clamping was by modification of a previously described method [S5]. A pH 5 clamping solution was prepared using 25 mM sodium acetate buffer, pH 5 containing 5 mM NaCl, 1 mM $CaCl_2$, 115 mM KCl, 1.2 mM $MgSO_4$, 10 mM glucose, 10 μM nigericin and 10 μM monensin. For fluorescence confocal microscopy, NRK cells in which DexA had been endocytosed for 4 h followed by a 24 h chase were incubated with the pH 5 clamping solution containing 637 nM cathepsin B MR substrate for 5 min.

Fluorescence microscopy

For immunofluorescence microscopy cells were fixed in 4% (w/v) paraformaldehyde in phosphate buffered saline pH 7.4 (PBS) for 10 min and permeabilized with 0.05% (w/v) saponin for 5 min at 20°C. For cytosolic washout cells were rinsed with PBS and incubated with PBS containing 0.05% (w/v) saponin for 30 s at 20°C before fixing with 4% (w/v) paraformaldehyde. All antibody incubations were carried out in PBS with 0.1% (w/v) BSA and 5% (v/v) FCS. Secondary antibodies were Alexa Fluor conjugates used at a final concentration of 4 μg/ml. Images of immunolabelled cells were collected with a x63 1.4 NA Plan Apochromat oil-immersion lens on Zeiss LSM 710, 780 or 880 confocal microscopes. Nuclear translocation of TFEB-GFP in NRK cells stably expressing TFEB-GFP was quantified after fixing with 4% (w/v) paraformaldehyde and staining nuclei with Hoechst 33342 using a Cellomics ArrayScan^{TM} VTi high content screening, wide-field microscope. Images of nuclear translocation were obtained using a Zeiss LSM 780 confocal microscope.

Rapid immunofluorescence protocol for cells incubated with cathepsin MR substrates

A rapid immunolabelling protocol was used to detect antigens in cells in which cresyl violet had been liberated from cathepsin MR substrate, before it dissipated from labelled organelles. Cells were grown on Mattek dishes, incubated with DMEM containing cathepsin MR substrate as described above and fixed with 4% PFA (w/v) in PBS for 5 min at 37°C. Cells were then permeabilised with 0.05% saponin in PBS for 5 min at room temperature, washed with PBS and incubated with 100 μl primary antibody diluted in PBS containing 5% (v/v) FCS and 0.1% (w/v) BSA for 10 min at room temperature. The cells were then washed and incubated with secondary fluorochrome-conjugated antibodies diluted in PBS containing 5% (v/v) FCS and 0.1% (w/v) BSA for 10 min at room temperature. The cells were washed and viewed immediately on the confocal microscope. Using this protocol sufficient cresyl violet was retained to demonstrate positive colocalizations but not for robust quantitation since the cresyl violet had begun to dissipate.

Ratiometric imaging

Dual-fluorochrome ratiometric imaging was performed utilising Oregon Green 488 as the pH-sensitive fluorochrome and Alexa 647 as the pH-insensitive fluorochrome. NRK cells cultured on Deckgläser 25 mm coverslips were incubated in DMEM containing 0.5 mg/ml DexOG and 0.5 mg/ml DexA for 4 h followed by a 20 h chase in conjugate-free DMEM to load terminal endocytic compartments as described above. The coverslips were placed in an Attofluor® Cell Chamber (ThermoFisher Scientific, Paisley, UK), incubated with Magic Red^{TM} cathepsin B substrate as described above and placed on the incubated stage of a Zeiss LSM 780 confocal microscope. To construct a pH calibration curve, cells were incubated with 25 mM sodium acetate buffer, pH 4 or pH 5, 25 mM MES buffer, pH 6 and 25 mM Hepes buffer, pH 7 containing 5 mM NaCl, 1 mM $CaCl_2$, 115 mM KCl, 1.2 mM $MgSO_4$, 10 mM glucose, 10 μM nigericin and 10 μM monensin for 5 min. Images were collected as described above and the mean background-corrected A647/OG488 fluorescence ratio was calculated at each pH. We recorded 12 images and selected a total of 300 regions of interest using NIH Image J (v1.48r) to quantify individual terminal endocytic organelles using the Alexa 647 channel. The background-corrected A647/OG488 ratio of each organelle was used to calculate the pH by interpolation against the calibration curve and plotted against the mean fluorescence intensity of cresyl violet liberated from cleavage of Magic Red^{TM} cathepsin B in each organelle.

Live Cell Imaging

Cells were seeded onto 42 mm diameter glass coverslips (PeCon, GmbH, Germany), inserted into a POC cell chamber and incubated at 37°C. They were then transferred to the incubated stage of an LSM710, 780 or 880 confocal microscope (Carl Zeiss, Ltd., Welwyn Garden City, UK). Live cells were imaged using a x63 1.4 NA Plan Apochromat oil-immersion lens using an Argon laser line at 488 nm (used to excite Oregon Green 488 or EGFP), a HeNe laser line at 543 nm (used to excite Texas Red^{TM} or cresyl violet) and a HeNe laser line at 633 nm (used to excite Alexa 647). Band pass (505–530 nm) and long pass (560 nm) filters were used to separate emission wavelengths of Oregon Green 488 and Texas Red^{TM} respectively. Laser power was attenuated to 2% of maximum to minimize photobleaching and phototoxicity. The detector pinholes were set to give a 0.7-1.4 μm optical slice. Pixel dwell times varied from 1μs to 1.58μs using multitracking (line switching) with a line average of 4. Acquisition was performed using Zen software (Zen 2012 black edition v 8.1) and processing was completed using Adobe Photoshop CS4 (version 11). Time-lapse images of the living cells were recorded every 2-30s and movies were assembled using Adobe Illustrator CS4 (version 14) and NIH Image J (v1.48r).

Acid Phosphatase Cytochemistry

NRK cells were grown in MatTek glass bottom dishes or 25 cm^2 tissue culture flasks and were first incubated with BSA-gold for 4 h followed by a 20 h chase in conjugate-free medium to label terminal endocytic compartments. For TEM of resin-embedded material, cells grown on MatTek glass bottom dishes were fixed by addition of 2% (wt/vol) paraformaldehyde / 2.5% (vol/vol) glutaraldehyde in 0.1 M sodium cacodylate, pH 7.2, to an equal volume of tissue culture medium at 37°C for 1 h. For TEM of cells prepared for immunogold electron microscopy, cells were fixed by addition of 8% (wt/vol) paraformaldehyde / 0.2% (vol/vol) glutaraldehyde in 0.1 M sodium cacodylate, pH 7.2, to an equal volume of tissue culture medium at 37°C for 1 h. The cells were then washed with 0.1 M cacodylate buffer, pH 7.2.

AcPase cytochemistry was performed according to principles established over 50 years ago [S6-S8] using a modified procedure with cerium chloride as a capture reagent for liberated inorganic phosphate [S9]. Cells were incubated for 2 x 15 min with 0.1 M acetate buffer, pH 5, containing 2 mM cerium chloride at 37°C and then for 2 x 60 min with 0.1 M acetate buffer, pH 5, containing 2 mM cerium chloride and 5 mM sodium β-glycerophosphate. The cells were then washed and processed for TEM as described below. For immuno-EM, cells were either processed as described above with cerium chloride or incubated for 2 x 15 min with 0.1 M acetate buffer, pH 5 containing 3.6 mM lead nitrate at 37°C and then for 2 x 30 min with 0.1 M acetate buffer, pH 5 containing 3.6 mM lead nitrate and 1 mM cytidine 5'-monophosphate. The cells were then washed and processed for immuno-EM as described below.

Correlative Light and Electron Microscopy

After visualization of living cells growing on MatTek gridded, glass bottom dishes by confocal microscopy they were fixed by addition of 4% paraformaldehyde / 5% glutaraldehyde in 0.1 M Na cacodylate buffer (pH 7.2) to an equal volume of culture medium. The position of the cells was recorded and confocal Z-series and DIC images were acquired to enable subsequent identification of the cells by TEM. After incubation for 5 min at 37°C, the solution was replaced with 2% paraformaldehyde / 2.5% glutaraldehyde in 0.1 M Na cacodylate buffer (pH 7.2). Cells were processed for TEM as detailed below, embedded in Agar 100 resin, cured, and the glass coverslip was removed by immersion in liquid nitrogen. Ultrathin sections (50 nm) were cut "en face" to the plane of the coverslip and mounted on formvar/carbon-coated slot EM grids.

TEM

Cells were fixed with 2% (wt/vol) paraformaldehyde / 2.5% (vol/vol) glutaraldehyde in 0.1 M sodium cacodylate buffer, pH 7.2, at 37°C, post fixed with 1% OsO_4 in 0.1 M sodium cacodylate buffer, pH 7.2, 'en bloc' stained with 0.5% (wt/vol) uranyl acetate in 50 mM sodium maleate buffer, pH 5.2 and processed for TEM as previously described [S1]. Cell monolayers were embedded in Agar 100 resin (Agar Scientific, Stansted, United Kingdom) by inverting a resin-filled BEEM capsule over them, polymerised and the coverslip was removed by immersion in liquid nitrogen. Ultrathin sections (50 nm) were cut parallel to the plane of the coverslip and mounted on formvar/carbon-coated slot EM grids. Sections for CLEM were stained with uranyl acetate and lead citrate and sections for AcPase cytochemistry were viewed unstained. Sections were examined with an FEI Tecnai G2 Spirit BioTwin transmission electron microscope (Eindhoven, The Netherlands) at an operating voltage of 80 kV and images were recorded with an Eagle 4K CCD camera.

Immuno-EM

Cells were fixed with 4% (wt/vol) paraformaldehyde / 0.1% (vol/vol) glutaraldehyde in 0.1 M sodium cacodylate buffer (pH 7.2), and pelleted in an Eppendorf tube (11,000 rpm for 5 min). The fixative was aspirated, and the cell pellet was resuspended in warm 12% (wt/vol) gelatin in PBS. The cells were then pelleted (11,000 rpm for 5 min) and the gelatin-enrobed cells were set on ice, trimmed into 1 mm^3 blocks and infused with 1.7 M sucrose / 15% (wt/vol) polyvinylpyrolidone for 24 h at 4°C. The blocks were subsequently mounted on cryostubs and snap-frozen in liquid nitrogen. Frozen ultrathin sections were cut using a diamond knife in an ultramicrotome with a cryochamber attachment (Leica, Milton Keynes UK) at −120°C, collected from the knife-edge with 50:50 2% (wt/vol) methyl cellulose: 2.3 M sucrose [S10] and mounted on formvar-carbon–coated EM grids.

Immunolabeling of the cation-independent mannose 6-phosphate receptor, LBPA, DAMP, cathepsin D and Texas Red™ was performed using the protein A-gold technique at room temperature [S11] as described previously [S1, S12]. The sections were contrasted by embedding in 1.8% (wt/vol) methyl cellulose / 0.3% (wt/vol) uranyl acetate and air-dried before observation in a TEM as described above.

Quantitative analysis of the subcellular distribution of gold particles was as described previously [S1].

Quantitation of DAMP labelled organelles

Quantitation of the labelling density of DAMP-labelled organelles was performed using iTEM software (Olympus Soft Imaging Solutions, Melville, NY). EM blocks, grids, and sections were selected randomly, sections were scanned systematically and an Eagle 4K CCD camera (FEI, Einhoven, The Netherlands) was used to record images. In NRK cells in which terminal endocytic compartments were pre-loaded with dextran-Texas Red™ (DexTR), the density of DAMP

immunolabelling was recorded in 71 individual organelles co-immunolabelled with anti-TR antibodies (2012 gold particles) and 49 nuclei (2020 gold particles).

Quantitation of fluorescence microscopy

Fluorescence colocalization analysis was carried out with Imaris software (Imaris x64 v7.7.1, Bitplane Scientific Software, Zürich, Switzerland) using its automated thresholding algorithm on confocal images with detectors adjusted such that the peak intensity in each channel was just below saturation. Pearson's correlation coefficients and Manders' colocalization coefficients were calculated and used respectively to measure the overall association of two fluorescent probes and the fraction of the total fluorescence of one probe overlapping with a second [S13, S14]. The size distribution of organelles (Figure S1B) in 15 NRK cells from a single experiment was analysed with the Imaris software. The MR-negative, DexA-positive organelles were identified as 35.5% of the total of 2447 DexA-positive organelles, based on Manders'coefficient (M1) data shown in Figure S1D. None of these organelles had MR-channel fluorescence >1.5x maximum background fluorescence. Quantitation of LC3 fluorescence intensity to assess autophagy was carried out as previously described [S15]. To quantify nuclear translocation of TFEB-GFP, Cellomics ArrayScan™ software was used. After autofocussing using the Hoechst 33342 channel to detect the nuclei, the programme maps and outlines the area of each nucleus (Nuc) and a region extending outwards by an adjustable number of pixels (Cyt), representing a significant portion of the cytoplasm). Nuclear translocation (NucCyt difference) is calculated as the mean intensity of pixels in Nuc after subtracting the mean intensity of pixels in Cyt.

Supplemental References

S1. Bright, N.A., Reaves, B.J., Mullock, B.M., and Luzio, J.P. (1997). Dense core lysosomes can fuse with late endosomes and are re-formed from the resultant hybrid organelles. J Cell Sci *110 (Pt 17)*, 2027-2040.

S2. Reaves, B.J., Bright, N.A., Mullock, B.M., and Luzio, J.P. (1996). The effect of wortmannin on the localisation of lysosomal type I integral membrane glycoproteins suggests a role for phosphoinositide 3-kinase activity in regulating membrane traffic late in the endocytic pathway. J Cell Sci *109 (Pt 4)*, 749-762.

S3. Seaman, M.N., Harbour, M.E., Tattersall, D., Read, E., and Bright, N. (2009). Membrane recruitment of the cargo-selective retromer subcomplex is catalysed by the small GTPase Rab7 and inhibited by the Rab-GAP TBC1D5. J Cell Sci *122*, 2371-2382.

S4. Gordon, D.E., Mirza, M., Sahlender, D.A., Jakovleska, J., and Peden, A.A. (2009). Coiled-coil interactions are required for post-Golgi R-SNARE trafficking. EMBO Rep *10*, 851-856.

S5. Lange, P.F., Wartosch, L., Jentsch, T.J., and Fuhrmann, J.C. (2006). ClC-7 requires Ostm1 as a beta-subunit to support bone resorption and lysosomal function. Nature *440*, 220-223.

S6. Gomori, G. (1952). Microscopic histochemistry: principles and practice, (University of Chicago Press).

S7. Essner, E., and Novikoff, A.B. (1961). Localization of acid phosphatase activity in hepatic lysosomes by means of electron microscopy. J Biophys Biochem Cytol *9*, 773-784.

S8. Holt, S.J., and Hicks, R.M. (1961). The localization of acid phosphatase in rat liver cells as revealed by combined cytochemical staining and electron microscopy. J Biophys Biochem Cytol *11*, 47-66.

S9. Robinson, J.M., and Karnovsky, M.J. (1983). Ultrastructural localization of several phosphatases with cerium. J Histochem Cytochem *31*, 1197-1208.

S10. Liou, W., Geuze, H.J., and Slot, J.W. (1996). Improving structural integrity of cryosections for immunogold labeling. Histochem Cell Biol *106*, 41-58.

S11. Slot, J.W., and Geuze, H.J. (2007). Cryosectioning and immunolabeling. Nat Protoc *2*, 2480-2491.

S12. Bright, N.A., Gratian, M.J., and Luzio, J.P. (2005). Endocytic delivery to lysosomes mediated by concurrent fusion and kissing events in living cells. Curr Biol *15*, 360-365.

S13. Dunn, K.W., Kamocka, M.M., and McDonald, J.H. (2011). A practical guide to evaluating colocalization in biological microscopy. Am J Physiol Cell Physiol *300*, C723-742.

S14. Manders, E.M.M., Verbeek, F.J., and Aten, J.A. (1993). Measurement of Colocalization of Objects in Dual-Color Confocal Images. J Microsc-Oxford *169*, 375-382.

S15. Wartosch, L., Gunesdogan, U., Graham, S.C., and Luzio, J.P. (2015). Recruitment of VPS33A to HOPS by VPS16 Is Required for Lysosome Fusion with Endosomes and Autophagosomes. Traffic *16*, 727-742.
